# A Unified Model for Stress-Driven Rearrangement Instabilities

**DOI:** 10.1007/s00205-020-01546-y

**Published:** 2020-06-20

**Authors:** Shokhrukh Yu. Kholmatov, Paolo Piovano

**Affiliations:** Fakultät für Mathematik, Universität Wien, Oskar-Morgenstern Platz 1, 1090 Wien, Austria

## Abstract

A variational model to simultaneously treat Stress-Driven Rearrangement Instabilities, such as boundary discontinuities, internal cracks, external filaments, edge delamination, wetting, and brittle fractures, is introduced. The model is characterized by an energy displaying both elastic and surface terms, and allows for a unified treatment of a wide range of settings, from epitaxially-strained thin films to crystalline cavities, and from capillarity problems to fracture models. The existence of minimizing configurations is established by adopting the direct method of the Calculus of Variations. The compactness of energy-equibounded sequences and energy lower semicontinuity are shown with respect to a proper selected topology in a class of admissible configurations that extends the classes previously considered in the literature. In particular, graph-like constraints previously considered for the setting of thin films and crystalline cavities are substituted by the more general assumption that the free crystalline interface is the boundary, consisting of an at most fixed finite number *m* of connected components, of sets of finite perimeter. Finally, it is shown that, as $m\rightarrow \infty$, the energy of minimal admissible configurations tends to the minimum energy in the general class of configurations without the bound on the number of connected components for the free interface.

## Introduction

Morphological destabilizations of crystalline interfaces are often referred to as Stress-Driven Rearrangement Instabilities (SDRI), from the seminal paper [40] (see also Asaro-Grinfeld-Tiller instabilities [4, 21]). SDRI consist in various mechanisms of mass rearrangements that take place at crystalline boundaries because of the strong stresses originated by the mismatch between the parameters of adjacent crystalline lattices. Atoms move from their crystalline order and different modes of stress relief may co-occur, such as deformations of the bulk materials with storage of *elastic energy*, and boundary instabilities that contribute to the *surface energy*.

In this paper we introduce a variational model displaying both elastic and surface energy that simultaneously takes into account the various possible SDRI, such as *boundary discontinuities*, internal *cracks*, external *filaments*, *wetting* and *edge delamination* with respect to a substrate, and *brittle fractures*. In particular, the model provides a unified mathematical treatment of epitaxially-strained thin films [22, 31, 33, 42, 48], crystal cavities [30, 47, 49], capillary droplets [11, 24, 26], as well as Griffith and failure models [9, 13, 14, 39, 50], which were previously treated separately in the literature. Furthermore, the possibility of delamination and debonding, i.e., crack-like modes of interface failure at the interface with the substrate [27, 41], is treated in accordance with the models in [5, 43, 44], that were introduced by revisiting in the variational perspective of fracture mechanics the model first described in [50]. Notice that as a consequence the surface energy depends on the admissible deformations and cannot be decoupled from the elastic energy. As a byproduct of our analysis, we extend previous results for the existence of minimal configurations to anisotropic surface and elastic energies, and we relax constraints previously assumed on admissible configurations in the thin-film and crystal-cavity settings. For thin films we avoid the reduction considered in [22, 23, 31] to only film profiles parametrizable by thickness functions, and for crystal cavities the restriction in [30] to cavity sets consisting of only one connected starshaped void.

The class of interfaces that we consider is given by all the boundaries, that consists of connected components whose number is arbitrarily large but not exceeding a fixed number *m*, of sets of finite perimeter *A*. We refer to the class of sets of finite perimeter associated to the free interfaces as *free crystals* and we notice that free crystals *A* may present an infinite number of components. The assumption on the number of components for the boundaries of free crystals is needed to apply an adaptation to our setting of the generalization of Golab’s Theorem proven in [36] that allows one to establish in dimension 2, to which we restrict, compactness with respect to a proper selected topology. To the best of our knowledge, no variational framework able to guarantee the existence of minimizers in dimension 3 in the settings of thin films and crystal cavities is available in the literature.

**Fig. 1:**
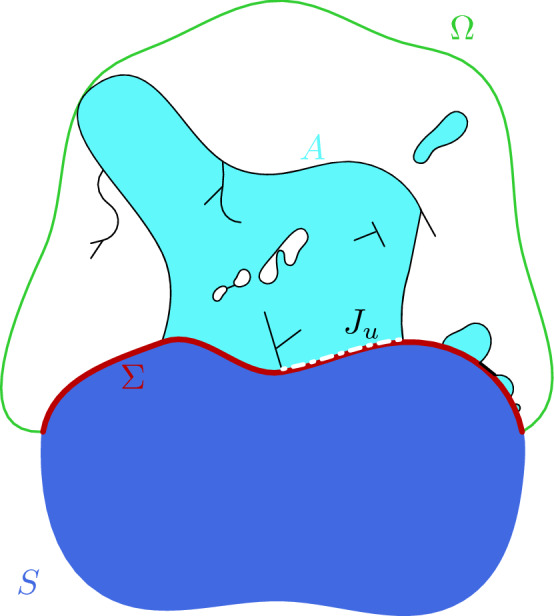
An admissible free (disconnected) crystal *A* is displayed in light blue in the container $\Omega$, while the substrate *S* is represented in dark blue. The boundary of *A* (with the cracks) is depicted in black, the container boundary in green, the contact surface $\Sigma$ in red (thicker line) while the delamination region $J_u$ with a white dashed line

Furthermore, the class of admissible deformations is enlarged with respect to [22, 23, 30, 31] to allow debonding and edge delamination to occur along the *contact surface*$\Sigma :=\partial S\cap \partial \Omega$ between the fixed substrate *S* and the fixed bounded region $\Omega$ containing the admissible free crystals (see Fig. 1). In what follows we refer to $\Omega$ as the *container* in analogy with capillarity problems. Notice that the obtained results can be easily applied also for unbounded containers in the setting of thin films with the graph constraint (see Section 2.2). Mathematically this is modeled by considering admissible deformations *u* that are Sobolev functions only in the interior of the free crystals *A* and the substrate *S*, and *GSBD*, i.e., generalized special functions of bounded deformation (see [19] for more details), on $A\cup S\cup \Sigma$. Thus, jumps $J_u$ that represent edge delamination can develop at the contact surface $\Sigma$, i.e., $J_u\subset \Sigma$.

The energy $\mathcal {F}$ that characterizes our model is defined for every admissible configuration (*A*, *u*) in the configurational space $\mathcal {C}_m$ of free crystals and deformations by

$$\begin{aligned} \mathcal {F}(A,u):=\mathcal {S}(A,u) + \mathcal {W}(A,u), \end{aligned}$$

where $\mathcal {S}$ denotes the *surface energy* and $\mathcal {W}$ the *bulk elastic energy*. The bulk elastic energy is given by

$$\begin{aligned} \mathcal {W}(A,u)=\int _{A\cup S} W(z,e(u)-E_0)\,\text {d}z \end{aligned}$$

for an elastic density $W(z,M):=\mathbb {C}(z) M:M$ defined with respect to a positive-definite elasticity tensor $\mathbb {C}$ and a *mismatch strain*$E_0$. The mismatch strain is introduced to represent the fact that the lattice of the free crystal generally does not match the substrate lattice. We notice that the tensor $\mathbb {C}$ is assumed to be only $L^\infty (\Omega \cup S)$, therefore not only allowing for different elastic properties between the material of the free crystals in $\Omega$ and the one of the substrate, but also for non-constant properties in each material extending previous results. The surface energy $\mathcal {S}$ is defined as

$$\begin{aligned} \mathcal {S}(A,u)= \int _{\partial A} \psi (z,u,\nu )\,\mathrm{d}\mathcal {H}^{d-1}, \end{aligned}$$

with surface tension $\psi$ defined by

1.1 $$\begin{aligned} \psi (z,u,\nu ):={\left\{ \begin{array}{ll} \varphi (z,\nu _A(z)) &{} z\in \Omega \cap \partial ^*A,\\ 2 \varphi (z,\nu _A(z)) &{} z\in \Omega \cap (A^{(1)}\cup A^{(0)})\cap \partial A,\\ \varphi (z,\nu _S(z)) + \beta (z) &{} \Sigma \cap A^{(0)}\cap \partial A,\\ \beta (z) &{} z\in \Sigma \cap \partial ^*A{\setminus } J_u,\\ \varphi (z,\nu _S(z)) &{} J_u, \end{array}\right. } \end{aligned}$$

where $\varphi \in C(\overline{\Omega }\times \mathbb {R}^d;[0,+\infty ))$ is a Finsler norm representing the *material anisotropy* with $c_1|\xi | \leqq \varphi (x,\xi ) \leqq c_2|\xi |$ for some $c_1,c_2>0$, $\beta \in L^\infty (\Sigma )$ is the *relative adhesion coefficient* on $\Sigma$ with

1.2 $$\begin{aligned} |\beta (z)|\leqq \varphi (z,\nu _S(z)) \end{aligned}$$

for $z\in \Sigma$, $\nu$ is the exterior normal on the reduced boundary $\partial ^*A$, and $A^{(\delta )}$ denotes the set of points of *A* with density $\delta \in [0,1]$. Notice that the anisotropy $\varphi$ is counted double on the sets $A^{(1)}\cap \partial A\cap \Omega$ and $A^{(0)}\cap \partial A\cap \Omega$, that represent the set of cracks and the set of external filaments, respectively. On the free profile $\partial ^*A$ the anisotropy is weighted the same as on the delamination region $J_u$, since delamination involves debonding between the adjacent materials by definition. Furthermore, the adhesion coefficient $\beta$ is considered on the contact surface $\Sigma$, alone on the reduced boundary $\Sigma \cap \partial ^*A{\setminus } J_u$ and together with $\varphi$ on those external filaments $A^{(0)}\cap \partial A\cap \Sigma$, to which we refer as *wetting layer*.

We refer the Reader to Section 2.3 for the rigorous mathematical setting and the main results of the paper, among which we recall here the following existence result:

*Main TheoremIf*$\mathtt {v}\in (0,|\Omega |)$*or*$S=\emptyset ,$*then for every*$m\geqq 1$*the volume-constrained minimum problem*

$$\begin{aligned} \inf \limits _{(A,u)\in \mathcal {C}_m,\,\,|A| = \mathtt {v}} \mathcal {F}(A,u) \end{aligned}$$

*admits a solution and*

1.3 $$\begin{aligned} \inf \limits _{(A,u)\in \mathcal {C},\,\,|A|=\mathtt {v}} \mathcal {F}(A,u) = \lim \limits _{m\rightarrow \infty } \inf \limits _{(A,u)\in \mathcal {C}_m, \,\,|A|=\mathtt {v}} \mathcal {F}(A,u). \end{aligned}$$

This existence result is accomplished in Theorem 2.6, where we also solve the related unconstraint problem with energy $\mathcal {F}^\lambda$ given by $\mathcal {F}$ plus a *volume penalization* depending on the parameter $\lambda >0.$

The proof is based on the *direct method* of the Calculus of Variations, i.e., it consists in determining a suitable topology $\tau _\mathcal {C}$ in $\mathcal {C}_m$ sufficiently weak to establish the compactness of energy-equibounded sequences in Theorem 2.7 and strong enough to prove that the energy is lower semicontinuous in Theorem 2.8. We notice here that Theorems 2.7 and 2.8 can also be seen as an extension, under the condition on the maximum admissible number *m* of connected components for the boundary, of the compactness and lower semicontinuity results in [15] to anisotropic surface tensions and to the other SDRI settings.

The topology $\tau _{\mathcal {C}}$ selected in $\mathcal {C}$ corresponds, under the uniform bound on the length of the free-crystal boundaries, to the convergence of both the free crystals and the free-crystal complementary sets with respect to the Kuratowski convergence and to the pointwise convergence of the displacements. In [22, 23, 31] the weaker convergence $\tau _{\mathcal {C}}'$ consisting of only the Kuratowski convergence of complementary sets of free-crystals (together with the $\overline{S}$) was considered, which in our setting without graph-like assumptions on the free boundary is not enough because not closed in $\mathcal {C}_m$. Working with the topology $\tau _{\mathcal {C}}$ also allows as to keep track or the surface energy of the possible external filaments of the admissible free crystals, which were in previous results not considered. However, to establish compactness with respect to $\tau _{\mathcal {C}}$ the Blaschke Selection Theorem employed in [22, 23, 30, 31] is not enough, and a version for the signed distance functions from the free boundaries is obtained (see Proposition 3.1). Furthermore, in order to take in consideration the situation in which connected components of $A_k$ separates in the limit in multiple connected components of *A*, e.g., in the case of neckpinches, we need to introduce extra boundary in $A_k$ in order to divide their components accordingly (see Proposition 3.6). Otherwise, adding to $u_k$ different rigid displacements with respect to the components in *A* (which are needed for compactness of $u_k$) would results in jumps for the displacements in $A_k$, which are not allowed in our setting with $H^1_{\mathrm{loc}}$-displacements. Therefore, we pass from the sequence $A_k$ to a sequence $D_k$ with such extra boundary for which we can prove compactness. Passing to $D_k$ is not a problem in the existence in view of property (2.9) that relates the *liminf* of the energy with respect to $A_k$ to the one with respect to $D_k$. However, in case $S\ne \emptyset ,$ in order to prove (2.9), we need to further modify the sequence $D_k$ from the original $A_k$ by cutting out the portion converging to delamination regions (e.g., portion containing accumulating cracks and voids at the boundary with *S*) using Proposition 3.9, and, in order to maintain the volume constraint, by replacing them with an extra set that does not contribute to the overall elastic energy.

The lower semicontinuity of the energy with respect to $\tau _{\mathcal {C}}$ is established for the elastic energy as in [31] by convexity, and for the surface energy in Proposition 4.1 in several steps by adopting a blow-up method (see, e.g., [1, 8]). More precisely, given a sequence of configurations $(A_k,u_k)\in \mathcal {C}_m$ converging to $(A,u)\in \mathcal {C}_m$ we consider a converging subsequence of the Radon measures $\mu _k$ associated to the surface energy and $(A_k,u_k)$, and we estimate from below the Radon-Nikodym derivative of their limit denoted by $\mu _0$ with respect to the Hausdorff measure restricted to the 5 portions of $\partial A$ that appear in the definition of the surface anisotropy $\psi$ in (1.1). We overcome the fact that in general $\mu _0$ is not a non-negative measure due to the presence of the contact term in the energy with $\beta$, by adding to $\mu _k$ and $\mu _0$ the positive measure

$$\begin{aligned} \mu _\Sigma (B) = \int _{B\cap \Sigma } \varphi (x,\nu _\Sigma (x))\text {d}\mathcal {H}^1 \end{aligned}$$

defined for every Borel set $B\subset \mathbb {R}^2$ and using (1.2). The estimates for the Radon-Nikodym derivative related to the free boundary $\Omega \cap \partial ^*A$ and the contact region $(\Sigma \cap \partial ^*A){\setminus } J_{u_k}$ follow from [1, Lemma 3.8]. For the estimates related to exterior filaments and interior cracks we first separately reduce to the case of flat filaments and cracks, and then we adapt some arguments from [36]. Extra care is needed to treat the exterior filament lying on $\Sigma$ to which we refer as wetting layer in analogy to the thin-film setting. The estimate related to the delamination region on $\Sigma$ follows by blow-up under condition (4.2) that ensures that the delamination regions between the limiting free crystal *A* and the substrate $S$ can be originated from delamination regions between $A_k$ and $S$ and from portions of free boundaries $\partial ^*A_k$ or interior cracks collapsing on $\Sigma$, as well as from accumulation of interior cracks starting from $(\Sigma \cap \partial ^*A_k){\setminus } J_{u_k}$.

A challenging point is to prove that condition (4.2) is satisfied by $(A_k,u_k)$. In order to do this, in Theorem 2.8 we first extend the displacements $u_k$ to the set $\Omega {\setminus } (A_k\cup S)$ using Lemma 4.8. The extension of the $u_k$ is performed without creating extra jump at the interface on the exposed surface of the substrate, i.e., the jump set of the extensions is approximately $J_{u_k}\cup (\Omega \cap \partial A_k)$. We point out that as a consequence we obtain also in Proposition 4.9 the lower semicontinuity, with respect to the topology $\tau _\mathcal {C}'$, of a version of our energy without exterior filaments (but with wetting layer) extending the lower semicontinuity results of [22, 30, 31].

**Fig. 2:**
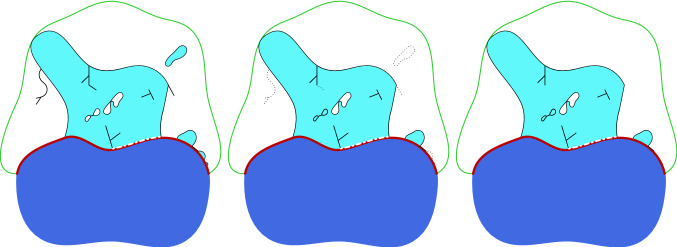
We pass from the set *A* represented on the left to the set $A''$ on the right by eliminating the external filaments, removing sufficiently small connected components of *A* and filling in sufficiently small holes

Finally, we prove (1.3), which entails the existence of a minimizing sequence $(A_m,u_m)\in \mathcal {C}_m$ for the minimum problem of $\mathcal {F}$ in $\mathcal {C}$. This is obtained by considering a minimizing sequence $(A_{\varepsilon },u_{\varepsilon })\in \mathcal {C}$ for $\mathcal {F}^{\lambda }$, and then by modifying it into a new minimizing sequence $(E_{\varepsilon ,\lambda },v_{\varepsilon ,\lambda })\in \mathcal {C}_m$ such that $\mathcal {F}^{\lambda }(A_{\varepsilon },u_{\varepsilon })+\delta _{\varepsilon }\geqq \mathcal {F}^{\lambda }(E_{\varepsilon ,\lambda },v_{\varepsilon ,\lambda })$ for some $\delta _{\varepsilon }\rightarrow 0$ as $\varepsilon \rightarrow 0$. The construction of $(E_{\varepsilon ,\lambda },v_{\varepsilon ,\lambda })\in \mathcal {C}_m$ requires 2 steps. In the first step we eliminate the external filaments, we remove sufficiently small connected components of $A_{\varepsilon }$, and we fill in sufficiently small holes till we reach a finite number of connected components with a finite number of holes (see Fig. 2). In the second step we redefine the deformations in the free crystal by employing [14, Theorem 1.1] in order to obtain a deformation with jump set consisting of at most finitely many components, and such that the difference in the elastic energy and the length of the jump sets with respect to $u_{\varepsilon }$ remains small.

The paper is organized as follows: in Section 2 we introduce the model and the topology $\tau _{\mathcal {C}}$, we refer to various SDRI settings from the literature that are included in our analysis, and we state the main results. In Section 3 we prove sequential compactness for the free crystals with the bound *m* on the boundary components in Proposition 3.3 and for $\mathcal {C}_m$ in Theorem 2.7. In Section 4 we prove the lower semicontinuity of the energy (Theorem 2.8) by first considering only the surface energy $\mathcal {S}$ under the condition (4.2) (see Proposition 4.1), and we conclude the section by showing the lower semicontinuity of the energy without the external filament and wetting-layer terms with respect to the topology $\tau _{\mathcal {C}}'$ (see Proposition 4.9). In Section 5 we prove the existence results (Theorems 2.6 and 2.9) and property (1.3). The paper is concluded with an Appendix where results related to rectifiable sets and Kuratowski convergence are recalled for reader’s convenience.

## Mathematical Setting

We start by introducing some notation. Since our model is two-dimensional, unless otherwise stated, all sets we consider are subsets of $\mathbb {R}^2.$ We choose the standard basis $\{\mathbf{e_1}=(1,0), \mathbf{e_2}=(0,1)\}$ in $\mathbb {R}^2$ and denote the coordinates of $x\in \mathbb {R}^2$ with respect to this basis by $(x_1,x_2).$ We denote by $\mathrm {Int}(A)$ the interior of $A\subset \mathbb {R}^2.$ Given a Lebesgue measurable set *E*,  we denote by $\chi _E$ its characteristic function and by |*E*| its Lebesgue measure. The set

$$\begin{aligned} E^{(\alpha )}:=\Big \{x\in \mathbb {R}^2:\,\, \lim \limits _{r\rightarrow 0} \frac{|E\cap B_r(x)|}{|B_r(x)|}=\alpha \Big \}, \qquad \alpha \in [0,1], \end{aligned}$$

where $B_r(x)$ denotes the ball in $\mathbb {R}^2$ centered at *x* of radius $r>0,$ is called the set of points of density $\alpha$ of *E*. Clearly, $E^{(\alpha )}\subset \partial E$ for any $\alpha \in (0,1),$ where

$$\begin{aligned} \partial E:=\{x\in \mathbb {R}^2:\,\,B_r(x)\cap E \ne \emptyset \text { and } B_r(x)\setminus E \ne \emptyset \text { for any } r>0\} \end{aligned}$$

is the topological boundary. The set $E^{(1)}$ is the *Lebesgue set* of *E* and $|E^{(1)}\Delta E|=0.$ We denote by $\partial ^*E$ the *reduced* boundary of a finite perimeter set *E* [3, 37], i.e.,

2.1 $$\begin{aligned} \partial ^*E:=\Big \{x\in \mathbb {R}^2:\,\, \exists \nu _E(x):=-\,\lim \limits _{r\rightarrow 0} \frac{D\chi _E(B_r(x))}{|D\chi _E|(B_r(x))},\quad |\nu _E(x)|=1\Big \}. \end{aligned}$$

The vector $\nu _E(x)$ is called the *measure-theoretic* normal to $\partial E.$

The symbol $$\mathcal {H}^s,$$$$s\geqq 0,$$ stands for the *s*-dimensional Hausdorff measure. An $$\mathcal {H}^1$$-measurable set *K* with $$0<\mathcal {H}^1(K)<\infty $$ is called $$\mathcal {H}^1$$-*rectifiable* if $$\theta ^*(K,x)=\theta _*(K,x) =1$$ for $$\mathcal {H}^1$$-a.e. $$x\in K,$$ where

$$\begin{aligned} \theta ^*(K,x):=\limsup \limits _{r\rightarrow 0^+} \frac{\mathcal {H}^1(B_r(x)\cap K)}{2r}, \qquad \theta _*(K,x):=\liminf \limits _{r\rightarrow 0^+} \frac{\mathcal {H}^1(B_r(x)\cap K)}{2r}. \end{aligned}$$

By [29, Theorem 2.3] any $\mathcal {H}^1$-measurable set *K* with $0<\mathcal {H}^1(K)<\infty$ satisfies $\theta ^*(K,x)=1$ for $\mathcal {H}^1$-a.e. $x\in K.$

### Remark 2.1

If *E* is a finite perimeter set, then

- $\overline{\partial ^*E} =\partial E^{(1)}$ (see, e.g., [37, Theorem 4.4] and [46, Eq. 15.3]);
- $\partial ^*E \subseteq E^{(1/2)}$ and $\mathcal {H}^{1}(E^{(1/2)}{\setminus } \partial ^*E) =0$ (see, e.g., [3, Theorem 3.61] and [46, Theorem 16.2]);
- $P(E,B) = \mathcal {H}^{1}(B\cap \partial ^*E)= \mathcal {H}^{1}(B\cap E^{(1/2)})$ for any Borel set *B*.

The notation $\mathrm {dist}(\cdot ,E)$ stands for the distance function from the set $E\subset \mathbb {R}^2$ with the convention that $\mathrm {dist}(\cdot ,\emptyset )\equiv +\infty .$ Given a set $A\subset \mathbb {R}^2,$ we consider also signed distance function from $\partial A,$ negative inside, defined as

$$\begin{aligned} \mathrm {sdist}(x,\partial A):= {\left\{ \begin{array}{ll} \mathrm {dist}(x, A) &{}\hbox { if}\ x\in \mathbb {R}^2{\setminus } A,\\ -\mathrm {dist}(x,\mathbb {R}^2{\setminus } A) &{}\hbox { if}\ x\in A. \end{array}\right. } \end{aligned}$$

### Remark 2.2

The following assertions are equivalent:

- $\mathrm {sdist}(x,\partial E_k) \rightarrow \mathrm {sdist}(x,\partial E)$ locally uniformly in $\mathbb {R}^2;$
- $E_k\overset{\mathcal {K}}{\rightarrow }\overline{E}$ and $\mathbb {R}^2{\setminus } E_k\overset{\mathcal {K}}{\rightarrow }\mathbb {R}^2{\setminus } \mathrm {Int}(E),$ where $\mathcal {K}$–Kuratowski convergence of sets [18, Chapter 4].

Moreover, either assumption implies $\partial E_k \overset{\mathcal {K}}{\rightarrow } \partial E.$

### The Model

Given two open sets $\Omega \subset \mathbb {R}^2$ and $S\subset \mathbb {R}^2{\setminus }\Omega ,$ we define the family of admissible regions for the *free crystal* and the space of *admissible configurations* by

$$\begin{aligned} \mathcal {A}:=\{A\subset \overline{\Omega } :\,\,\partial A \text { is } \mathcal {H}^1-\text {rectifiable and } \mathcal {H}^1(\partial A)<\infty \} \end{aligned}$$

and

$$\begin{aligned} \begin{aligned} \mathcal {C}:=\big \{(A,u):\,\,&A\in \mathcal {A},\\&u\in GSBD^2(\mathrm{Int}{(A\cup S\cup \Sigma )};\mathbb {R}^2) \cap H_\mathrm {loc}^1(\mathrm {Int}(A)\cup S;\mathbb {R}^2)\big \}, \end{aligned} \end{aligned}$$

respectively, where $\Sigma :=\partial S\cap \partial \Omega$ and $GSBD^2(E,\mathbb {R}^2)$ is the collection of all generalized special functions of bounded deformation [15, 19]. Given a displacement field $u\in GSBD^2(\mathrm{Int}{(A\cup S\cup \Sigma )};\mathbb {R}^2)\cap H_\mathrm {loc}^1(\mathrm {Int}(A)\cup S;\mathbb {R}^2)$ we denote by $e(u(\cdot ))$ the density of $\mathbf{e}(u)=(Du+(Du)^T)/2$ with respect to Lebesgue measure $\mathcal {L}^2$ and by $J_u$ the jump set of *u*. Recall that $e(u)\in L^2(A\cup S)$ and $J_u$ is $\mathcal {H}^1$-rectifiable. Notice also that assumption $u\in H_\mathrm {loc}^1(\mathrm {Int}(A)\cup S;\mathbb {R}^2)$ implies $J_u\subset \Sigma \cap \overline{\partial ^* A}.$ We denote the boundary trace of a function $u:A\rightarrow \mathbb {R}^n$ by $\mathrm {tr}_A$ (if exists).

#### Remark 2.3

For any $$A\in \mathcal {A}:$$

- $\partial A = N\cup (\Omega \cap \partial ^*A) \cup (\partial \Omega \cap \partial A) \cup (\Omega \cap A^{(0)}\cap \partial A)\cup (\Omega \cap A^{(1)}\cap \partial A),$ where *N* is an $\mathcal {H}^1$-negligible set (see, e.g., [46, page 184]);
- $\Omega \cap \overline{\partial ^*A} = \Omega \cap \partial A^{(1)}$ (see Remark 2.1 (a) above);
- $\mathcal {H}^1(\overline{\partial ^*A}{\setminus } \partial ^*A) = 0$ (since $\overline{\partial ^*A}\subset \partial A$ is $\mathcal {H}^1$-rectifiable);
- up to a $\mathcal {H}^1$-negligible set, the trace of $A\in \mathcal {A}$ on $\partial \Omega$ is defined as $\partial \Omega \cap \partial ^*A$ (see, e.g., [1, Lemma 2.10]).

Unless otherwise stated, in what follows $\Omega$ and $S\subset \mathbb {R}^2{\setminus } \Omega$ are bounded Lipschitz open sets with finitely many connected components satisfying $\mathcal {H}^1(\partial S)+\mathcal {H}^1(\partial \Omega )<\infty$ and $\Sigma \subseteq \partial \Omega$ is a Lipschitz 1-manifold.

We introduce in $\mathcal {A}$ the following notion of convergence:

#### Definition 2.4

($\tau _{\mathcal {A}}$-*Convergence*) A sequence $\{A_k\}\subset \mathcal {A}$ is said to $\tau _{\mathcal {A}}$-converge to $A\subset \mathbb {R}^2$ and is written $A_k\overset{\tau _\mathcal {A}}{\rightarrow } A$ if

- $$\sup \limits _{k\geqq 1} \mathcal {H}^1(\partial A_k) < \infty ;$$
- $\mathrm {sdist}(\cdot ,\partial A_k)\rightarrow \mathrm {sdist}(\cdot ,\partial A)$ locally uniformly in $\mathbb {R}^2$ as $k\rightarrow \infty .$

We endow $\mathcal {C}$ with the following notion of convergence:

#### Definition 2.5

($\tau _{\mathcal {C}}$-*Convergence*) A sequence $\{(A_n,u_n)\}\subset \mathcal {C}$ is said to $\tau _\mathcal {C}$-converge to $(A,u)\in \mathcal {C}$, and is written $(A_n,u_n)\overset{\tau _{\mathcal {C}}}{\rightarrow } (A,u)$ if

- $$A_n\overset{\tau _{\mathcal {A}}}{\rightarrow } A,$$
- $$u_n\rightarrow u$$ a.e. in $$\mathrm {Int}(A)\cup S$$.^1^

The *energy* of admissible configurations is given by the functional $\mathcal {F}:\mathcal {C}\rightarrow [-\infty ,+\infty ],$

$$\begin{aligned} \mathcal {F}:=\mathcal {S}+ \mathcal {W}, \end{aligned}$$

where $\mathcal {S}$ and $\mathcal {W}$ are the surface and elastic energies of the configuration, respectively. The surface energy of $(A,u)\in \mathcal {C}$ is defined as

2.2 $$\begin{aligned} \mathcal {S}(A,u)&:= \int _{\Omega \cap \partial ^*A} \varphi (x,\nu _A(x))\text {d}\mathcal {H}^1(x) \nonumber \\&\quad +\int _{\Omega \cap (A^{(1)}\cup A^{(0)})\cap \partial A} \big (\varphi (x,\nu _A(x)) + \varphi (x,-\nu _A(x))\big )\text {d}\mathcal {H}^1(x)\nonumber \\&\quad + \int _{\Sigma \cap A^{(0)}\cap \partial A} \big (\varphi (x,\nu _\Sigma (x)) + \beta (x)\big )\text {d}\mathcal {H}^1(x) \nonumber \\&\quad + \int _{\Sigma \cap \partial ^*A{\setminus } J_u} \beta (x) \text {d}\mathcal {H}^1(x) + \int _{J_u} \varphi (x,-\nu _\Sigma (x))\,\text {d}\mathcal {H}^1(x), \end{aligned}$$

where $\varphi :{\overline{\Omega }}\times \mathbb {S}^1\rightarrow [0,+\infty )$ and $\beta :\Sigma \rightarrow \mathbb {R}$ are Borel functions denoting the *anisotropy* of crystal and the *relative adhesion* coefficient of the substrate, respectively, and $\nu _\Sigma :=\nu _S$. In the following we refer to the first term in (2.2) as the *free-boundary energy*, to the second as the *energy of internal cracks and external filaments*, to the third as the *wetting-layer energy*, to the fourth as the *contact energy*, and to the last as the *delamination energy*. In applications instead of $\varphi (x,\cdot )$ it is more convenient to use its positively one-homogeneous extension $|\xi |\varphi (x,\xi /|\xi |).$ With a slight abuse of notation we denote this extension also by $\varphi .$

The elastic energy of $(A,u)\in \mathcal {C}$ is defined as

$$\begin{aligned} {{\mathcal {W}}}(A,u):= \int _{A\cup S} W(x,e(u(x)) - E_0(x))\text {d}x, \end{aligned}$$

where the elastic density *W* is determined as the quadratic form

2.3 $$\begin{aligned} W(x,M): = \mathbb {C}(x)M:M, \end{aligned}$$

by the so-called *stress-tensor*, a measurable function $x\in \Omega \cup S\rightarrow \mathbb {C}(x),$ where $\mathbb {C}(x)$ is a non-negative fourth-order tensor in the Hilbert space $\mathbb {M}^{2\times 2}_{\mathrm{sym}}$ of all $2\times 2$-symmetric matrices with the natural inner product

$$\begin{aligned} M:N=\sum \limits _{i,j=1}^2 M_{ij}N_{ij} \end{aligned}$$

for $$ M=(M_{ij})_{1\leqq i,j\leqq 2}, N=(N_{ij})_{1\leqq i,j\leqq 2}\in \mathbb {M}^{2\times 2}_{\mathrm{sym}}. $$

The *mismatch strain*$x\in \Omega \cup S\mapsto E_0(x)\in \mathbb {M}^{2\times 2}_{\mathrm{sym}}$ is given by

$$\begin{aligned} E_0: = {\left\{ \begin{array}{ll} e(u_0) &{} \hbox { in}\ \Omega ,\\ 0 &{} \hbox { in}\ S, \end{array}\right. } \end{aligned}$$

for a fixed $$u_0\in H^1(\Omega )$$.

Given $m\geqq 1,$ let $\mathcal {A}_m$ be a collection of all subsets *A* of ${\overline{\Omega }}$ such that $\partial A$ has at most *m* connected components. Recall that since $\partial A$ is closed, it is $\mathcal {H}^1$-measurable. By Proposition A.2, $\partial A$ is $\mathcal {H}^1$-rectifiable so that $\mathcal {A}_m\subset \mathcal {A}.$ We call the set

$$\begin{aligned} \mathcal {C}_m:=\Big \{(A,u)\in \mathcal {C}:\,\,A\in \mathcal {A}_m \Big \} \end{aligned}$$

the set of constrained admissible configurations. We also consider a volume constraint with respect to $\mathtt {v}\in (0,|\Omega |],$ i.e.,

$$\begin{aligned} |A|=\mathtt {v}\end{aligned}$$

for every $$A\in \mathcal {A}.$$

### Applications

The model introduced in this paper includes the settings of various free boundary problems, some of which are outlined below.

- *Epitaxially-strained thin films* [10, 22, 23, 31, 35]: $$\Omega :=(a,b)\times (0,+\infty ),$$$$S:=(a,b)\times (-\infty ,0)$$ for some $$a<b,$$ free crystals in the subfamily
  $$\begin{aligned} \begin{aligned} \quad \mathcal {A}_{\mathrm{subgraph}}:=\{A\subset \Omega :\, \exists h\in BV(\Sigma ;[0,\infty )) \text { and l.s.c. such that } A=A_h \}\subset \mathcal {A}_1, \end{aligned} \end{aligned}$$
  where $A_h:=\{(x^1,x^2)\,:\, 0<x^2<h(x^1)\}$, and admissible configurations in the subspace
  $$\begin{aligned} \mathcal {C}_{\mathrm{subgraph}}:=\{(A,u):\, A\in \mathcal {A}_{\mathrm{subgraph}},\, u\in H_\mathrm {loc}^1(\mathrm{Int}{(A\cup S\cup \Sigma )};\mathbb {R}^2)\}\subset \mathcal {C}_1 \end{aligned}$$
  (see also [6, 38]). Notice that the container $\Omega$ is not bounded, however, we can reduce to the situation of bounded containers where we can apply Theorem 2.9 since every energy equibounded sequence in $\mathcal {A}_{\mathrm{subgraph}}$ is contained in an auxiliary bounded set (see also Remark 2.10).
- *Crystal cavities* [30, 34, 47, 49]: $\Omega \subset \mathbb {R}^2$ smooth set containing the origin, $S:=\mathbb {R}^2{\setminus }\Omega ,$ free crystals in the subfamily
  $$\begin{aligned} \mathcal {A}_{\mathrm{starshaped}}:=\{A\subset \Omega :\, \text {open, starshaped with respect to } (0,0), \text { and } \partial \Omega \subset \partial A \}\subset \mathcal {A}_1, \end{aligned}$$
  and the space of admissible configurations
  $$\begin{aligned} \mathcal {C}_{\mathrm{starshaped}}:=\{(A,u):\, \, A\in \mathcal {A}_{\mathrm{starshaped}},\, u\in H_\mathrm {loc}^1(\mathrm{Int}{(A\cup S\cup \Sigma )};\mathbb {R}^2)\}\subset \mathcal {C}_1. \end{aligned}$$
  See Remark 2.10.
- *Capillarity droplets, e.g.,* [11, 24, 26]: $$\Omega \subset \mathbb {R}^2$$ is a bounded open set (or a cylinder), $$\mathbb {C}=0,$$$$S= \emptyset $$, and admissible configurations in the collection
  $$\begin{aligned} \mathcal {C}_{\mathrm{capillary}}:=\{(A,0):\, \,A\in \mathcal {A}\}\subset \mathcal {C}. \end{aligned}$$
- *Griffith fracture model, e.g.,* [12, 13, 16, 17]: $$S=\Sigma =\emptyset $$$$E_0\equiv 0$$, and the space of configurations
  $$\begin{aligned} \mathcal {C}_{\mathrm{Griffith}}:=\{(\Omega {\setminus } K,u):\, K \text { closed, } \mathcal {H}^1-\text {rectifiable},\, u\in H_\mathrm {loc}^1(\Omega {\setminus } K;\mathbb {R}^2)\}\subset \mathcal {C}. \end{aligned}$$
- *Mumford-Shah model (without fidelity term), e.g.,* [3, 20, 45]: $$S=\Sigma =\emptyset ,$$$$E_0=0,$$$$\mathbb {C}$$ is such that the elastic energy $$\mathcal {W}$$ reduces to the Dirichlet energy, and the space of configurations
  $$\begin{aligned} \mathcal {C}_{\mathrm{Mumfard-Shah}}: = \{ (\Omega {\setminus } K,u)\in \mathcal {C}_{\mathrm{Griffith}}:\,\, u=(u_1,0)\}\subset \mathcal {C}. \end{aligned}$$
- *Boundary delaminations* [5, 27, 41, 43, 44, 50]: the setting of our model finds applications to describe debonding and edge delaminations in composites [50]. We notice that our perspective differs from [5, 43, 44] where reduced models for the horizontal interface between the film and the substrate are derived, since instead we focus on the 2-dimensional film and substrate vertical section.

### Main Results

In this subsection we state the main results of the paper. Let us formulate our main hypotheses:

1. $$\varphi \in C(\overline{\Omega }\times \mathbb {R}^2;[0,+\infty ))$$ and is a Finsler norm, i.e., there exist $$c_2\geqq c_1>0$$ such that for every $$x\in \overline{\Omega },$$$$\varphi (x,\cdot )$$ is a norm in $$\mathbb {R}^2$$ satisfying
  2.4 $$\begin{aligned} c_1|\xi | \leqq \varphi (x,\xi ) \leqq c_2|\xi |\quad \text { for any } x\in \overline{\Omega }\text { and } \xi \in \mathbb {R}^2; \end{aligned}$$
2. $$\beta \in L^\infty (\Sigma )$$ and satisfies
  2.5 $$\begin{aligned} -\varphi (x,\nu _\Sigma (x))\leqq \beta (x) \leqq \varphi (x,\nu _\Sigma (x))\qquad \text {for } \mathcal {H}^1-\text {a.e.}\ x\in \Sigma ; \end{aligned}$$
3. *W* is of the form (2.3) with $\mathbb {C}\in L^{\infty }(\Omega \cup S)$ such that
  2.6 $$\begin{aligned} \mathbb {C}(x)M:M \geqq 2c_3\,M:M\quad \hbox { for any}\ M\in \mathbb {M}^{2\times 2}_{\mathrm{sym}}\end{aligned}$$
  for some $$c_3>0.$$

#### Theorem 2.6

(Existence) Assume (H1)–(H3). Let either $$\mathtt {v}\in (0,|\Omega |)$$ or $$S=\emptyset .$$ Then for every $$m\geqq 1,$$$$\lambda >0$$ both the volume-constrained minimum problem 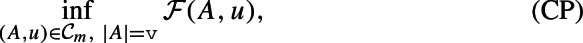 and the unconstrained minimum problem 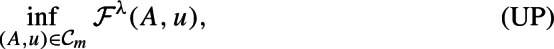 have a solution, where $\mathcal {F}^\lambda :\mathcal {C}_m\rightarrow \mathbb {R}$ is defined as

$$\begin{aligned} \mathcal {F}^\lambda (A,u):=\mathcal {F}(A,u) +\lambda \big ||A| - \mathtt {v}\big |. \end{aligned}$$

Furthermore, there exists $\lambda _0>0$ such that for every $\mathtt {v}\in (0,|\Omega |]$ and $\lambda >\lambda _0,$

2.7 $$\begin{aligned} \inf \limits _{(A,u)\in \mathcal {C},\,\,|A|=\mathtt {v}} \mathcal {F}(A,u) = \inf \limits _{(A,u)\in \mathcal {C}} \mathcal {F}^\lambda (A,u) = \lim \limits _{m\rightarrow \infty } \inf \limits _{(A,u)\in \mathcal {C}_m, \,\,|A|=\mathtt {v}} \mathcal {F}(A,u).\nonumber \\ \end{aligned}$$

We notice that for $\lambda >\lambda _0$ solutions of (CP) and (UP) coincide (see the proof of Theorem 2.6) for any $\mathtt {v}\in (0,|\Omega |]$ and $m\geqq 1$. Moreover, (2.7) shows that a minimizing sequence for $\mathcal {F}$ in $\mathcal {C}$ can be chosen among the sets whose boundary have finitely many connected components.

The proof of the existence part of Theorem 2.6 is given mainly by the following two results in which we show that $\mathcal {C}_m$ is $\tau _\mathcal {C}$-compact and $\mathcal {F}$ is $\tau _\mathcal {C}$-lower semicontinuous. Recall that an (infinitesimal) rigid displacement in $\mathbb {R}^n$ is an affine transformation $a(x)= Mx+b,$ where *M* is a skew-symmetric (i.e., $M^T = -M$) $n\times n$-matrix and $b\in \mathbb {R}^n.$ Given $B\in \mathcal {A}$ with $\mathrm {Int}(B) = \cup _{j} E_j,$ where $\{E_j\}$ are all connected components of $\mathrm {Int}(B),$ we say the function

$$\begin{aligned} a=\sum \limits _{j\geqq 1} (M_jx+b_j)\chi _{E_j}, \end{aligned}$$

a *piecewise rigid displacement* associated to *B*,  here $M_jx+b_j$ is a rigid displacement in $\mathbb {R}^2$.

#### Theorem 2.7

(Compactness of $\mathcal {C}_m$) Assume (H1)–(H3). Let either $\mathtt {v}\in (0,|\Omega |)$ or $S=\emptyset .$ Let $\{(A_k,u_k)\}\subset \mathcal {C}_m$ be such that

$$\begin{aligned} \sup \limits _{k\geqq 1} \mathcal {F}(A_k,u_k) < \infty \end{aligned}$$

and

2.8 $$\begin{aligned} |A_k|\leqq \mathtt {v}\end{aligned}$$

for every $k\geqq 1.$ Then there exist $(A,u)\in \mathcal {C}_m$ of finite energy, a subsequence $\{(A_{k_n},u_{k_n})\}$ and a sequence $\{(D_n,v_n)\}\subset \mathcal {C}_m$ with

$$\begin{aligned} v_n:=(u_{k_n}+a_n)\chi _{D_n\cap A_{k_n}} + u_0\chi _{D_n{\setminus } A_{k_n}} \end{aligned}$$

for some piecewise rigid displacements $$a_n$$ associated to $$D_n,$$ such that $$A_{k_n} \overset{\tau _\mathcal {A}}{\rightarrow } A,$$$$(D_n,v_n) \overset{\tau _\mathcal {C}}{\rightarrow } (A,u)$$, $$|D_n|=|A_{k_n}|,$$ and

2.9 $$\begin{aligned} \liminf \limits _{n\rightarrow \infty } \mathcal {F}(A_{k_n},u_{k_n}) \geqq \liminf \limits _{n\rightarrow \infty } \mathcal {F}(D_n,v_n). \end{aligned}$$

#### Theorem 2.8

(Lower semicontinuity of $\mathcal {F}$) Assume (H1)–(H3) and let $\{(A_k,u_k)\}\subset \mathcal {C}_m$ and $(A,u)\in \mathcal {C}_m$ be such that $(A_k,u_k)\overset{\tau _{\mathcal {C}}}{\rightarrow }(A,u).$ Then

$$\begin{aligned} \liminf \limits _{k\rightarrow \infty } \mathcal {F}(A_k,u_k) \geqq \mathcal {F}(A,u). \end{aligned}$$

As a byproduct of our methods we obtain the following existence result in a subspace of $\mathcal {C}_m$ with respect to a weaker topology previously used in [22, 30, 31] for thin films and crystal cavities.

#### Theorem 2.9

(Existence for weaker topology) Assume (H1)–(H3) and fix $m\geqq 1$ and $\mathtt {v}\in (0,|\Omega |].$ The functional $\mathcal {F}':\mathcal {C}\rightarrow \mathbb {R}$ defined as

$$\begin{aligned} \begin{aligned} \mathcal {F}'(A,u):=\mathcal {F}(A,u)&- 2\int _{\Omega \cap A^{(0)}\cap \partial A} \varphi (x,\nu _A)\text {d}\mathcal {H}^1\\&- \int _{\Sigma \cap A^{(0)} \cap \partial A} \big (\phi (x,\nu _A) + \beta \big ) \text {d}\mathcal {H}^1 -\int _\Sigma \beta \text {d}\mathcal {H}^1, \end{aligned} \end{aligned}$$

admits a minimizer (*A*, *u*) in every $\tau _\mathcal {C}'$-closed subset of

$$\begin{aligned} \mathcal {C}_m':=\big \{(A,u)\in \mathcal {C}:\,\, A \text { open, } |A| = \mathtt {v}, \text { and } A\cup \Sigma \in \mathcal {A}_m\big \}, \end{aligned}$$

where $\{(A_k,u_k)\}\subset \mathcal {C}$ converges to $(A,u)\in \mathcal {C}$ in $\tau _\mathcal {C}'$-sense if

- $$\sup \limits _{k\geqq 1}\,\,\mathcal {H}^1(\partial A_k)<\infty ,$$
- $$\mathbb {R}^2{\setminus } A_k \overset{\mathcal {K}}{\rightarrow } \mathbb {R}^2{\setminus } A,$$
- $$u_k\rightarrow u$$ a.e. in $$\mathrm {Int}(A)\cup S.$$

#### Remark 2.10

The sets $\mathcal {C}_{\mathrm{subgraph}}$ and $\mathcal {C}_{\mathrm{starshaped}}$ defined in Section 2.2 are $\tau _\mathcal {C}'$-closed in $\mathcal {C}_m'$ (see e.g., [31, Proposition 2.2]). In the thin-film setting, we define $\varphi$ and $\beta$ as $\varphi : = \gamma _f$ and

$$\begin{aligned} \beta :=-\,\max \big \{\min \{\gamma _f,\gamma _s - \gamma _{fs}\},-\gamma _f\big \}, \end{aligned}$$

where $$\gamma _f,$$$$\gamma _s,$$ and $$\gamma _{fs}$$ denote the surface tensions of the film-vapor, substrate-vapor, and film-substrate interfaces, respectively. The energy $$\mathcal {F}'$$ coincides (apart from the presence of delamination) with the thin-film energy in [22, 23] in the case $$\gamma _f,$$$$\gamma _s,$$$$\gamma _{fs}$$ are constants, $$\gamma _s - \gamma _{fs}\geqq 0,$$$$\gamma _s>0,$$ and $$\gamma _f>0.$$ Therefore, Theorem 2.9 extends the existence results in [22, 31] to all values of $$\gamma _s$$ and $$\gamma _s - \gamma _{fs}$$, as well as to anisotropic surface tensions and anisotropic elastic densities.

#### Remark 2.11

All the results contained in this subsection hold true with essentially the same proofs by replacing (H3) with the more general assumption

(H3’) $W:(\Omega \cup S)\times \mathbb {M}^{2\times 2}_{\mathrm{sym}}\rightarrow [0,\infty )$ is a function such that $M\mapsto W(x,M)$ is convex for any $x\in \Omega \cup S$ and
  $$\begin{aligned} c'|M|^p \leqq W(x,M) \leqq c''|M|^p +f(x) \end{aligned}$$
  for some $$p\geqq 2,$$$$c''\geqq c'>0$$ and $$f\in L^1(\Omega \cup S).$$

## Compactness

In this section we prove Theorem 2.7. Convergence of sets with respect to the signed distance functions has the following compactness property.

### Proposition 3.1

(Blaschke-type selection principle) For every sequence $\{A_k\}$ of subsets $\mathbb {R}^2$ there exist a subsequence $\{A_{k_l}\}$ and $A\subset \mathbb {R}^2$ such that $\mathrm {sdist}(\cdot ,\partial A_{k_l})\rightarrow \mathrm {sdist}(\cdot ,\partial A)$ locally uniformly in $\mathbb {R}^2$ as $l\rightarrow \infty .$

### Proof

Without loss of generality we suppose $A_k\notin \{\mathbb {R}^2,\emptyset \}.$ By the Blaschke selection principle [3, Theorem 6.1], there exists a not relabelled subsequence $\{A_k\}$ and a closed set $K\subset \mathbb {R}^2$ such that $\partial A_k$ converges to *K* in the Kuratowski sense as $k\rightarrow \infty .$ Notice that by Proposition A.1,

3.1 $$\begin{aligned} |\mathrm {sdist}(\cdot ,\partial A_k)|\rightarrow \mathrm {dist}(\cdot , K) \end{aligned}$$

locally uniformly as $k\rightarrow \infty$ since $|\mathrm {sdist}(\cdot ,\partial A_k)| = \mathrm {dist}(\cdot ,\partial A_k).$ As $\mathrm {sdist}(\cdot ,\partial A_k)$ is 1-Lipschitz, by the Arzela-Ascoli Theorem, passing to a further not relabelled subsequence one can find $f:\mathbb {R}^2\rightarrow [-\infty ,+\infty ]$ such that

$$\begin{aligned} \mathrm {sdist}(\cdot ,\partial A_k) \rightarrow f \end{aligned}$$

locally uniformly in $\mathbb {R}^2$ as $k\rightarrow \infty .$ By (3.1), $|f(\cdot )|= \mathrm {dist}(\cdot ,K).$ Recall that *K* may have nonempty interior. Fix a countable set $Q\subset \mathrm {Int}(K)$ dense in $\mathrm {Int}(K),$ and define

$$\begin{aligned} A:=\{f< 0\}\cup (\mathrm {Int}(\overline{\{f>0\}})\cap \partial K) \cup Q. \end{aligned}$$

By construction, $\mathrm {Int}(A) = \{f<0\}$, $\overline{A}=\{f\leqq 0\}\cup K$ and $\partial A=\{f=0\}=K.$

Finally we show that

$$\begin{aligned} f(x) = \mathrm {sdist}(x,\partial A). \end{aligned}$$

If $x\in A,$ by the definition of *A* and *K*,  $f(x)\leqq 0$ so that

$$\begin{aligned} f(x) = -\mathrm {dist}(x,K) = -\mathrm {dist}(x,\partial A) = -\mathrm {dist}(x, \mathbb {R}^2{\setminus } A). \end{aligned}$$

Analogously, if $x\notin A,$ then $f(x)\geqq 0$ and hence

$$\begin{aligned} f(x) = \mathrm {dist}(x,K) = \mathrm {dist}(x,\partial A) = \mathrm {dist}(x, A). \end{aligned}$$

$$\square $$

In general, the collection $\mathcal {A}$ is not closed under $\tau _\mathcal {A}$-convergence. Indeed, let $E:=\{x_k\}$ be a countable dense set in $B_1(0)$ and $E_k:=\{x_1,\ldots ,x_k\}\in \mathcal {A}.$ Then $\mathcal {H}^1(\partial E_k) = 0,$ and $E_k\overset{\tau _{\mathcal {A}}}{\rightarrow } E$ as $k\rightarrow \infty$, but $E\notin \mathcal {A}$ since $\partial E = \overline{B_1(0)}.$ However, $\mathcal {A}_m$ is closed with respect to the $\tau _{\mathcal {A}}$-convergence.

### Lemma 3.2

Let $A\subset \Omega$ and $\{A_k\}\subset \mathcal {A}_m$ be such that $A_k\overset{\tau _{\mathcal {A}}}{\rightarrow } A$. Then:

- $$A\in \mathcal {A}_m$$ and
  3.2 $$\begin{aligned} \mathcal {H}^1(\partial A)\leqq \liminf \limits _{k\rightarrow \infty } \mathcal {H}^1(\partial A_k); \end{aligned}$$
- $$A_k \rightarrow A$$ in $$L^1(\mathbb {R}^2)$$ as $$k\rightarrow \infty .$$

### Proof

(a) By Remark 2.2, $\partial A_k\overset{\mathcal {K}}{\rightarrow }\partial A$ as $k\rightarrow \infty .$ Thus, by [36, Theorem 2.1] $\partial A$ has at most *m*-connected components, and (3.2) holds.

(b) As $\partial A_k\overset{\mathcal {K}}{\rightarrow }\partial A,$ for any $x\in \mathrm {Int}(A)$ resp. $x\in \mathbb {R}^2{\setminus }\overline{A},$ there exists $k_x>0$ such that $x\in A_k$ resp. $x\in \mathbb {R}^2{\setminus } \overline{A_k}$ for all $k>k_x.$ Finally, by (3.2), $|\partial A| = 0,$ and therefore,

$$\begin{aligned} \chi _{A_k} \rightarrow \chi _A\qquad \text {a.e.}\ x\in \mathbb {R}^2. \end{aligned}$$

Now (b) follows from the uniform boundedness of $\{A_k\}$ and the Dominated Convergence Theorem. $\quad \square$

Furthermore, sequences $\{A_k\}\subset \mathcal {A}_m$ with equibounded boundary lengths are compact with respect to the $\tau _\mathcal {A}$-convergence.

### Proposition 3.3

(Compactness of $\mathcal {A}_m$) Suppose that $\{A_k\}\subset \mathcal {A}_m$ is such that

$$\begin{aligned} \sup \limits _{k\geqq 1} \mathcal {H}^1(\partial A_k) <\infty . \end{aligned}$$

Then there exists a subsequence $\{A_{k_l}\}$ and $A\in \mathcal {A}_m$ such that $\mathcal {H}^1(\partial A)<\infty$ and $\mathrm {sdist}(\cdot ,\partial A_{k_l})\rightarrow \mathrm {sdist}(\cdot ,\partial A)$ locally uniformly in $\mathbb {R}^2$ as $l\rightarrow \infty .$

### Proof

By Proposition 3.1 there exists a not relabelled subsequence $$\{A_k\}$$ and a set *A* such that $$\partial A_k\overset{\mathcal {K}}{\rightarrow } \partial A$$ and $$\mathrm {sdist}(\cdot ,\partial A_k)\rightarrow \mathrm {sdist}(\cdot ,\partial A)$$ locally uniformly in $$\mathbb {R}^2$$ as $$k\rightarrow \infty .$$ By Lemma 3.2, $$A\in \mathcal {A}_m$$ and $$\mathcal {H}^1(\partial A)<\infty .$$$$\quad \square $$

### Proposition 3.4

Let $\{A_k\}\subset \mathcal {A}_m$ be such that $A_k\overset{\tau _\mathcal {A}}{\rightarrow } A$ as $k\rightarrow \infty .$ Suppose that

$$\begin{aligned} \mathrm {Int}(A)=\bigcup \limits _{h\in I}E_h,\qquad F=\bigcup \limits _{i\in I_1} E_i\qquad \text {and} \qquad G=\bigcup \limits _{j\in I_2} E_j, \end{aligned}$$

where $$E_h$$ are disjoint connected components of $$\mathrm {Int}(A),$$$$I_1$$ and $$I_2$$ are disjoint finite subsets of *I*. Then there exist a subsequence $$\{A_{k_l}\}$$ and a sequence $$\{\gamma _l\}$$ of $$\mathcal {H}^1$$-rectifiable sets in $$\mathbb {R}^2$$ such that

- $$\gamma _l\subset \mathrm {Int}(A_{k_l})$$ and $$\lim \limits _{l\rightarrow \infty } \mathcal {H}^1(\gamma _l) =0;$$
- $\mathrm {sdist}(\cdot ,\partial (A_{k_l}{\setminus } \gamma _l))\rightarrow \mathrm {sdist}(\cdot ,\partial A)$ as $l\rightarrow \infty$ locally uniformly in $\mathbb {R}^2;$
- for any connected open sets $D'\subset \subset F$ and $D''\subset \subset G$ there exists $l'$ such that $D'$ and $D''$ belong to different connected components of $\mathrm {Int}(A_{k_l}{\setminus } \gamma _l)$ for any $l>l'.$

We postpone the proof until after the next lemma. Before this, we need to introduce some notation. Let $n_0>1$ be such that $E_h\cap \{\mathrm {dist}(\cdot ,\partial A) > \frac{1}{n}\}\ne \emptyset$ for every $h\in I_1\cup I_2$ and $n> n_0.$ Given $h\in I_1\cup I_2,$ let $\{E_h^n\}_{n> n_0}$ be an increasing sequence of connected open sets satisfying $E_h\cap \{\mathrm {dist}(\cdot ,\partial A) >\frac{1}{n}\} \subseteq E_h^n\subset \subset E_h$ and

3.3 $$\begin{aligned} E_h=\bigcup \limits _{n\geqq n_0} E_h^n. \end{aligned}$$

By the *sdist*-convergence and the finiteness of $I_1\cup I_2,$ for any $n\geqq n_0$ there exist $k_n^0>0$ such that $E_h^n\subset \subset \mathrm {Int}(A_k)$ for all $k>k_n^0$ and $h\in I_1 \cup I_2.$ Let

3.4 $$\begin{aligned} 2d_n:= \min \limits _{i\in I_1,\,j\in I_2}\{\mathrm {dist}(E_i^n,E_j^n),\mathrm {dist}(E_i^n,\partial A),\mathrm {dist}(E_j^n,\partial A)\}. \end{aligned}$$

Note that $$0<d_n<\frac{1}{2n}.$$

The idea of the proof of Proposition 3.4 is to “partition” the connecting components of $$\mathrm {Int}(A_k)$$ which in the limit break down into connected components $$\{E_h\}_{h\in I'}$$ of $$\mathrm {Int}(A)$$ such that $$I'\cap I_1\ne \emptyset $$ and $$I'\cap I_2\ne \emptyset ,$$ for example in the case of neckpinches. More precisely, we cut out at most *m*-circles from $$\mathrm {Int}(A_k)$$ such that for any $$n>n_0,$$ for all sufficiently large *k* (depending only on *n*), any curve $$\gamma \subset \mathrm {Int}(A_k)$$ connecting a point of $$E_i^n,$$$$i\in I_1,$$ to a point of $$E_j^n,$$$$j\in I_2,$$ intersects at least one of these circles. The following lemma consists in performing this argument for fixed $$i\in I_1$$ and $$j\in I_2$$:

### Lemma 3.5

Under the assumptions of Proposition 3.4, let $$i\in I_1,$$$$j\in I_2,$$ and $$n>n_0$$ be such that the set

3.5 $$\begin{aligned} Y=Y_{ij}^n&:=\Big \{k\in \mathbb {N}:\, \exists D_k\subset \subset \mathrm {Int}(A_k)\, \text {closed, connected,} \nonumber \\&\quad \text {and such that } D_k\cap E_i^{n},D_k\cap E_j^{n}\ne \emptyset \Big \} \end{aligned}$$

is infinite. Then, there exists $k_n^{ij}>k_n^0$ such that for any $k\in Y$ with $k>k_n^{ij}$ there exists a collection $\{B_{r_k^l}(z_k^l)\}_l$ of at most *m* balls contained in $A_k$ such that $r_k^l<d_n$ and any curve $\gamma \subset \subset \mathrm {Int}(A_k),$ connecting a point of $E_i^{n}$ to a point of $E_j^{n},$ intersects at least one of $B_{r_k^l}(z_k^l).$

### Proof

We divide the proof into four steps.

*Step 1:* for any $k\in Y,$ let $C_k\subset \subset \mathrm {Int}(A_k)$ be any closed connected set intersecting both $E_i^{n_0}$ and $E_j^{n_0}.$ Then

$$\begin{aligned} \lim \limits _{k\in Y,\,k\rightarrow \infty }\, \mathrm {dist}(C_k,\partial A_k) =0. \end{aligned}$$

By contradiction, assume that there exists $\epsilon >0$ such that

3.6 $$\begin{aligned} \mathrm {dist}(C_k,\partial A_k) \geqq \epsilon \end{aligned}$$

for infinitely many $k\in Y.$ By the Kuratowski-compactness of closed sets there exist a closed connected set *C* and a not relabelled subsequence $\{C_k\}_{k\in Y}$ satisfying (3.6) for all $k\in Y$ such that $C_k\overset{K}{\rightarrow } C$ as $k\rightarrow \infty .$ Since $A_k\overset{\tau _\mathcal {A}}{\rightarrow }A,$ in view of Remark 2.2$\partial A_k\overset{K}{\rightarrow }\partial A$ and $D\subset A.$ Let $x\in C$ and $y\in \partial A$ be such that $|x-y| = \mathrm {dist}(C,\partial A).$ Then by the definition of the Kuratowski convergence, there exist sequences $x_k\in C_k$ and $y_k\in \partial A_k$ such that $x_k\rightarrow x$ and $y_k\rightarrow y.$ Since $|x_k-y_k| \geqq \mathrm {dist}(C_k,\partial A_k)\geqq \epsilon ,$ it follows that

3.7 $$\begin{aligned} \mathrm {dist}(C,\partial A) = |x- y| =\lim \limits _{k\rightarrow \infty } |x_k-y_k|\geqq \epsilon . \end{aligned}$$

Thus, $C\subset \subset \mathrm {Int}(A).$ In particular, (3.7) implies that the non-empty connected open set $\{\mathrm {dist}(\cdot ,C)<\frac{\epsilon }{4}\}$ is compactly contained in $\mathrm {Int}(A)$ and intersects both $E_i^{n_0}$ and $E_j^{n}$ so that $E_i^{n}\cup \{\mathrm {dist}(\cdot ,C)<\frac{\epsilon }{4}\} \cup E_j^{n} \subset \mathrm {Int}(A)$ is connected. But this is a contradiction since $E_i^{n}$ and $E_j^{n}$ belong to different connected components of $\mathrm {Int}(A).$

*Step 2:* for every $k\in Y$ there exists a path-connected closed set $L_k\subset \subset \mathrm {Int}(A_k)$ intersecting both $E_i^{n}$ and $E_j^{n}$ such that

3.8 $$\begin{aligned} \mathrm {dist}(L_k,\partial A_k) =\delta _k:= \sup \,\, \mathrm {dist}(D,\partial A_k), \end{aligned}$$

where $\sup$ is taken over all closed connected sets $D\subset \subset \mathrm {Int}(A_k),$ intersecting both $E_i^{n_0}$ and $E_j^{n_0}$ (such sets exist by definition of *Y*). Moreover, there exists $k_n^1>0$ such that $L_k$ contains $E_i^{n}\cup E_j^{n}$ and $\delta _k<d_n$ for any $k>k_n^1.$

Indeed, in view of the Kuratowski-compactness of closed sets and from the Kuratowski-continuity of $\mathrm {dist}(\cdot ,\partial A_k),$ (3.8) has a maximizer $L_k'.$ Applying Step 1 with $A_k$ and $C_k=L_k',$ we get $\delta _k\rightarrow 0$ as $k\rightarrow \infty .$ Let $L_k$ be the connected component of $\{\mathrm {dist}(\cdot ,\partial A_k)\geqq \delta _k\}$ containing $L_k'.$ Since $E_i^{n}\cup E_j^{n} \subset \subset \mathrm {Int}(A),$ the *sdist*-convergence and Remark 2.2, $E_i^{n}\cup E_j^{n} \subset \subset \mathrm {Int}(A_k)$ for all large *k*. More precisely, by the definition (3.4) of $d_n,$ there exists ${\bar{k}}_n^1>0$ such that

3.9 $$\begin{aligned} \min \{\mathrm {dist}(E_i^n,\partial A_k),\mathrm {dist}(E_j^n,\partial A_k)\}\geqq d_n \end{aligned}$$

for all $k>{\bar{k}}_n^1.$ By construction, $\mathrm {dist}(L_k,\partial A_k)= \delta _k,$ and since $\delta _k\rightarrow 0,$ there exists $k_n^1>{\bar{k}}_n^1$ such that $\delta _k<d_n$ for any $k\geqq k_n^1.$ Note that by (3.9) for such *k* we have also $E_i^{n}\cup E_j^{n} \subset L_k.$

Let us show that $L_k$ is also path-connected. Indeed, given $x\in L_k,$ consider the ball $B_r(x)$ for small $r<\delta _k.$ Then $L_k\cap B_r(x)$ is path-connected, otherwise there would exist a curve in $B_r(x)$ with endpoints in $L_k$ containing a point $z\in B_r(x){\setminus } L_k$ such that $\mathrm {dist}(z,\partial A_k)>\delta _k$ contradicting to the definition of $L_k.$ Thus, $L_k$ is locally path-connected. Now the compactness and the connectedness of $L_k$ imply its path-connectedness.

*Step 3:* given $x\in E_i^{n_0}$ and $y\in E_j^{n_0},$ let $\gamma _k\subset L_k$ be a curve connecting *x* to *y*. Then for any $k>k_\epsilon ^0$ there exists $z_k\in \gamma _k{\setminus } \overline{E_i^{n_0} \cup E_j^{n_0}}$ such that any curve $\gamma \subset \subset \mathrm {Int}(A_k)$ homotopic in $\mathrm {Int}(A_k)$ to $\gamma _k$ (with same endpoints) intersects the ball $B_{\delta _k}(z_k).$

Indeed, otherwise slightly perturbing the curve $\gamma _k$ around the points of the compact set $\gamma ':=\{x\in \gamma _k:\, \mathrm {dist}(x,\partial A_k)=\delta _k\}$ we would get a new curve ${\widetilde{\gamma }}_k\subset \subset \mathrm {Int}(A_k)$ connecting *x* to *y* for which $\mathrm {dist}(x,\gamma _k)>\delta _k$ for all $x\in {\widetilde{\gamma }}_k.$ Now the compactness of ${\widetilde{\gamma }}_k$ implies $\mathrm {dist}({\widetilde{\gamma }}_k,\partial A_k)>\delta _k,$ which contradicts to the definition (3.8) of $L_k.$

*Step 4:* now we prove the lemma.

Applying Steps 1-3 with $A_k,$ we find an integer $k_n^1>k_n^0,$ a curve $\gamma _k^1$ connecting a point of $E_i^{n_0}$ to a point $E_j^{n_0}$ such that

3.10 $$\begin{aligned} \mathrm {dist}(\gamma _k^1,\partial A_k)=r_k^1=\sup \mathrm {dist}(D,\partial A_k) < d_n \end{aligned}$$

where *sup* is taken over all connected and closed $D\subset \subset \mathrm {Int}(A_k)$ intersecting both $E_i^{n_0}$ and $E_j^{n_0},$ and a ball $B_{r_k^1}(z_k^1)\subset A_k$ with $z_k^1\in \gamma _k^1$ such that any curve $\gamma \subset \subset \mathrm {Int}(A_k)$ homotopic to $\gamma _k^1$ intersects $B_{r_k^1}(z_k^1)$ for any $k\in Y$ with $k>k_n^1.$

For $k\in Y$ with $k>k_n^1$ set

$$\begin{aligned} A_k^1:=A_k{\setminus }(\mathrm {Int}(A_k)\cap \partial B_{r_k^1}(z_k^1)). \end{aligned}$$

Now consider the set $Y_1$ of all $k\in Y$ for which there exists a closed connected set $C_k\subset \subset \mathrm {Int}(A_k)$ intersecting both $E_i^{n_0}$ and $E_j^{n_0}.$ If $Y_1$ is finite, we set $k_n^{ij}:= \max \{\max Y_1, k_n^1\}$ and we are done.

Assume that $$Y_1$$ is infinite. Note that for any $$k\in Y_1,$$$$\partial B_{r_k^1}(z_k^1)$$ touches at least two different connected components of $$\partial A_k$$ and thus, $$A_k^1\in \mathcal {A}_{m-1}.$$ Applying Steps 1-3 with $$A_k^1$$ and $$Y_1,$$ we find an integer $$k_n^2>k_n^1,$$ a curve $$\gamma _k^2$$ connecting a point of $$E_i^{n_0}$$ to a point $$E_j^{n_0}$$ such that

$$\begin{aligned} \mathrm {dist}(\gamma _k^2,\partial A_k^1)=r_k^2=\sup \mathrm {dist}(D,\partial A_k^1) \end{aligned}$$

where *sup* is taken over all connected and closed $D\subset \subset \mathrm {Int}(A_k^1)$ intersecting both $E_i^{n_0}$ and $E_j^{n_0},$ and a ball $B_{r_k^2}(z_k^2)\subset A_k^1$ with $z_k^2\in \gamma _k^2$ such that any curve $\gamma \subset \subset \mathrm {Int}(A_k)$ homotopic to $\gamma _k^2$ intersects $B_{r_k^2}(z_k^2)$ for any $k\in Y_1$ with $k>k_n^2.$ By (3.10), $r_k^1\geqq r_k^2.$

For $k\in Y_1$ with $k>k_n^2$ set

$$\begin{aligned} A_k^2:=A_k{\setminus }(\mathrm {Int}(A_k)\cap (\partial B_{r_k^1}(z_k^1) \cup \partial B_{r_k^2}(z_k^2))) \end{aligned}$$

and consider the set $Y_2$ of all $k\in Y_1$ for which there exists a closed connected set $C_k\subset \subset \mathrm {Int}(A_k^2)$ intersecting both $E_i^{n_0}$ and $E_j^{n_0}.$ Note that $Y_2$ is finite, setting $k_n^{ij}:= \max \{\max Y_2, k_n^2\}$ and we are done. If $Y_2$ is infinite, then $A_k^2\in \mathcal {A}_{m-2},$ and we repeat the same procedure above. After at most *m* steps we obtain $k_n^{ij}>k_n^0$ such that for any $k>k_n^{ij}$ there is a collection $\{B_{r_k^l}(z_k^l)\}$ of at most *m* balls, which satisfy the assertion of the lemma. $\quad \square$

The assertions of Proposition 3.4 follow by applying Lemma 3.5 with all pairs $(i,j)\in I_1\times I_2.$

### Proof of Proposition 3.4

Given $$i\in I_1,$$$$j\in I_2$$ and $$n>n_0,$$ let $$Y_{ij}^n$$ be given by (3.5). If $$Y_{ij}^n$$ is infinite, let $$k_n^{ij}$$ be given by Lemma 3.5, otherwise set $$k_n^{ij}:= 1+\max Y_{ij}^n.$$ Let $$k_n:=1+\max \limits _{i,j}\,k_n^{ij}$$ and

$$\begin{aligned} \gamma _n^{ij}:= {\left\{ \begin{array}{ll} \mathrm {Int}(A_{k_n}) \cap \bigcup \limits _l \partial B_{r_{k_n}^l}^{ij}(z_{k_n}^l),&{} \hbox { if}\ k_n\in Y_{ij}^n,\\ \emptyset , &{} \hbox { if}\ k_n\notin Y_{ij}^n, \end{array}\right. } \end{aligned}$$

where $\{B_{r_k^l}^{ij}(z_k^l)\}$ is the collection of balls given by Lemma 3.5. Without loss of generality we assume that $\{k_n\}_n$ is strictly increasing and set

$$\begin{aligned} \gamma _n:=\bigcup \limits _{i,j} \gamma _n^{ij}. \end{aligned}$$

Being a union of at most $N_1N_2m$ circles, $\gamma _n$ is $\mathcal {H}^1$-rectifiable; here $N_i$ is the cardinality of $I_i.$ By Lemma 3.5,

3.11 $$\begin{aligned} \mathcal {H}^1(\gamma _n) \leqq \sum \limits _{i,j,l} \mathcal {H}^1(\partial B_{r_{k_n}^l}^{ij}(z_{k_n}^l)) \leqq 2\pi N_1N_2m\,d_n. \end{aligned}$$

Then $\lim \limits _{n\rightarrow \infty } \mathcal {H}^1(\gamma _n)=0$ and therefore, $\gamma _n$ converges in the Kuratowski sense to at most $N_1N_2m$ points on $\partial A.$

We claim that the sequences $$\{A_{k_n}\}$$ and $$\{\gamma _n\}$$ satisfy assertions (a)–(c). Indeed, by (3.11), $$\{\gamma _n\}$$ satisfy (a). Since $$\gamma _n$$ converges to at most $$N_1N_2m$$ points on $$\partial A$$ in the Kuratowski sense, (b) follows. To prove (c) , we take any connected open sets $$D'\subset \subset E$$ and $$D''\subset \subset F.$$ By connectedness and the definitions of $$E_h$$ and $$E_h^n,$$ there exist $$i\in I_1$$ and $$j\in I_2$$ and $${\bar{n}}>n_0$$ such that $$D'\subset \subset E_i^n$$ and $$D''\subset \subset E_j^n$$ for all $$n>\bar{n}.$$ By the construction of $$\gamma _n,$$ the sets $$E_i^n$$ and $$E_j^n$$ (and hence, $$D'$$ and $$D''$$) belong to different connected components of $$\mathrm {Int}(A_{k_n}){\setminus } \gamma _n$$ for all $$n>{\bar{n}}.$$$$\quad \square $$

By inductively applying Proposition 3.4 and by means of a diagonal argument we modify a sequence $$\{A_k\}$$$$\tau _\mathcal {A}$$-converging to a set *A* into a sequence $$\{B_k\}$$ with same $$\tau _\mathcal {A}$$-limit and whose (open) connected components “vanish” or “converge to the corresponding” connected components of *A*. This construction will be used in Step 1 of the proof of Theorem 2.7. We notice here that if $$S=\emptyset ,$$ then the sequence $$\{D_n\}$$ from Theorem 2.7 coincides with the sequence $$\{B_n\}.$$ Actually, if $$S=\emptyset ,$$ it would be enough to take $$D_n={\widetilde{B}}_n,$$ where $${\widetilde{B}}_n$$ is constructed in the Step 1 of the proof of the next proposition, since in this case we do not need properties (e) and (f) of the statement of the next proposition.

### Proposition 3.6

Let $A\in \mathcal {A}_m$ and $\{A_k\}\subset \mathcal {A}_m$ be such that $\mathrm {sdist}(\cdot ,\partial A_k)\rightarrow \mathrm {sdist}(\cdot ,\partial A)$ locally uniformly in $\mathbb {R}^2.$ Then there exist a subsequence $\{A_{k_l}\}$ and a sequence $\{B_l\}\subset \mathcal {A}_m$ such that

- $$\partial A_{k_l}\subset \partial B_l$$ and $$\lim \limits _{l\rightarrow \infty } \mathcal {H}^1(\partial B_l{\setminus } \partial A_{k_l})=0;$$
- $\mathrm {sdist}(\cdot ,\partial B_l)\rightarrow \mathrm {sdist}(\cdot ,\partial A)$ locally uniformly in $\mathbb {R}^2;$
- if $\{E_i\}$ is the set of all connected components of $\mathrm {Int}(A),$ we can choose a subfamily $\{E_i^l\}$ of connected components of $\mathrm {Int}(B_l)$ such that for any $G\subset \subset E_i$ there exists $l_{i,G}>0$ with $G\subset \subset E_i^l$ for every $l>l_{i,G};$
- $$|B_l| = |A_{k_l}|$$ for every $$l\geqq 1;$$
- $$\begin{aligned} \lim \limits _{l \rightarrow \infty } \sup \limits _{x\in E_i^l{\setminus } E_i} \mathrm {dist}(x, E_i)=0 \end{aligned}$$
  and
  $$\begin{aligned} \lim \limits _{l \rightarrow \infty } \mathcal {H}^1(\partial \Omega \cap (\partial E_i^l{\setminus } \partial E_i)) =0. \end{aligned}$$
- the boundary of every connected component of $\mathrm {Int}(B_l){\setminus }\bigcup _i E_i^l$ intersects the boundary of at most one connected component of $S.$

### Proof

Given $N,n\geqq 1,$ we define the index set $I_n^N$ by

$$\begin{aligned} I_n^N:=\Big \{i>N:\,\, E_i\cap \{\mathrm {dist}(\cdot ,\partial A)>\frac{1}{n}\}\ne \emptyset \Big \}. \end{aligned}$$

We notice that $I_n^N$ is finite since *A* is bounded.

*Step 1: Construction of*$\{{\widetilde{B}}_l\}$*and*$\{A_{k_l}\}$*satisfying (a)–(d).* This is done by using Proposition 3.4 iteratively in $N\in \mathbb {N}$ and a diagonal argument.

*Substep 1: Base of iteration.* By Proposition 3.4 applied with $$\{A_k\}_{k\in Y^0}$$ with $$Y^0:=\mathbb {N},$$$$I_1=\{1\},$$ and $$I_2=I_n^1$$ inductively with respect to $$n\in \mathbb {N},$$ we find a decreasing sequence $$Y^0\supset Y^1\supset \ldots $$ of infinite subsets of $$\mathbb {N}$$ such that for the subsequence $$\{A_k\}_{k\in Y^n}$$ there exists a sequence $$\{\gamma _k^n\}_{k\in Y^n}$$ of $$\mathcal {H}^1$$-rectifiable sets such that for any $$n\geqq 1:$$

- $\gamma _k^n\subset \mathrm {Int}(A_k)$ for any $k\in Y^n$ and $\lim \limits _{k\in Y^n,\, k\rightarrow \infty } \mathcal {H}^1(\gamma _k^n) =0;$
- for any connected open sets $D\subset \subset E_1$ and $D'\subset \subset \cup _{j\in I_n^1} E_j$ there exists $k'>0$ such that *D* and $D'$ belong to different connected components of $A_k{\setminus } \gamma _k^n$ for any $k\in Y^n$ with $k>k';$
- $\mathrm {sdist}(\cdot ,\partial (A_k{\setminus } \gamma _k^n)) \rightarrow \mathrm {sdist}(\cdot ,\partial A)$ as $Y^n\ni k \rightarrow \infty$ locally uniformly in $\mathbb {R}^2.$

Then by a diagonal argument, we choose an increasing sequence $n\in \mathbb {N}\mapsto k_n^1\in Y^n$ such that

$$\begin{aligned} {\widetilde{B}}_{1,n}:= A_{k_n}{\setminus } \gamma _{k_n^1}^n,\qquad n\in \mathbb {N}\end{aligned}$$

satisfies

- $a_{1n}$: $\partial A_{k_n}\subset \partial {\widetilde{B}}_{1,n}$ and $\mathcal {H}^1(\partial {\widetilde{B}}_{1,n}{\setminus } \partial A_{k_n})=\mathcal {H}^1(\gamma _{k_n^1}^n)<2^{-n}$ for any $n\geqq 1;$
- $b_{1n}$: $\mathrm {sdist}(\cdot ,\partial {\widetilde{B}}_{1,n})\rightarrow \mathrm {sdist}(\cdot ,\partial A)$ as $n\rightarrow \infty$ locally uniformly in $\mathbb {R}^2;$
- $c_{1n}$: for any connected open set $D\subset \subset E_1$ there exist $n_D^1>1$ and a unique connected component denoted by $E_1^{1,n}$ of $\mathrm {Int}({\widetilde{B}}_{1,n})$ such that $D\subset \subset E_1^{1,n}$ for all $n>n_D^1.$

*Substep 2: Iterative argument.* Repeating Substep 1 and applying Proposition 3.4 inductively in $$N=1,2,\dots ,$$ with $$A_k:={\widetilde{B}}_{N,k},$$$$I_1:=\{1,\ldots ,N\}$$ and $$I_2:=I_n^N$$ for $$n\in \mathbb {N},$$ we obtain $$\{{\widetilde{B}}_{N+1,n}\}_n\subset \mathcal {A}_m$$ and and increasing sequence $$n\in \mathbb {N}\mapsto k_n^{N+1}$$ with $$\{k_n^{N}\}_n\supset \{k_n^{N+1}\}_n$$ such that for any $$N\geqq 1:$$

- $$a_{Nn}$$: $$\partial A_{k_n^N}\subset \partial {\widetilde{B}}_{N,n},$$$$\partial {\widetilde{B}}_{N,n}\subset \partial {\widetilde{B}}_{N+1,n}$$ and $$\mathcal {H}^1(\partial {\widetilde{B}}_{N+1,n}{\setminus } \partial {\widetilde{B}}_{N,n})<2^{-(N+1)n}$$ for any $$n\geqq 1;$$
- $b_{Nn}$: $\mathrm {sdist}(\cdot ,\partial {\widetilde{B}}_{N,n})\rightarrow \mathrm {sdist}(\cdot ,\partial A)$ as $n\rightarrow \infty$ locally uniformly in $\mathbb {R}^2;$
- $c_{Nn}$: for any connected open set $D\subset \subset E_i$ for some $i\in \{1,\ldots ,N\}$ there exist $n_D^i>1$ and a unique connected component denoted by $E_i^{N,n}$ of $\mathrm {Int}({\widetilde{B}}_{N,n})$ such that $D\subset \subset E_i^{N,n}$ for all $n>n_D^i.$

By condition $b_{Nn}$ in Substep 2 and by the uniform boundedness of $\{{\widetilde{B}}_{N,n}\},$ there exists an increasing sequence $N\in \mathbb {N}\mapsto n_N\in \mathbb {N}$ such that the sequence ${\widetilde{B}}_N:={\widetilde{B}}_{N,n_N}$ satisfies $\mathrm {sdist}(\cdot ,\partial {\widetilde{B}}_N)\rightarrow \mathrm {sdist}(\cdot ,\partial A)$ as $N\rightarrow \infty$ locally uniformly in $\mathbb {R}^2.$ By condition $a_{Nn_N}$ of Substep 2,

$$\begin{aligned} \partial A_{k_{n_N}^N}\subset \partial {\widetilde{B}}_{1,n_N}\subset \ldots \subset \partial {\widetilde{B}}_{N,n_N}=\partial {\widetilde{B}}_N \end{aligned}$$

and

$$\begin{aligned} \mathcal {H}^1(\partial {\widetilde{B}}_N{\setminus } \partial A_{k_{n_N}^N}) \leqq \sum \limits _{i=1}^N \mathcal {H}^1(\partial {\widetilde{B}}_{i,n_N}{\setminus } \partial {\widetilde{B}}_{i-1,n_N}) \leqq \sum \limits _{i=1}^N 2^{-in_N}<2^{1-n_N}, \end{aligned}$$

where $${\widetilde{B}}_{0,n_N}:=A_{k_{n_N}^N}.$$

Furthermore, given $i\in \mathbb {N},$ if $D\subset \subset E_i$ is any connected open set, then by condition $c_{Nn_N},$ there exists a unique connected component ${\widetilde{E}}_i^N:=E_i^{N,n_N}$ of $\mathrm {Int}({\widetilde{B}}_N)$ such that $D\subset \subset E_i^N$ for all sufficiently large *N* (depending only *D* and *i*). Moreover, it is clear that $|{\widetilde{B}}_N|=|A_{k_{n_N}^N}|$ for any *N*. Hence, the sequence $\{{\widetilde{B}}_N\}_N$ and the subsequence $\{A_{k_{n_N}^N}\}_N$ satisfy assertions (a)–(d).

*Step 2: Construction of*$\{{\widehat{B}}_l\}$*and*$\{A_{k_l}\}$*satisfying (a)–(e).* Notice that $\mathrm {Int}({\widetilde{B}}_N)\subset \mathrm {Int}(A_{k_{n_N}^N})$ and by ${\widetilde{B}}_N\overset{\tau _\mathcal {A}}{\rightarrow } A$ and Lemma 3.2 (b), $\lim \limits _{N\rightarrow \infty } |{\widetilde{B}}_N\Delta A|\rightarrow 0.$ In particular, for any *i*, 

3.12 $$\begin{aligned} \lim \limits _{N \rightarrow \infty } |{\widetilde{E}}_i^N\Delta E_i|=0. \end{aligned}$$

By the Area Formula applied with $\mathrm {dist}(\cdot , E_i)$ we have

$$\begin{aligned} |{\widetilde{E}}_i^N {\setminus } E_i| = \int _0^\infty \mathcal {H}^1\big (({\widetilde{E}}_i^N{\setminus } E_i)\cap \{\mathrm {dist}(\cdot ,E_i) =t\}\big )\mathrm{d}t \end{aligned}$$

for any *i*. From this, (3.12) and a diagonal argument, there exists a not relabelled subsequence $\{{\widetilde{B}}_N\}$ for which

$$\begin{aligned} \lim \limits _{N\rightarrow \infty } \mathcal {H}^1\big (({\widetilde{E}}_i^N{\setminus } E_i)\cap \{\mathrm {dist}(\cdot ,E_i) =t\}\big ) =0 \end{aligned}$$

for any *i* and a.e. $t>0.$ Thus, we can choose $t_s\searrow 0$ for which

$$\begin{aligned} \lim \limits _{N\rightarrow \infty } \mathcal {H}^1\big (({\widetilde{E}}_i^N{\setminus } E_i)\cap \{\mathrm {dist}(\cdot ,E_i) =t_s\}\big ) =0 \end{aligned}$$

for any $s\in \mathbb {N}$ and *i*,  and thus, by a diagonal argument we find a further subsequence $\{{\widetilde{B}}_{N_s}\}_{s\in \mathbb {N}}$ such that

3.13 $$\begin{aligned} \mathcal {H}^1\big (({\widetilde{E}}_i^{N_s} {\setminus } E_i)\cap \{\mathrm {dist}(\cdot ,E_i) =t_s\}\big )<2^{-is} \end{aligned}$$

for any *i* and *s*. Let $\zeta _i^s:=({\widetilde{E}}_i^{N_s}{\setminus } E_i)\cap \{\mathrm {dist}(\cdot ,E_i) =t_s\},$ and let

$$\begin{aligned} {\widehat{B}}_s:={\widetilde{B}}_{N_s}{\setminus } \zeta _s, \end{aligned}$$

where $\zeta _s:=\bigcup \limits _{i} \zeta _i^s.$ Note that $\zeta _s$ is $\mathcal {H}^1$-rectifiable and by (3.13), $\mathcal {H}^1(\zeta _s) \leqq 2^{1-s}.$ Denote by ${\widehat{E}}_i^s$ the connected component of ${\widehat{B}}_s$ satisfying ${\widehat{E}}_i^s\subset {\widetilde{E}}_i^{N_s}$ and ${\widehat{E}}_i^s\cap E_i \ne \emptyset .$ By construction, $\sup \limits _{x\in {\widehat{E}}_i^s{\setminus } E_i} \mathrm {dist}(x, E_i)\leqq t_s,$ thus,

3.14 $$\begin{aligned} \limsup \limits _{s \rightarrow \infty }\sup \limits _{x\in {\widehat{E}}_i^s{\setminus } E_i} \mathrm {dist}(x, E_i)=0, \end{aligned}$$

and since $\partial A_{k_{n_{N_s}}^{N_s}}\subset \partial {\widetilde{B}}_{N_s}\subset \partial {\widehat{B}}_s,$ and

$$\begin{aligned} \limsup \limits _{s\rightarrow \infty } \mathcal {H}^1(\partial {\widehat{B}}_s{\setminus } \partial A_{k_{n_{N_s}}^{N_s}}) \leqq \limsup \limits _{s\rightarrow \infty } \Big (\mathcal {H}^1(\zeta _s) +\mathcal {H}^1(\partial {\widetilde{B}}_{N_s}{\setminus } \partial A_{k_{n_{N_s}}^{N_s}})\Big )=0. \end{aligned}$$

Moreover, since $\partial \Omega \cap (\partial {\widehat{E}}_i^s{\setminus } \partial E_i)\subset \{0<\mathrm {dist}(\cdot ,E_i)<t_s\}$ for any *s* and *i*,  we have

3.15 $$\begin{aligned}&\limsup \limits _{s\rightarrow \infty } \mathcal {H}^1(\partial \Omega \cap (\partial {\widehat{E}}_i^s{\setminus } \partial E_i)) \nonumber \\&\quad \leqq \lim \limits _{s\rightarrow \infty } \mathcal {H}^1(\partial \Omega \cap \{0<\mathrm {dist}(\cdot ,E_i)<t_s\} ) =0 \end{aligned}$$

for any *i* since $t_s\searrow 0.$ If $D\subset \subset E_i,$ then $D\subset \subset {\widehat{E}}_i^s$ provided that *s* is large. This, and the relations $\mathbb {R}^2{\setminus } \overline{{\widetilde{B}}_{N_s}} =\mathbb {R}^2{\setminus } \overline{{\widehat{B}}_s}$ and $\mathrm {Int}({\widehat{B}}_s)\subset \mathrm {Int}({\widetilde{B}}_{N_s})$ imply the local uniform convergence of $\mathrm {sdist}(\cdot ,\partial {\widehat{B}}_s)$ to $\mathrm {sdist}(\cdot ,\partial A)$ in $\mathbb {R}^2.$ Thus, $\{{\widehat{B}}_s\}$ and $\{A_{k_{n_{N_s}}^{N_s}}\}$ satisfy (a)–(e).

*Step 3: Construction of*$\{B_l\}$*and*$\{A_{k_l}\}$*satisfying (a)–(f).* Consider $C_s:=\mathrm {Int}({\widehat{B}}_s){\setminus } \bigcup _i{\widehat{E}}_i^s.$ Since $|E_i^s\Delta E_i|\rightarrow 0$ and $|\mathrm {Int}({\widehat{B}}_s)\Delta \mathrm {Int}(A)|\rightarrow 0$ as $s\rightarrow \infty ,$ we have $|C_s|\rightarrow 0.$ Therefore, applying the Area Formula with $\mathrm {dist}(\cdot ,S),$ we have

$$\begin{aligned} |C_s| = \int _0^\infty \mathcal {H}^1(C_s\cap \{\mathrm {dist}(\cdot ,S)=t\})dt \end{aligned}$$

so that, passing to further not relabelled subsequence if necessary, we can choose $t_s'\in (0,d_0/4)$ such that $\lim \limits _{s\rightarrow \infty } \mathcal {H}^1(C_s\cap \{\mathrm {dist}(\cdot ,S)=t_s'\})=0,$ where $d_0$ is the minimal distance between connected components of $S.$ Now the sequence

$$\begin{aligned} B_s:={\widehat{B}}_s{\setminus } (C_s\cap \{\mathrm {dist}(\cdot ,S)=t_s'\}) \end{aligned}$$

and the subsequence $\{A_{k_{n_{N_s}}^{N_s}}\}$ satisfy all assertions of the proposition. $\quad \square$

### Proposition 3.7

Let $Q\subset \mathbb {R}^n$ be a connected open set and $\{u_k\}\subset H^1_\mathrm {loc}(Q;\mathbb {R}^n)$ be such that

3.16 $$\begin{aligned} \sup \limits _k \int _Q|e(u_k)|^2\text {d}x<+\infty . \end{aligned}$$

Then either $|u_k|\rightarrow \infty$ a.e. in $Q$ or there exist $u\in H_\mathrm {loc}^1(Q;\mathbb {R}^n)\cap GSBD^2(Q;\mathbb {R}^n)$ and a subsequence $\{u_{k_l}\}$ such that $u_{k_l} \rightharpoonup u$ in $H^1_\mathrm {loc}(Q;\mathbb {R}^n),$ and hence, $u_{k_l}\rightarrow u$ a.e. in $Q.$

### Proof

Indeed, suppose that there exists a ball $B_\epsilon \subset \subset Q,$ a measurable function ${\widetilde{u}}: B_\epsilon \rightarrow \mathbb {R}^n$ and a not relabelled subsequence $\{u_k\}$ such that $u_k\rightarrow {\widetilde{u}}$ a.e. in some subset *E* of $B_\epsilon$ with positive measure. Since $u_k\in H^1(B_\epsilon ;\mathbb {R}^n),$ by the Poincaré-Korn inequality, there exists a rigid displacement $a_k:\mathbb {R}^n\rightarrow \mathbb {R}^n$ such that

$$\begin{aligned} \Vert u_k + a_k\Vert _{H^1(B_\epsilon )}^2 \leqq C\int _{B_\epsilon } |e(u_k)|^2\text {d}x \end{aligned}$$

for some $C>1$ independent of *k*. In particular, by the Rellich–Kondrachov Theorem, there exists $v\in H^1(B_\epsilon ;\mathbb {R}^n)$ such that $u_k +a_k \rightharpoonup v$ in $H^1(B_\epsilon ;\mathbb {R}^n)$ (up to a subsequence) and a.e. in $B_\epsilon .$ Since $u_k\rightarrow {\widetilde{u}}$ a.e. in *E*,  $a_k\rightarrow v - {\widetilde{u}}$ a.e. in *E* as $k\rightarrow \infty .$ Thus, $v - {\widetilde{u}}$ is a restriction in *E* of some rigid displacement $a:\mathbb {R}^n\rightarrow \mathbb {R}^n.$ By linearity of rigid displacements, $a_k\rightarrow a$ pointwise in $\mathbb {R}^n.$ Therefore, $u_k \rightharpoonup v-a$ in $H^1(B_\epsilon ;\mathbb {R}^n),$ hence a.e. in $B_\epsilon .$ In view of (3.16), $\{u_k\}\subset GSBD^2(Q;\mathbb {R}^n)$ with $J_{u_k}=\emptyset .$ Hence, by [15, Theorem 1.1], there exist a further not relabelled subsequence $\{u_k\}$ for which the set

$$\begin{aligned} F:= \{x\in Q:\,\,|u_k(x)|\rightarrow \infty \} \end{aligned}$$

has a finite perimeter in $\Omega$ and $u\in GSBD^2(Q;\mathbb {R}^n)$ such that $u_k\rightarrow u$ a.e. in $Q{\setminus } F$ and

3.17 $$\begin{aligned} \mathcal {H}^{n-1}(J_u{\setminus } \partial ^*F) + \mathcal {H}^{n-1}(Q\cap \partial ^*F) \leqq \liminf \limits _{k\rightarrow \infty } \mathcal {H}^1(J_{u_k}) =0. \end{aligned}$$

Thus, $P(F,Q)=0,$ i.e., either $F=\emptyset$ or $F=Q.$ Since $u_k\rightarrow u=v-a$ a.e. in $B_\epsilon \subset Q,$ the case $F=Q$ is not possible. Thus, $F=\emptyset .$ By (3.17), $\mathcal {H}^1(J_u)=0.$

Now we show that $u_k\rightharpoonup u$ in $H^1_\mathrm {loc}(Q;\mathbb {R}^n)$ and $u\in H^1_\mathrm {loc}(Q;\mathbb {R}^n).$ Let $D_1\subset \subset D_2\subset \subset \ldots$ be an increasing sequence of connected Lipschitz open sets such that $D_1:=B_\epsilon$ and $Q=\cup _j D_j.$ Applying Poincaré-Korn inequality $D_j$ we find a rigid displacement $a_k^j$ such that

$$\begin{aligned} \Vert u_k + a_k^j\Vert _{H^1(D_j)}^2 \leqq c_j\int _{D_j} |e(u_k)|^2\text {d}x, \end{aligned}$$

where $$c_j$$ is independent on *k*. Then by the Rellich–Kondrachov Theorem, every subsequence $$\{u_{k_l}\}$$ admits further not relabelled subsequence such that $$u_{k_l} + a_{k_l}^j \rightharpoonup v$$ in $$H^1(D_j;\mathbb {R}^n)$$ and a.e. in $$D_j$$ for some $$v\in H^1(D_j;\mathbb {R}^n).$$ Since $$u_{k_l} \rightarrow u$$ a.e. in $$D_j,$$ it follows that $$a_{k_l}^j \rightarrow v-u$$ a.e. in $$D_j$$ and hence, $$v-u$$ is also a rigid displacement. Since a.e.-convergence of linear functions implies the local strong $$H^1$$-convergence, $$u_{k_l}\rightharpoonup u$$ in $$H^1(D_j;\mathbb {R}^n),$$ and thus, $$u\in H^1(D_j;\mathbb {R}^n).$$ Since the subsequence $$\{u_{k_l}\}$$ is arbitrary, $$u_k\rightharpoonup u$$ in $$H^1(D_j;\mathbb {R}^n).$$ By the choice of $$D_j,$$$$u_k\rightharpoonup u$$ in $$H^1_\mathrm {loc}(Q;\mathbb {R}^n)$$ and $$u\in H^1_\mathrm {loc}(Q;\mathbb {R}^n).$$$$\quad \square $$

The following corollary of Proposition 3.7 is used in the proof of Theorem 2.7:

### Corollary 3.8

Let $P,P_k\subset \mathbb {R}^n$ be connected bounded open sets such that for any $G\subset \subset P$ there exists $k_G$ such that $G\subset \subset P_k$ for all $k>k_G,$ and let $u_k\in H^1_\mathrm {loc}(P_k;\mathbb {R}^n)$ be such that

3.18 $$\begin{aligned} \sup \limits _k \int _{P_k} |e(u_k)|^2\text {d}x<\infty . \end{aligned}$$

Then there exist $u\in H^1_\mathrm {loc}(P;\mathbb {R}^n)\cap GSBD^2(P;\mathbb {R}^n),$ a subsequence $\{(P_{k_l},u_{k_l})\}$ and a sequence $\{ b_l^P \}$ of rigid displacements such that $u_{k_l} + b_l^P \rightarrow u$ a.e. in $P.$

### Proof

Let $B_\epsilon \subset \subset P$ be any ball. By assumption, $B_\epsilon \subset \subset P_k$ for all large *k*. By the Poincaré-Korn inequality, for all such *k* there exists a rigid displacement $b_k^\epsilon$ such that

$$\begin{aligned} \Vert u_k + b_k^\epsilon \Vert _{H^1(B_\epsilon )}^2 \leqq C_\epsilon \int _{B_\epsilon } |e(u_k)|^2\text {d}x. \end{aligned}$$

This, (3.18) and the Rellich–Kondrachov Theorem imply that there exist a not relabelled subsequence $$\{u_k+ b_k^\epsilon \}$$ and $$v\in H^1(B_\epsilon ;\mathbb {R}^n)$$ such that $$u_k+ b_k^\epsilon \rightharpoonup v$$ as $$k\rightarrow \infty $$ in $$H^1(B_\epsilon ;\mathbb {R}^n),$$ hence, a.e. in $$B_\epsilon .$$ Now applying Proposition 3.7 with an increasing sequence $$\{G_i\}$$ of connected open sets satisfying $$G_1=B_\epsilon ,$$$$G_i\subset \subset P$$ and $$P=\cup _i G_i$$ we find $$u\in H_\mathrm {loc}^1(P;\mathbb {R}^n)\cap GSBD^2(P;\mathbb {R}^n)$$ with $$u=v$$ in $$B_\epsilon $$ and a not relabelled subsequence $$\{u_k+ b_k^\epsilon \}$$ such that $$u_k+ b_k^\epsilon \rightarrow u$$ as $$k\rightarrow \infty $$ a.e. in $$P.$$$$\quad \square $$

### Proposition 3.9

Assume (H1)–(H2) and let $$x_0\in \Sigma ,$$$$\delta \in (0,\frac{1}{2})$$ and $$r\in (0,1)$$ be such that $$\nu _0:=\nu _\Sigma (x_0)$$ exists. Then

3.19 $$\begin{aligned} |\varphi (y,\xi )- \varphi (x_0,\xi )|<\delta \end{aligned}$$

for any $$y\in U_{r,\nu _0}(x_0)$$ and $$\xi \in \mathbb {S}^1,$$$$U_{r,\nu _0}(x_0) \cap \Sigma $$ is a graph of a Lipschitz function over tangent line $$U_{r,\nu _0}(x_0) \cap T_{x_0}$$ in direction $$\nu _0$$ and

3.20 $$\begin{aligned} \int _{U_{r,\nu _0}(x_0) \cap \Sigma } |\beta (y) -\beta (x_0)|\text {d}\mathcal {H}^1<\delta \mathcal {H}^1(U_{r,\nu _0}(x_0)\cap \Sigma ). \end{aligned}$$

Let $$A\in \mathcal {A}_m$$ be such that $$x_0\in \Sigma \cap \partial ^*A,$$$$U_{r,\nu _0}(x_0)\cap \{\mathrm {dist}(\cdot ,T_{x_0})\geqq \delta r \}\subset \mathrm {Int}(A)\cup S,$$ and let $$\{(A_k,u_k)\}\subset \mathcal {C}_m$$ and $$u\in H_\mathrm {loc}^1(\mathrm {Int}(A);\mathbb {R}^2)$$ be such that $$A_k\overset{\tau _\mathcal {A}}{\rightarrow }A$$ and

$$\begin{aligned} \sup \limits _{k} \int _{U_{r,\nu _0}(x_0) \cap (A_k\cup S)} |e(u_k)|^2\text {d}x + \mathcal {H}^1(U_{r,\nu _0}(x_0) \cap \partial A_k) <\infty \end{aligned}$$

and $u_k\rightarrow u$ a.e. in $U_{r,\nu _0}(x_0) \cap \mathrm {Int}(A)$ and $|u_k|\rightarrow +\infty$ a.e. in $S\cap U_{r,\nu _0}(x_0).$ Then there exists $k_\delta >1$ for which

3.21 $$\begin{aligned}&\int _{U_{r,\nu _0}(x_0)\cap \Omega \cap \partial ^*A_k} \varphi (x,\nu _{A_k})\text {d}\mathcal {H}^1 +2\int _{U_{r,\nu _0}(x_0) \cap \Omega \cap (A_k^{(0)} \cup A_k^{(1)}) \cap \partial A_k} \varphi (x,\nu _{A_k})\text {d}\mathcal {H}^1\nonumber \\&\qquad + \int _{U_{r,\nu _0}(x_0) \cap \Sigma \cap A_k^{(0)}\cap \partial A_k} \big (\varphi (x,\nu _\Sigma ) + \beta \big )\text {d}\mathcal {H}^1\nonumber \\&\qquad + \int _{U_{r,\nu _0}(x_0)\cap \Sigma \cap \partial ^*A_k{\setminus } J_{u_k}} \beta \text {d}\mathcal {H}^1 + \int _{U_{r,\nu _0}(x_0) \cap J_{u_k}} \varphi (x,\nu _\Sigma )\,\text {d}\mathcal {H}^1 \nonumber \\&\quad \geqq \frac{1}{1+\frac{\delta }{c_2}} \int _{U_{r,\nu _0}(x_0) \cap \Sigma \cap \partial ^*A} \varphi (x,\nu _\Sigma ) \,\text {d}\mathcal {H}^1 - \delta \,\int _{U_{r,\nu _0}(x_0) \cap \Sigma }\varphi (x,\nu _\Sigma )\text {d}\mathcal {H}^1. \end{aligned}$$

for any $$k>k_\delta .$$

We postpone the proof unfil after the following lemma:

### Lemma 3.10

Let $$\phi $$ be a norm in $$\mathbb {R}^2,$$$$A\in \mathcal {A}_m$$ be such that $$0\in \Sigma \cap \partial ^*A,$$$$U_r \cap \{\mathrm {dist}(\cdot ,\{x_2=0\})\geqq \frac{r}{2} \}\subset \mathrm {Int}(A)\cup S,$$ and $$\{(A_k,u_k)\}\subset \mathcal {C}_m,$$ and $$u\in H^1_\mathrm {loc}(\mathrm {Int}(A);\mathbb {R}^2)$$ be such that

3.22 $$\begin{aligned} \sup \limits _{k} \int _{U_r \cap A_k} |e(u_k)|^2\text {d}x + \mathcal {H}^1(U_r \cap \partial A_k) <\infty \end{aligned}$$

and $A_k\overset{\tau _\mathcal {A}}{\rightarrow }A$ and $u_k\rightarrow u$ a.e. in $U_r \cap \mathrm {Int}(A)$ and $|u_k|\rightarrow +\infty$ a.e. in $S\cap U_r.$ Then for every $\epsilon >0$ there exists $k_\epsilon >0$ such that for any $k>k_\epsilon ,$

3.23 $$\begin{aligned}&\int _{U_r \cap \Omega \cap \partial ^*A_k} \phi (\nu _{A_k})\text {d}\mathcal {H}^1 +2\int _{U_r \cap \Omega \cap A_k^{(1)} \cap \partial A_k} \phi (\nu _{A_k})\text {d}\mathcal {H}^1 \nonumber \\&\quad \geqq 2\int _{U_r \cap \Sigma \cap (\partial ^* A_k {\setminus } J_{u_k})} \phi (\nu _\Sigma )\text {d}\mathcal {H}^1 -\epsilon . \end{aligned}$$

### Proof

Since $(A_k^{(1)}\cap \partial A_k)\cup J_{u_k}$ is $\mathcal {H}^1$-rectifiable, by [3, pp. 80] there exists at most countably many $C^1$-curves $\{\Gamma _i^k\}_{i\geqq 1}$ such that

$$\begin{aligned} \mathcal {H}^1\Big (((A_k^{(1)}\cap \partial A_k)\cup J_{u_k}){\setminus } \bigcup \limits _{i\geqq 1} \Gamma _i^k\Big ) =0. \end{aligned}$$

Selecting closed arcs inside curves if necessary, we suppose that $\Gamma _i^k\subset U_r$ and

$$\begin{aligned} \int _{U_r \cap A_k^{(1)}\cap \partial A_k} \phi (\nu _{A_k})\text {d}\mathcal {H}^1 + \int _{U_r \cap J_{u_k}} \phi (\nu _{A_k})\text {d}\mathcal {H}^1 +\epsilon > \sum \limits _{i\geqq 1} \int _{\Gamma _i^k} \phi (\nu _{\Gamma _i^k})\text {d}\mathcal {H}^1 \end{aligned}$$

for any *k*. Since each $$\Gamma _i^k$$ is $$C^1,$$ we can choose a Lipschitz open set $$V_i^k\subset U_r $$ such that $$\Gamma _i^k\subset \overline{V_i^k},$$$$|V_i^k|\leqq 2^{-i-1-k},$$

$$\begin{aligned} \int _{\partial V_i^k} \phi (\nu _{V_i^k})\text {d}\mathcal {H}^1 < 2\int _{\Gamma _i^k} \phi (\nu _{\Gamma _i^k})\text {d}\mathcal {H}^1 + \frac{\epsilon }{2^{i+1}} \end{aligned}$$

and $\mathrm {dist}_\mathcal {H}(\Gamma _i^k,\partial V_i^k)<2^{-k},$ where $\mathrm {dist}_\mathcal {H}$ is the Hausdorff distance (see e.g., (A.1) for the definition). Let $V_0^k:=U_r {\setminus } \overline{\mathrm {Int}(A_k)\cup S}$ be the “voids”. By the definition of $\{V_i^k\},$

3.24 $$\begin{aligned}&\int _{U_r \cap \Omega \cap \partial ^*A_k} \phi (\nu _{A_k})\text {d}\mathcal {H}^1 + \int _{U_r \cap (\Sigma {\setminus } \partial ^*A_k)} \phi (\nu _\Sigma ) \text {d}\mathcal {H}^1 + 2 \int _{U_r \cap J_{u_k}} \phi (\nu _{A_k})\text {d}\mathcal {H}^1 \nonumber \\&\quad + 2 \int _{U_r \cap \Omega \cap A_k^{(1)} \cap \partial A_k} \phi (\nu _{A_k})\text {d}\mathcal {H}^1 \geqq \sum \limits _{i\geqq 0} \int _{\partial V_i^k} \phi (\nu _{\partial V_i^k}) \text {d}\mathcal {H}^1 - \frac{\epsilon }{2}. \end{aligned}$$

In particular, by (3.22), $\sup \limits _k \sum \limits _{i\geqq 0} \mathcal {H}^1(\partial V_i^k) <\infty ,$ and hence, by [46, Proposition 2.6], there exists $\xi \in \mathbb {R}^2$ such that the set $\big \{x\in \bigcup _i\partial V_i:\,\,\mathrm {tr}_{U_r {\setminus } \cup V_i^k} (u)(x) = \xi \big \}$ is $\mathcal {H}^1$-negligible. Define

$$\begin{aligned} w_k:= u_k\chi _{U_r {\setminus } \bigcup _i V_i^k} + \xi \chi _{\bigcup _i V_i^k}. \end{aligned}$$

Then $$w_k\in GSBD^2(U_r ;\mathbb {R}^2),$$$$J_{w_k}=\bigcup _i\partial V_i^k$$ and by (3.22),

$$\begin{aligned} \sup \limits _k \int _{U_r } |e(w_k)|^2\text {d}x + \mathcal {H}^1(J_{w_k}) <\infty . \end{aligned}$$

Since $\sum \limits _{i\geqq 1}|V_i^k| \leqq 2^{-k},$ by assumption on $\{u_k\}$ and $\{A_k\},$

$$\begin{aligned} w_k\rightarrow {\left\{ \begin{array}{ll} u &{} \text {a.e.}\ \text {in } U_r\cap \mathrm {Int}(A), \\ \xi &{} \text {a.e.}\ \text {in } \Omega \cap U_r\cap {\setminus }\overline{A} \end{array}\right. } \end{aligned}$$

and $$|w_k|\rightarrow +\infty $$ a.e. in $$U_r\cap S.$$

We show that

3.25 $$\begin{aligned} 2\int _{U_r \cap \Sigma } \phi (\nu _\Sigma )\text {d}\mathcal {H}^1 \leqq \liminf \limits _{k\rightarrow \infty } \int _{J_{w_1}} \phi (\nu _{J_{w_1}})\text {d}\mathcal {H}^1. \end{aligned}$$

By assumption, $U_r \cap \Sigma \subset (-1,1)\times (-\epsilon ,\epsilon )$ and $U_r \cap \partial A \subset (-1,1)\times (-\epsilon ,\epsilon ),$ thus, by the convergence $A_k\overset{\tau _\mathcal {A}}{\rightarrow } A$ and Remark 2.2, $U_r \cap \partial A_k\subset (-1,1)\times (-\epsilon ,\epsilon )$ for all large *k*. In particular, for such *k*,  $J_{w_k}\subset (-1,1)\times (-\epsilon ,\epsilon ).$

Under the notation of [15], given $\xi \in \mathbb {S}^1$ let $\pi _\xi$ be the orthogonal projection onto the line $\Pi _\xi :=\{\eta \in \mathbb {R}^2:\,\,\xi \cdot \eta =0\},$ perpendicular to $\xi ;$ given a Borel set $F\subset \mathbb {R}^2$ and $y\in \Pi _\xi ,$ let $F_y^\xi :=\{t\in \mathbb {R}:\,\, y+t\xi \in F\}$ be the one-dimensional slice of *F*,  and given $u\in GSBD(U_r ;\mathbb {R}^2)$ and $y\in \Pi _\xi ,$ let ${\widehat{u}}_y^\xi (t) = u(y+t\xi )\cdot \xi$ be the one-dimensional slice of *u*. Since $w_h\rightarrow w$ a.e. in $U_r{\setminus }S,$ by [15, Eq. 3.23], for any $\epsilon >0$ and Borel set $F\subset U_r ,$

3.26 $$\begin{aligned} \mathcal {H}^0((F{\setminus } S)_y^\xi \cap J_{{\widehat{w}}_y^\xi }) + \mathcal {H}^0((U_r \cap \Sigma )_y^\xi ) \leqq \liminf \limits _{k\rightarrow \infty } \Big (\mathcal {H}^0(F_y^\xi \cap J_{({\widehat{w}}_k)_y^\xi }) + \epsilon f_y^\xi (w_k)\Big )\nonumber \\ \end{aligned}$$

for a.e. $\xi \in \mathbb {S}^1$ and a.e. $y\in \Pi _\xi ,$ where the integral of $f_y^\xi (w_k)$ over $\Pi _\xi$ is uniformly bounded independent on $\xi$ and *k* (see also (4.21) below).

Let

$$\begin{aligned} {\widehat{A}}:=\Big \{y\in \pi _\xi (U_r \cap \Sigma ):\,\, \liminf \limits _{k\rightarrow \infty } \mathcal {H}^0(J_{({\widehat{w}}_k)_y^\xi })=0\Big \}\subset \Pi _\xi . \end{aligned}$$

Then $F:=U_r \cap \pi _\xi ^{-1}({\widehat{A}})$ is Borel and, thus, integrating (3.26) over ${\widehat{A}}$ and using the definition of ${\widehat{A}}$ and Fatou’s Lemma we get

$$\begin{aligned} \mathcal {H}^1\big (\pi _\xi (U_r \cap \Sigma ) \cap {\widehat{A}}\big ) \leqq \liminf \limits _{k\rightarrow \infty } \epsilon \int _{{\widehat{A}}}f_y^\xi (v_k)\text {d}y\leqq M\epsilon \end{aligned}$$

for some $M>0$ independent of $\epsilon .$ Thus, letting $\epsilon \rightarrow 0$ we get $\mathcal {H}^1({\widehat{A}})=0.$ In particular,

3.27 $$\begin{aligned} \limsup \limits _{k\rightarrow \infty } \mathcal {H}^1\big (\pi _\xi (U_r \cap \Sigma ){\setminus } \pi _\xi (J_{w_k})\big )=0. \end{aligned}$$

Note that by construction, $J_{w_k}$ is a union of open sets, thus, for a.e. $y\in \pi _\xi (J_{w_k}),$ the line $\pi _\xi ^{-1}(y)$ passing through *y* and parallel to $\xi$ crosses $J_{w_k}$ at least at two points. Thus,

3.28 $$\begin{aligned} \mathcal {H}^0\big (J_{({\widehat{w}}_k)_y^\xi }\big )\geqq 2= 2\mathcal {H}^0\big ((U_r \cap \Sigma )_y^\xi \big ) \end{aligned}$$

for $\mathcal {H}^1$-a.e. $y\in \pi _\xi (J_{w_k})\cap \pi _\xi (U_r \cap \Sigma ),$ where $o(1)\rightarrow 0$ as $k\rightarrow \infty .$ Now we choose arbitrary pairwise disjoint open sets $F_1,F_2,\ldots \subset \subset U_r$ and repeating the same argument of Step 1 in the proof of Proposition 4.6 (by using (3.28) in place of (4.20) and using (3.27)) we obtain (3.25).

From (3.25) and (3.24) it follows that there exists $k_\epsilon >0$ such that

3.29 $$\begin{aligned}&\int _{U_r \cap \Omega \cap \partial ^*A_k} \phi (\nu _{A_k})\text {d}\mathcal {H}^1 + \int _{U_r \cap (\Sigma {\setminus } \partial ^*A_k)} \phi (\nu _\Sigma ) \text {d}\mathcal {H}^1 + 2 \int _{U_r \cap J_{u_k}} \phi (\nu _{A_k})\text {d}\mathcal {H}^1 \nonumber \\&\quad + 2 \int _{U_r \cap \Omega \cap A_k^{(1)} \cap \partial A_k} \phi (\nu _{A_k})\text {d}\mathcal {H}^1 \geqq 2\int _{U_r \cap \Sigma } \phi (\nu _\Sigma )\text {d}\mathcal {H}^1 - \epsilon \end{aligned}$$

for any $k>k_\epsilon .$ Now (3.23) follows from (3.29).   $\quad \square$

We anticipate here that in Lemma 4.7, below, we establish a similar result.

### Proof of Proposition 3.9

For simplicity, assume that $$x_0=0,$$$$\nu =\mathbf{e_2}$$ and $$\phi (\xi ) = \varphi (0,\xi ).$$ Denote the left-hand side of (3.21) by $$\alpha _k.$$ By (3.19) and (2.5),

3.30 $$\begin{aligned} \alpha _k\geqq&{\widehat{\alpha }}_k - 2\delta \mathcal {H}^1(U_r\cap \Omega \cap \partial A_k), \end{aligned}$$

where

$$\begin{aligned} {\widehat{\alpha }}_k:=&\int _{U_r \cap \Omega \cap \partial ^*A_k} \phi (\nu _{A_k})\text {d}\mathcal {H}^1 +2\int _{U_r \cap \Omega \cap (A_k^{(0)}\cup A_k^{(1)}) \cap \partial A_k} \phi (\nu _{A_k})\text {d}\mathcal {H}^1\nonumber \\&+ \int _{U_r \cap \Sigma \cap \partial ^*A_k{\setminus } J_{u_k}} \beta \text {d}\mathcal {H}^1+ \int _{U_r \cap J_{u_k}} \varphi (x,\nu _\Sigma )\,\text {d}\mathcal {H}^1. \end{aligned}$$

By Lemma 3.10 applied with $\phi$ and $\epsilon :=\delta \int _{U_r\cap \Sigma }\varphi (x,\nu _\Sigma )\text {d}\mathcal {H}^1$, there exists $k_\delta$ such that

$$\begin{aligned} {\widehat{\alpha }}_k\geqq 2\int _{U_r \cap \Sigma \cap (\partial ^* A_k {\setminus } J_{u_k})} \phi (\nu _\Sigma )\text {d}\mathcal {H}^1 + \int _{U_r \cap \Sigma \cap \partial ^*A_k{\setminus } J_{u_k}} \beta \text {d}\mathcal {H}^1+ \int _{U_r \cap J_{u_k}} \phi (\nu _\Sigma )\,\text {d}\mathcal {H}^1 -\epsilon \end{aligned}$$

for all $k>k_\delta .$ Then by (3.19)

$$\begin{aligned} \int _{U_r \cap \Sigma \cap (\partial ^* A_k {\setminus } J_{u_k})} \phi (\nu _\Sigma )\text {d}\mathcal {H}^1\geqq & {} \int _{U_r \cap \Sigma \cap (\partial ^* A_k {\setminus } J_{u_k})} \varphi (x,\nu _\Sigma )\text {d}\mathcal {H}^1\\&- \delta \mathcal {H}^1(U_r \cap \Sigma \cap (\partial ^* A_k {\setminus } J_{u_k})), \end{aligned}$$

and therefore,

$$\begin{aligned} {\widehat{\alpha }}_k\geqq & {} \int _{U_r \cap \Sigma \cap (\partial ^* A_k {\setminus } J_{u_k})} (2\varphi (x,\nu _\Sigma ) + \beta ) \text {d}\mathcal {H}^1 + \int _{U_r \cap J_{u_k}} \phi (\nu _\Sigma )\,\text {d}\mathcal {H}^1 \\&-\epsilon - \delta \mathcal {H}^1(U_r \cap \Sigma \cap (\partial ^* A_k {\setminus } J_{u_k})) \end{aligned}$$

Applying (2.5) in the first integral we get

$$\begin{aligned} {\widehat{\alpha }}_k\geqq \int _{U_r \cap \Sigma \cap \partial ^* A_k} \varphi (x,\nu _\Sigma ) \text {d}\mathcal {H}^1 -\epsilon - \delta \mathcal {H}^1(U_r \cap \Sigma \cap \partial ^* A_k) \end{aligned}$$

so that

3.31 $$\begin{aligned} \alpha _k \geqq \int _{U_r \cap \Sigma \cap \partial ^* A_k} \varphi (x,\nu _\Sigma ) \text {d}\mathcal {H}^1 - \epsilon - 2\delta \mathcal {H}^1(U_r \cap \partial A_k). \end{aligned}$$

By (2.4) and (2.5)

$$\begin{aligned} c_1 \mathcal {H}^1(U_r \cap \partial A_k) \leqq \alpha _k+ \int _{U_1\cap \Sigma }\varphi (x,\nu _\Sigma )\text {d}\mathcal {H}^1, \end{aligned}$$

and hence, (3.31) and the definition of $\epsilon$ imply

$$\begin{aligned} \Big (1+ \frac{\delta }{c_1}\Big )\,\alpha _k \geqq \int _{U_r \cap \Sigma \cap \partial ^* A_k} \varphi (x,\nu _\Sigma ) \text {d}\mathcal {H}^1 - \delta \Big (1+ \frac{\delta }{c_1}\Big )\int _{U_r\cap \Sigma }\varphi (x,\nu _\Sigma )\text {d}\mathcal {H}^1. \end{aligned}$$

and (3.21) follows. $$\quad \square $$

Finally we prove compactness of $\mathcal {C}_m.$

### Proof of Theorem 2.7

Let $R:=\sup \limits _k \mathcal {F}(A_k,u_k)$ and, by passing to a further not relabelled subsequence if necessary, we assume that

$$\begin{aligned} \liminf \limits _{k\rightarrow \infty } \mathcal {F}(A_k,u_k) = \lim \limits _{k\rightarrow \infty } \mathcal {F}(A_k,u_k). \end{aligned}$$

By (H1)–(H3) we have

$$\begin{aligned} \sup \limits _k \Big (c_1\mathcal {H}^1(\Omega \cap \partial A_k) + 2c_3\int _{A_k\cup S} |e(u_k)|^2\text {d}x\Big ) \leqq R + \int _\Sigma |\beta |\text {d}\mathcal {H}^1 \end{aligned}$$

and hence,

3.32 $$\begin{aligned} \mathcal {H}^1(\partial A_k) \leqq \mathcal {H}^1(\Omega \cap \partial A_k) + \mathcal {H}^1(\partial \Omega \cap \partial A_k) \leqq \frac{R +\int _\Sigma |\beta |\text {d}\mathcal {H}^1}{c_1} +\mathcal {H}^1(\partial \Omega )\nonumber \\ \end{aligned}$$

and

3.33 $$\begin{aligned} \int _{A_k\cup S} |e(u_k)|^2\text {d}x \leqq \frac{R +\int _\Sigma |\beta |\text {d}\mathcal {H}^1}{2c_3} \end{aligned}$$

for any $k\geqq 1.$ In view of (3.32) and Proposition 3.3, there exists $A\in \mathcal {A}_m$ with $\mathcal {H}^1(\partial A)<\infty$ and a not relabelled subsequence $\{A_k\}$ such that $\mathrm {sdist}(\cdot ,\partial A_k)\rightarrow \mathrm {sdist}(\cdot ,\partial A)$ locally uniformly in $\mathbb {R}^2.$ Now we construct the sequence $\{(B_n,v_n)\}$ in three steps. In the first step we apply Proposition 3.6 and Corollary 3.8 to obtain a (not relabelled) subsequence and to construct a sequence $\{B_k\}\subset \mathcal {A}_m$ with associated piecewise rigid displacements $\{a_k\}$ such that both $B_k\overset{\tau _\mathcal {A}}{\rightarrow } A$ and $u_k+a_k\rightarrow u$ a.e. in $\mathrm {Int}(A) \cup S$ for some $u\in H^1_\mathrm {loc}(\mathrm {Int}(A)\cup S,\mathbb {R}^2)\cap GSBD^2(\mathrm{Int}{(A\cup S\cup \Sigma )};\mathbb {R}^2).$ In the second step we take care of the fact that adding different rigid motions in $B_k$ and in $S$ can create extra jump at $\Sigma$ making difficult to satisfy (2.9). More precisely, by Proposition 3.9 we modify $\{B_k\}$ and $\{u_k\}$ so that the modified sequence $\{(B_k^\delta ,u_k^\delta )\}\subset \mathcal {C}_m$ satisfies (2.9) with some small error of order $\delta >0.$ Finally, in Step 3 we construct the sequence $\{(D_n,v_n)\}\subset \mathcal {C}_m$ by means of $\{(B_k^\delta ,u_k^\delta )\}$ and a diagonal argument.

*Step 1: Defining a first modification*$\{B_k\}$*of*$\{A_k\}$. By Proposition 3.6 there exist a not relabelled subsequence $\{A_k\}$ and a sequence $\{B_k\}\subset \mathcal {A}_m$ such that

1. $$\partial A_k\subset \partial B_k$$ and $$\lim \limits _{k\rightarrow \infty } \mathcal {H}^1(\partial B_k{\setminus } \partial A_k)=0;$$
2. $$B_k\overset{\tau _\mathcal {A}}{\rightarrow } A$$ as $$k\rightarrow \infty ;$$
3. if $\{E_i\}_{i\in I}$ is all connected components of $\mathrm {Int}(A),$ there exists connected components of $\mathrm {Int}(B_k)$ enumerated as $\{E_i^k\}_{i\in I}$ such that for any *i* and $G\subset \subset E_i$ one has $G\subset \subset E_i^k$ for all large *k* (depending only on *i* and *G*);
4. $$\sum \limits _i \mathcal {H}^1(\partial \Omega \cap (\partial E_i^k{\setminus } \partial E_i))\rightarrow 0$$ as $$k\rightarrow \infty ;$$
5. $$|B_k|=|A_k|$$ for all $$k\geqq 1;$$
6. the boundary of every connected component of $\mathrm {Int}(B_k){\setminus }\bigcup _i E_i^k$ intersects the boundary of at most one connected component of $S.$

Notice that by condition (a1),

$$\begin{aligned} \lim \limits _{k\rightarrow \infty }|\mathcal {S}(A_k,u_k) - \mathcal {S}(B_k,u_k)| =0 \end{aligned}$$

and

$$\begin{aligned} \mathcal {W}(A_k,u_k)= \mathcal {W}(B_k,u_k). \end{aligned}$$

Thus,

3.34 $$\begin{aligned} \lim \limits _{k\rightarrow \infty }|\mathcal {F}(A_k,u_k)- \mathcal {F}(B_k,u_k)|=0. \end{aligned}$$

Now we define the piecewise rigid displacements $a_k$ associated to $B_k.$ Let $\{S_j\}_{j\in Y}$ be the set of connected components of $S$ for some index set *Y*. We define the index sets $I_n\subset I$ and $Y_n\subset Y$ inductively on *n* in such a way that Corollary 3.8 holds with $P_k=E_i^k$ and $P=E_i$ and also with $P_k=P=S_j$ for every $i\in I_n$ and $j\in Y_n$ with the same rigid displacements $a_k^n$ independent of *i* and *j*.

More precisely, let $I_0:=Y_0:=\emptyset ,$ and given the sets $I_1,\ldots , I_{n-1}$ and $Y_1,\ldots ,Y_{n-1}$ for $n\geqq 1,$ we define $I_n$ and $Y_n$ as follows. By Corollary 3.8 applied with $P_k=P=S_{j_n}$ with $j_n$ the smallest element of $Y{\setminus } \bigcup \limits _{l=1}^{n-1} Y_l,$ we find a not relabelled subsequence $\{(B_k,u_k)\},$ a sequence $\{a_k^n\}$ of rigid displacements and $w_n\in H^1_\mathrm {loc}(S_{j_n};\mathbb {R}^2)$ such that $u_k+a_k^n\rightarrow w_n$ a.e. in $S_{j_n}.$ Let $I_n$ and $Y_n$ be the sets such that there exists a not relabelled subsequence $\{(B_k,u_k)\}$ such that the sequence $(u_k+a_k^n)\chi _{E_i^k}$ converges a.e. in $E_i$ for $i\in I_n$ and the sequence $(u_k+a_k^n)\chi _{S_j}$ converges a.e. in $S_j$ for $j\in Y_n.$ Recall that $j_n\in Y_n.$ Let

$$\begin{aligned} F_n^k:=\Big (\bigcup \limits _{i\in I_n} E_i^k\Big )\cup \Big (\bigcup \limits _{j\in Y_n} S_j\Big )\qquad \text {and}\qquad F_n:=\Big (\bigcup \limits _{i\in I_n} E_i\Big ) \cup \Big (\bigcup \limits _{j\in Y_n} S_j\Big ). \end{aligned}$$

By the definition of $I_n$ and $Y_n,$ and by diagonalization the sequence $(u_k+a_k^n)\chi _{F_n^k}$ converges as $k\rightarrow \infty$ a.e. in $F_n$ to some function in $H^1_\mathrm {loc}(F_n;\mathbb {R}^2),$ which we still denote by $w_n.$

Note that for large *n*,  $Y_n$ is empty since *Y* is finite by assumption. Notice also that by definition of $I_n$ and $Y_n,$ and Proposition 3.7 applied in connected open sets $P\subset \subset E_i\cup S_j,$ we have $|u_k+a_k^n|\rightarrow +\infty$ a.e in $E_i\cup S_j$ for every $i\in I{\setminus } I_n$ and $j\in Y{\setminus } Y_n,$

We now define the rigid displacements in $E_i^k$ for $i\in I{\setminus } \bigcup \limits _n I_n.$ By a diagonal argument and by Corollary 3.8 applied with $P_k=E_i^k$ and $P=E_i$ for any $i\in I{\setminus } \bigcup \limits _n I_n,$ we find a further not relabelled sequence $\{B_k,u_k\},$ sequence $\{{\widetilde{a}}_k^i\}$ of rigid displacements and $w^i\in H^1_\mathrm {loc}(E_i;\mathbb {R}^2)$ such that $(u_k+ {\widetilde{a}}_k^i)\chi _{E_i^k} \rightarrow w^i$ a.e. $E_i$ as $k\rightarrow \infty .$

Finally, we define rigid displacements in connected components $C_i^k$ of $B_k{\setminus }\bigcup _i E_i^k$ whose interior in the limit becomes empty, i.e., $C_i^k$ turns into an external filament. Recall that $|C_i^k|\rightarrow 0$ as $k\rightarrow \infty .$ If $\mathcal {H}^1(\partial C_i^k\cap \Sigma )=0,$ we define null-rigid displacement in $C_i^k.$ If $\mathcal {H}^1(\partial C_i^k\cap \Sigma )>0,$ then by condition (a6), $\partial C_i^k$ intersects the boundary of unique $S_{j_i},$ in which we have defined rigid displacement $a_k^{j_i}.$ In this case we define the same $a_k^{j_i}$ in $C_i^k$ so that $\bigcup _i \partial C_i^k\cap J_{u_k+a_k^{j_i}} \subset J_{u_k},$ that is, we do not create extra jump set.

Let

$$\begin{aligned} a_k:= \sum \limits _{n} a_k^n \sum \limits _{i\in I_n,j\in Y_n} \chi _{E_i^k\cup S_j}^{} + \sum \limits _{i\in I{\setminus } \cup _n I_n} {\widetilde{a}}_k^i\chi _{E_i^k}^{} + \sum _{i,\,\mathcal {H}^1(\partial C_i^k\cap \Sigma )>0} a_k^{j_i}\chi _{C_i^k} \end{aligned}$$

and

$$\begin{aligned} u:= \sum \limits _{n} w_n \sum \limits _{i\in I_n,j\in Y_n} \chi _{E_i\cup S_j}^{} + \sum \limits _{i\in I{\setminus } \cup _n I_n} w^i\chi _{E_i}^{}. \end{aligned}$$

By construction, $$a_k$$ is a piecewise rigid displacement associated to $$B_k,$$$$u\in H_\mathrm {loc}^1(\mathrm {Int}(A)\cup S;\mathbb {R}^2)$$ and $$u_k+ a_k\rightarrow u$$ a.e. in $$\mathrm {Int}(A)\cup S.$$ Note that $$e(u_k+a_k) = e(u_k).$$ Hence, by convergence $$A_k\overset{\tau _\mathcal {A}}{\rightarrow }A$$ and by (3.33), for any Lipschitz open set $$D\subset \subset \mathrm {Int}(A)\cup S,$$

$$\begin{aligned} \int _D |e(u_k+a_k)|^2\mathrm{d}x< \frac{R+\int _\Sigma |\beta |\text {d}\mathcal {H}^1}{2c_3} \end{aligned}$$

for all large *k* (depending only *D*). Since $u_k+a_k\rightarrow u$ a.e. in *D*,  by the Poincaré-Korn inequality, $e(u_k+a_k) \rightharpoonup e(u)$ weakly in $L^2(D;\mathbb {M}^{2\times 2}_{\mathrm{sym}}).$ Then by the convexity of $v\mapsto \int _D |e(v)|^2\text {d}x,$ we get

$$\begin{aligned} \int _D|e(u)|^2\text {d}x \leqq \liminf \limits _{k\rightarrow \infty } \int _D |e(u_k+a_k)|^2 \mathrm{d}x\leqq \frac{R+\int _\Sigma |\beta |\text {d}\mathcal {H}^1}{2c_3}. \end{aligned}$$

Hence, letting $D\nearrow \mathrm {Int}(A)\cup S$ we get $u\in GSBD^2(\mathrm{Int}{(A\cup S\cup \Sigma )};\mathbb {R}^2).$ Consequently, $(A,u)\in \mathcal {C}_m$ and $(B_k,u_k+a_k) \overset{\tau _\mathcal {C}}{\rightarrow }(A,u)$ as $k\rightarrow \infty .$

We observe that if $S=\emptyset ,$ the terms of the surface energy $\mathcal {S}(A_k,u_k)$ related to $\Sigma$ disappears, and hence, using $e(u_k +a_k) = e(u_k)$ and property (a1),

$$\begin{aligned} \mathcal {F}(A_k,u_k) =\mathcal {F}(B_k,u_k+a_k) + o(1), \end{aligned}$$

where $o(1)\rightarrow 0$ as $n\rightarrow \infty ,$ and so we can define $D_n=B_n.$

*Step 2: Further modification of*$\{B_k\}$. Without loss of generality we assume $\mathtt {v}<|\Omega |.$ It remains to control $J_{u_k+a_k}$ at $\Sigma$ since, as mentioned above, adding different rigid displacements to $u_k$ in connected components of the substrate and the free crystal whose closures intersect can result in a larger jump set $J_{u_k+a_k}$ than $J_{u_k}.$ Recall that by condition (a4) and (a6),

3.35 $$\begin{aligned} \lim \limits _{k\rightarrow \infty } \mathcal {H}^1(J_{u_k+a_k}{\setminus } \partial ^*A) =0. \end{aligned}$$

Hence, we need only to control $J_{u_k}\cap \partial ^*A.$ The idea here is to remove a “small” subset $R_k$ of $B_k$ containing almost all points $x\in \Sigma \cap \partial ^*A\cap (\partial ^*A_k{\setminus } J_{u_k})$ which in the limit becomes jump for *u*. In order to keep volume constraint, we will insert a square $U_k$ of volume $|R_k|$ in $\Omega {\setminus } A_k.$ This is possible since $|R_k|\rightarrow 0$ and $|A_k|\leqq \mathtt {v}$ for all *k*.

More precisely, we prove that for any $\delta \in (0,1/16),$ there exist $k_\delta >0$ and $(B_k^\delta ,u_k^\delta )\in \mathcal {C}_m$ with $|B_k|=|B_k^\delta |$ such that $(B_k^\delta ,u_k^\delta )$ and

3.36 $$\begin{aligned} \mathcal {F}(B_k,u_k) + 4\delta (1+c_2)\mathcal {H}^1(\Sigma ) + 4c_2 \delta > \mathcal {F}(B_k^\delta ,u_k+a_k) \end{aligned}$$

for any $$k>k_\delta .$$

We divide the proof into four steps.

*Substep 2.1.* By assumptions of the theorem, conditions (a2) and (a5) and Lemma 3.2 (b), $|A|\leqq \mathtt {v}.$ Hence, we can choose a square $U\subset \subset \Omega {\setminus }\overline{A}.$ By (a2) and the definition of $\tau _\mathcal {A}$-convergence, there is no loss of generality in assuming $U\subset \subset \Omega {\setminus } {\overline{B_k}}$ for any *k*. Let

3.37 $$\begin{aligned} \epsilon _0:=\sqrt{|U|}. \end{aligned}$$

Without loss of generality, we assume $\epsilon _0\in (0,\frac{1}{2}).$

First we observe that for any $\delta \in (0,1):$

1. since $$\Sigma $$ is Lipschitz, for $$\mathcal {H}^1$$-a.e. $$x\in \Sigma ,$$ there exist a unit normal $$\nu _\Sigma (x)$$ to $$\Sigma $$ and $$\overline{r_x}>0$$ such that for any $$r\in (0,\overline{r_x}),$$$$U_{r,\nu _\Sigma (x)}(x)\cap \Sigma $$ can be represented as a graph of a Lipschitz function over tangent line $$U_{r,\nu _\Sigma (x)}(x)\cap T_x$$ at *x* in the direction $$\nu _\Sigma (x);$$
2. since $\varphi$ is uniformly continuous, for any $x\in \overline{\Omega }$ there exists $\overline{r_x^\delta }>0$ such that for any $y\in U_{r_x^\delta ,\nu _\Sigma (x)}(x)$ and $\xi \in \mathbb {S}^1,$
  $$\begin{aligned} |\varphi (y,\xi ) - \varphi (x,\xi )|<\delta ; \end{aligned}$$
3. since $\mathcal {H}^1$-a.e. $x\in \Sigma$ is the Lebesgue point of $\beta ,$ there exists $\overline{r_x^\delta }>0$ such that for any $r\in (0,\overline{r_x^\delta }),$
  $$\begin{aligned} \int _{U_{r,\nu _\Sigma (x)}(x)\cap \Sigma } |\beta (y) - \beta (x)|\text {d}\mathcal {H}^1(y) <\delta \mathcal {H}^1(U_{r,\nu _\Sigma (x)}(x)\cap \Sigma ); \end{aligned}$$
4. for $\mathcal {H}^1$-a.e. $x\in \Sigma \cap \partial ^*A$ one has
  $$\begin{aligned} \theta ^*(\Sigma ,x) = \theta _*(\Sigma ,x)=\theta ^*(\Sigma \cap \partial ^*A,x) = \theta _*(\Sigma \cap \partial ^*A,x)=1, \end{aligned}$$
  thus, there exists $r_x>0$ such that for any $r\in (0,r_x),$
  $$\begin{aligned} \mathcal {H}^1(U_{r,\nu _\Sigma (x)}(x) \cap (\Sigma {\setminus } \partial ^*A )) <2\delta r; \end{aligned}$$
5. by Proposition A.4 applied with a connected component of $$\partial A,$$ for a.e. $$x\in \Sigma \cap \partial ^*A,$$$$ \overline{U_{1,\nu _\Sigma (x)}(x)}\cap \sigma _{\rho ,x}(\partial A) \overset{K}{\longrightarrow } \overline{U_{1,\nu _\Sigma (x)}(0)}\cap (T_x-x)$$ as $$\rho \rightarrow 0,$$ where $$\sigma _{\rho ,x}(y):=\frac{y-x}{\rho }$$ is the blow up map and $$T_x-x$$ is the straight line passing through the origin and parallel to the tangent line $$T_x$$ of $$\partial ^*A$$ (and of $$\Sigma $$) at *x*. Thus, there exists $$\overline{r_x^\delta }>0$$ such that for any $$r\in (0,\overline{r_x^\delta }),$$$$U_{r,\nu _\Sigma (x)}(x)\cap \{\mathrm {dist}(\cdot , T_x)>\delta r\}\subset \mathrm {Int}(A)\cup S.$$

By (3.35), there exists ${\bar{k}}_\delta >0$ such that

3.38 $$\begin{aligned} \mathcal {H}^1(J_{u_k+a_k}{\setminus } \partial ^*A)<\delta \end{aligned}$$

for any $$k>{\bar{k}}_\delta .$$

Fix $\delta \in (0,\frac{1}{16})$ and let $t_\delta >0$ be such that

3.39 $$\begin{aligned} |\{x\in \Omega :\,\,\mathrm {dist}(x,\Sigma )<t_\delta \}|< \delta ^2\epsilon _0^2, \end{aligned}$$

where $\epsilon _0$ is given in (3.37).

We now consider connected components $E_i$ and $S_j$ such that the associated rigid displacements are different. Let $I'$ be the set of all $i\in I$ such that $\mathcal {H}^1(\partial ^*E_i\cap \partial S)>0$ and $\liminf \limits _{k\rightarrow \infty } \mathcal {H}^1(\partial E_i^k\cap \partial S)>0,$ and there exists a connected component $S_j$ of $S$ such that $u_k+b_k^i\rightarrow u$ a.e. in $E_i$ and $|u_k+b_k^i|\rightarrow +\infty$ a.e. in $S_j$ for the associated sequence $\{b_k^i\}$ of rigid displacements in $E_i.$

Set

$$\begin{aligned} L:=\Sigma \cap \bigcup \limits _{i\in I'} \partial ^*E_i. \end{aligned}$$

Note that $\overline{L}\subset \Sigma$ and by (b1)–(b5), for a.e. $x\in \overline{L}$ for which $\nu _\Sigma (x)$ and $\nu _A(x)$ exist and $\nu _\Sigma (x)=\nu _A(x)$ there is

3.40 $$\begin{aligned} r_x:=r_x^\delta \in \left( 0,\frac{t_\delta }{8}\right) \end{aligned}$$

such that properties (b1)–(b5) holds with *x* and $r=r_x.$ Note that for any such *x* : 

1. since $B_k\overset{\tau _\mathcal {A}}{\rightarrow } A,$ by property (b5), there exists $\overline{k_x^\delta }>{\bar{k}}_\delta$ such that $U_{r,\nu _\Sigma (x)}(x)\cap \{\mathrm {dist}(\cdot , T_x)>\delta r\}\subset \mathrm {Int}(B_k)\cup S$ for any $k>\overline{k_x^\delta };$
2. by Proposition 3.9 applied with $u_k+a_k^i,$ there exists $\overline{k_x^\delta }>{\bar{k}}_\delta$ such that
  3.41 $$\begin{aligned}&\int _{U_{r,\nu _0}(x)\cap \Omega \cap \partial ^*A_k} \varphi (y,\nu _{A_k})\text {d}\mathcal {H}^1 +2\int _{U_{r,\nu _0}(x) \cap \Omega \cap (A_k^{(0)} \cup A_k^{(1)}) \cap \partial A_k} \varphi (y,\nu _{A_k})\text {d}\mathcal {H}^1\nonumber \\&\qquad + \int _{U_{r,\nu _0}(x) \cap \Sigma \cap A_k^{(0)}\cap \partial A_k} \big (\varphi (y,\nu _\Sigma ) + \beta \big )\text {d}\mathcal {H}^1\nonumber \\&\qquad + \int _{U_{r,\nu _0}(x)\cap \Sigma \cap \partial ^*A_k{\setminus } J_{u_k}} \beta \text {d}\mathcal {H}^1 + \int _{U_{r,\nu _0}(x) \cap J_{u_k}} \varphi (y,\nu _\Sigma )\,\text {d}\mathcal {H}^1 \nonumber \\&\quad \geqq \frac{1}{1+\frac{\delta }{c_2}} \int _{U_{r,\nu _0}(x) \cap \Sigma \cap \partial ^*A} \varphi (y,\nu _\Sigma ) \,\text {d}\mathcal {H}^1 - \delta \,\int _{U_{r,\nu _0}(x) \cap \Sigma }\varphi (y,\nu _\Sigma )\text {d}\mathcal {H}^1. \end{aligned}$$
  for any $k>\overline{k_x^\delta },$ where $r:=r_x$ and $\nu _0:=\nu _\Sigma (x).$

*Substep 2.2.* Let $x\in L$ be with properties (c1)–(c2) and let $U_r:=U_{r,\nu _\Sigma (x)}(x)$ and $Q_x\subset \Omega \cap$ be the open set whose boundary consists of $\Gamma _1:=U_r\cap \Sigma ,$ two segments $\Gamma _2,\Gamma _3\subset \partial U_r$ of length at most $2\delta r,$ parallel to $\nu _\Sigma (x),$ and the segment $\Gamma _4:= \Omega \cap U_r \cap \{\mathrm {dist}(\cdot ,T_x)=\delta \}$ of length 2*r*. Given $k>k_x^\delta$ let

$$\begin{aligned} {\widehat{B}}_k^\delta :=B_k{\setminus } Q_x)\cup (\overline{\Omega }\cap \partial U_r). \end{aligned}$$

Clearly, $({\widehat{B}}_k^\delta ,u_k+a_k)\in \mathcal {A}_m.$ We claim that

3.42 $$\begin{aligned} \mathcal {S}(B_k,u_k) \geqq \mathcal {S}({\widehat{B}}_k^\delta ,u_k) -4\delta (1+c_2)\mathcal {H}^1(U_r\cap \Sigma ). \end{aligned}$$

Indeed, without loss of generality, we assume that $x=0$ and $\nu _\Sigma (x)=\mathbf{e_2}.$ By the anisotropic minimality of segments,

3.43 $$\begin{aligned} \int _{\Gamma _1} \varphi (0,\nu _\Sigma ) \text {d}\mathcal {H}^1 + \int _{\Gamma _2\cup \Gamma _3} \varphi (0,\mathbf{e_2}) \text {d}\mathcal {H}^1 \geqq \int _{\Gamma _4} \varphi (0,\mathbf{e_2})\text {d}\mathcal {H}^1. \end{aligned}$$

Since $\mathcal {H}^1(\Gamma _1)\geqq 2r=\mathcal {H}^1(\Gamma _4)$ and $\mathcal {H}^1(\Gamma _2),\mathcal {H}^1(\Gamma _3)\leqq 2\delta r$ and, also by property (b2), we have

3.44 $$\begin{aligned} \int _{\Gamma _1} \varphi (0,\nu _\Sigma ) \text {d}\mathcal {H}^1 \leqq \int _{\Gamma _1} \varphi (y,\nu _\Sigma ) \text {d}\mathcal {H}^1 + \delta \mathcal {H}^1(\Gamma _1), \end{aligned}$$

and

3.45 $$\begin{aligned} \int _{\Gamma _2\cup \Gamma _3} \varphi (0,\mathbf{e_2}) \text {d}\mathcal {H}^1 \leqq 2\delta \varphi (0,\mathbf{e_2}) \mathcal {H}^1(\Gamma _1) \end{aligned}$$

and

3.46 $$\begin{aligned} \int _{\Gamma _4} \varphi (0,\mathbf{e_2})\text {d}\mathcal {H}^1 \geqq \int _{\Gamma _4} \varphi (y,\mathbf{e_2})\text {d}\mathcal {H}^1 - \delta \mathcal {H}^1(\Gamma _1), \end{aligned}$$

hence, using (2.4) in (3.45), from (3.43) and (3.44)–(3.46) we obtain

3.47 $$\begin{aligned} \int _{\Gamma _4} \varphi (y,\mathbf{e_2})\text {d}\mathcal {H}^1 \leqq \int _{\Gamma _1} \varphi (y,\nu _\Sigma ) \text {d}\mathcal {H}^1 + 2\delta (1+c_2) \mathcal {H}^1(\Gamma _1). \end{aligned}$$

Denoting by $\alpha _k$ the left-hand side of (3.41), by condition (a1) and the definition of ${\widehat{B}}_k^\delta ,$ we have

$$\begin{aligned} \mathcal {S}(B_k,u_k) - \mathcal {S}({\widehat{B}}_k^\delta ,u_k)&\geqq \alpha _k -2\int _{\partial B_k{\setminus }\partial A_k} \varphi (y,\nu _{B_k})\text {d}\mathcal {H}^1 \\&\quad - \int _{\Gamma _2\cup \Gamma _3\cup \Gamma _4} \phi (y,\nu _{B_k^\delta })\text {d}\mathcal {H}^1 - \int _{(B_k^\delta )^{(0)}\cap (\Gamma _2\cup \Gamma _3)} \phi (y,\nu _{B_k^\delta })\text {d}\mathcal {H}^1, \end{aligned}$$

thus, using $\Gamma _1=U_r\cap \Sigma ,$ from (3.41), (3.47), (3.45), (3.38) and (2.4) we obtain

$$\begin{aligned} \mathcal {S}(B_k,u_k) - \mathcal {S}({\widehat{B}}_k^\delta ,u_k)&\geqq \frac{1}{1+\frac{\delta }{c_2}} \int _{U_r\cap \Sigma \cap \partial ^*A} \varphi (y,\nu _\Sigma )\text {d}\mathcal {H}^1 - \int _{U_r\cap \Sigma } \varphi (y,\nu _\Sigma ) \text {d}\mathcal {H}^1\\&\quad - 3\delta (1+c_2) \mathcal {H}^1(U_r\cap \Sigma ). \end{aligned}$$

In the last inequality using $\frac{1}{1+\frac{\delta }{c_2}}\geqq 1 - \frac{\delta }{c_2}$ and inequality (2.4) once more we deduce

3.48 $$\begin{aligned} \mathcal {S}(B_k,u_k)\geqq & {} \mathcal {S}({\widehat{B}}_k^\delta ,u_k) - \int _{U_r\cap (\Sigma {\setminus } \partial ^*A)} \varphi (y,\nu _\Sigma )\text {d}\mathcal {H}^1 \nonumber \\&- \delta (4+3c_2) \mathcal {H}^1(U_r\cap \Sigma ). \end{aligned}$$

Now condition (b4) and (2.4) and the inequality $\mathcal {H}^1(\Gamma _1)\geqq 2r$ imply

$$\begin{aligned} \int _{U_r\cap (\Sigma {\setminus } \partial ^*A)} \varphi (y,\nu _\Sigma )\text {d}\mathcal {H}^1\leqq c_2\mathcal {H}^1(U_r\cap (\Sigma {\setminus } \partial ^*A)) \leqq 2\delta c_2 r \leqq \delta c_2 \mathcal {H}^1(U_r\cap \Sigma ). \end{aligned}$$

Inserting this in (3.48) we get (3.42).

*Substep 2.3.* Now we choose finitely many points $x_1\ldots ,x_N\in \overline{L}$ with corresponding $r_1,\ldots ,r_N$ satisfying (b1)–(b5) and (c1)–(c2) such that the squares $\{U_{r_j,\nu _\Sigma (x_j)}(x_j)\}_{j=1}^N$ are pairwise disjoint and

3.49 $$\begin{aligned} \mathcal {H}^1\Big (L {\setminus } \bigcup \limits _{j=1}^N U_{r_j,\nu _\Sigma (x_j)}(x_j)\Big ) <\delta . \end{aligned}$$

Recalling the definition of $k_\delta ^x$ in condition (c2) and the definition ${\bar{k}}_\delta$ in (3.38), let $k_\delta :=\max \{{\bar{k}}_\delta ,\overline{k_\delta ^{x_1}},\ldots ,\overline{k_\delta ^{x_N}}\}$ and let $Q_{x_j}\subset \Omega \cap U_{r_j,\nu _\Sigma (x_j)}(x_j)$ be as in Substep 2.2. Set

$$\begin{aligned} {\widetilde{B}}_k^\delta : = \Big (B_k{\setminus } \bigcup \limits _{j=1}^N Q_{x_j}\Big ) \cup \bigcup \limits _{j=1}^N(\overline{\Omega }\cap \partial U_{r_j,\nu _\Sigma (x_j)}(x_j)). \end{aligned}$$

Then, as in the proof of (3.42),

$$\begin{aligned} \mathcal {S}(B_k,u_k) - \mathcal {S}({\widetilde{B}}_k^\delta ,u_k) \geqq -4\delta (1+c_2)\sum \limits _{j=1}^N \mathcal {H}^1(U_{r_j,\nu _\Sigma (x_j)}(x_j)\cap \Sigma ), \end{aligned}$$

so that by the pairwise disjointness of $\{ U_{r_j,\nu _\Sigma (x_j)}(x_j)\},$

3.50 $$\begin{aligned} \mathcal {S}(B_k,u_k) - \mathcal {S}({\widetilde{B}}_k^\delta ,u_k) \geqq -4\delta (1+c_2)\mathcal {H}^1(\Sigma ). \end{aligned}$$

Recalling (3.49) and (3.38), and using (2.4), we estimate

$$\begin{aligned}&\mathcal {S}({\widetilde{B}}_k^\delta ,u_k+a_k) - \mathcal {S}({\widetilde{B}}_k^\delta ,u_k)\\&\quad \leqq \int _{L{\setminus } \bigcup _j U_{r_j,\nu _\Sigma (x_j)}(x_j)} \varphi (y,\nu _\Sigma )\text {d}\mathcal {H}^1 + \int _{J_{u_k+a_k}{\setminus } \partial ^*A} \varphi (y,\nu _\Sigma )\text {d}\mathcal {H}^1<2c_2\delta , \end{aligned}$$

hence, from (3.50), we get

3.51 $$\begin{aligned} \mathcal {S}(B_k,u_k) - \mathcal {S}({\widetilde{B}}_k^\delta ,u_k+a_k) \geqq -4\delta (1+c_2)\mathcal {H}^1(\Sigma ) - 2c_2\delta . \end{aligned}$$

On the other hand, since $\mathrm {Int}({\widetilde{B}}_k^\delta ) \subset \mathrm {Int}(B_k)$ and $e(u_k) = e(u_k+a_k),$

3.52 $$\begin{aligned} \mathcal {W}(B_k,u_k) \geqq \mathcal {W}({\widetilde{B}}_k^\delta ,u_k+a_k). \end{aligned}$$

From (3.51) and (3.52) we deduce

$$\begin{aligned} \mathcal {F}(B_k,u_k) \geqq \mathcal {F}({\widetilde{B}}_k^\delta ,u_k+a_k) -4\delta (1+c_2)\mathcal {H}^1(\Sigma ) - 2c_2\delta . \end{aligned}$$

However, by construction, $|B_k|\geqq |{\widetilde{B}}_k^\delta |$ since ${\widetilde{B}}_k^\delta \subset B_k\cup \bigcup \limits _{j=1}^N(\overline{\Omega }\cap \partial U_{r_j,\nu _\Sigma (x_j)}(x_j)).$ Thus, $R_k:=B_k{\setminus } {\widetilde{B}}_k^\delta$ satisfies $|R_k|=|B_k\Delta {\widetilde{B}}_k^\delta |.$ Since $\bigcup _jQ_{x_j} \subset \Omega \cap \{\mathrm {dist}(\cdot ,\Sigma )<\frac{t_\delta }{8}\},$ thus, by (3.39), $|R_k|<\delta ^2\epsilon _0.$ Hence, we choose a square $U_k\subset U$ (see (3.37)) such that $|U_k|=|R_k|.$ For $k>k_\delta$ set

$$\begin{aligned} B_k^\delta := {\widetilde{B}}_k^\delta \cup U_k. \end{aligned}$$

In order not to increase the number of connected components of $B_k^\delta$, we translate $U_k$ in $\Omega {\setminus }{\overline{B_k}}$ until it touches to $\partial {\widetilde{B}}_k^\delta .$ Define

$$\begin{aligned} u_k^\delta :=u_k\chi _{{\widetilde{B}}_k^\delta } + u_0\chi _{U_k}. \end{aligned}$$

Then $\{(B_k^\delta ,u_k^\delta )\}\subset \mathcal {C}_m$ and for any $k>k_\delta$ by (3.51) and (3.52)

$$\begin{aligned} \mathcal {F}(B_k,u_k)\geqq \mathcal {F}(B_k^\delta ,u_k^\delta ) - \mathcal {S}(U_k,u_0) -4\delta (1+c_2)\mathcal {H}^1(\Sigma ) - 2c_2\delta . \end{aligned}$$

By the choice of $U_k,$ its sidelength is less that $\delta \epsilon _0,$ hence, using $\epsilon _0<\frac{1}{2}$ and (2.4), $\mathcal {S}(U_k,u_0) \leqq 2c_2\delta$ so that

$$\begin{aligned} \mathcal {F}(B_k,u_k)\geqq \mathcal {F}(B_k^\delta ,u_k^\delta ) -4\delta (1+c_2)\mathcal {H}^1(\Sigma ) - 4c_2 \delta . \end{aligned}$$

*Step 3: Construction of*$$(D_n,v_n).$$ Notice that the sequence $$\{(B_k^\delta ,u_k^\delta )\}$$ in general does not need to satisfy $$B_k^\delta \overset{\tau _\mathcal {A}}{\rightarrow }A,$$ since we removed “something” from $$B_k$$ and added a square $$U_k.$$ To overcome this problem, we take $$\delta =\delta _n:=\frac{1}{16n}$$ and $$(D_n,v_n):=(B_{k_n}^{\delta _n},u_{k_n}^{\delta _n}),$$ where $$k_n:=k_{{\delta _n}}+1,$$ and there is no loss of generality in assuming $$n\mapsto k_n$$ is increasing. Denote $$r_j^n:=r_{x_j}^{\delta _n},$$ where the latter is defined in Substep 2.3 and notice that by (3.39) and (3.40) $$r_j^n\rightarrow 0$$ as $$n\rightarrow \infty $$ In particular, $$\partial D_n\overset{K}{\rightarrow }\partial A$$ as $$n\rightarrow \infty .$$ Thus, $$D_n\overset{\tau _\mathcal {A}}{\rightarrow }A.$$ Since $$|D_n\Delta A|\rightarrow 0,$$$$v_n\rightarrow u$$ a.e. in $$A\cup S.$$ By (3.36)

3.53 $$\begin{aligned} \mathcal {F}(B_{k_n},u_{k_n}) + \frac{(1+c_2)\mathcal {H}^1(\Sigma ) + c_2}{4n} \geqq \mathcal {F}(D_n,u_n), \end{aligned}$$

thus (2.9) follows from (3.53) and (3.34). $\quad \square$

## Lower Semicontinuity

In this section we consider more general surface energies. For every $A\in \mathcal {A}$ and $J_A\in \mathcal {J}_A,$ where

$$\begin{aligned} \mathcal {J}_A:= \big \{J\subseteq \Sigma \cap \overline{\partial ^* A}:\,\,J \text { is } \mathcal {H}^1-\text {measurable} \big \} \end{aligned}$$

is the collection of all possible delaminations on $\Sigma ,$ we define

$$\begin{aligned} \mathcal {S}(A,J_A; \varphi , g)&:= \int _{\Omega \cap \partial ^*A} \varphi (x,\nu _A)\text {d}\mathcal {H}^1 +2\int _{\Omega \cap (A^{(1)}\cup A^{(0)})\cap \partial A} \varphi (x,\nu _A)\text {d}\mathcal {H}^1 \nonumber \\&\quad + \int _{\Sigma {\setminus } \partial A} g(x,0)\text {d}\mathcal {H}^1 + \int _{\Sigma \cap A^{(0)}\cap \partial A} \big (\varphi (x,\nu _\Sigma ) + g(x,1)\big )\text {d}\mathcal {H}^1\nonumber \\&\quad + \int _{\Sigma \cap \partial ^*A{\setminus } J_A} g(x,1)\text {d}\mathcal {H}^1 + \int _{J_A} \big (\varphi (x,\nu _\Sigma ) + g(x,0) \big )\,\text {d}\mathcal {H}^1, \end{aligned}$$

where $g:\Sigma \times \{0,1\}\rightarrow \mathbb {R}$ is a Borel function. We remark that $\mathcal {S}(A,u) = \mathcal {S}(A,J_u;\varphi ,g)$ with $g(x,s)=\beta (x)s$ and $J_A = J_u.$

The main result of this section is the following.

### Proposition 4.1

(Lower-semicontinuity of $\mathcal {S}$) Suppose that $g:\Sigma \times \{0,1\}\rightarrow \mathbb {R}$ is a Borel function such that $g(\cdot ,s)\in L^1(\Sigma )$ for $s=0,1$ and

4.1 $$\begin{aligned} |g(x,1) - g(x,0)| \leqq \varphi (x,\nu _\Omega (x)) \end{aligned}$$

for $$\mathcal {H}^1$$-a.e. $$x\in \Sigma .$$ Let $$A_k\in \mathcal {A}_m,$$$$J_{A_k}\in \mathcal {J}_{A_k},$$$$A\in \mathcal {A}_m$$ and $$J_A\in \mathcal {J}_A$$ be such that

- $$A_k\overset{\tau _{\mathcal {A}}}{\rightarrow } A$$ as $$k\rightarrow \infty ;$$
- $$\mathcal {H}^1$$-a.e. $$x\in J_A$$ there exist $$r=r_x>0,$$$$w,w_k\in GSBD^2(B_r(x);\mathbb {R}^2)$$ and relatively open subset $$L_k$$ of $$\Sigma $$ with $$\mathcal {H}^1(L_k)<1/k$$ for which
  4.2 $$\begin{aligned} {\left\{ \begin{array}{ll} J_{w_k}\subset B_r(x)\cap (J_{A_k}\cup (\Omega \cap \partial A_k)\cup L_k) \text { and } J_A\subset J_w;\\ w_k\rightarrow w \text { a.e. in } B_r(x) \text { as } k\rightarrow \infty ;\\ \sup \limits _{k\geqq 1} \int _{B_r(x)}|e(w_k)|^2\text {d}x<\infty . \end{array}\right. } \end{aligned}$$

Then

4.3 $$\begin{aligned} \liminf \limits _{k\rightarrow \infty } \mathcal {S}(A_k,J_{A_k}; \varphi , g) \geqq \mathcal {S}(A,J_A; \varphi , g). \end{aligned}$$

We prove Proposition 4.1 using a blow-up around the points of the boundary of *A*. Given $y_o\in \mathbb {R}^2$ and $\rho >0,$ the blow-up map $\sigma _{\rho ,y_o}:\mathbb {R}^2\rightarrow \mathbb {R}^2$ is defined as

4.4 $$\begin{aligned} \sigma _{\rho ,y_o}(y):=\frac{y - y_o}{\rho }. \end{aligned}$$

When $$y_o=0$$ we write $$\sigma _\rho $$ instead of $$\sigma _{\rho ,0}.$$ Given $$\nu \in \mathbb {S}^1,$$$$ U_{\rho ,\nu }(x) $$ is an open square of sidelength $$2\rho >0$$ centered at *x* whose sides are either perpendicular or parallel to $$\nu ;$$ if $$\nu = \mathbf{e_2}$$ and $$x=0,$$ we write $$U_{\rho ,\nu }(0):=U_\rho =(-\rho ,\rho )^2,$$$$U_\rho ^+=(-\rho ,\rho )\times (0,\rho ),$$ and $$I_\rho :=[-\rho ,\rho ]\times \{0\}.$$ Observe that $$\sigma _{\rho ,x}(U_{\rho ,\nu }(x))=U_{1,\nu }(0)$$ and $$\sigma _{\rho ,x}(\overline{U_{\rho ,\nu }(x)})= \overline{U_{1,\nu }(0)}.$$ We denote by $$\pi $$ the projection onto $$x_1$$-axis i.e.,

4.5 $$\begin{aligned} \pi (x)=(x_1,0). \end{aligned}$$

The following auxiliary results will be used in the proof of Proposition 4.1:

### Lemma 4.2

Let *U* be any open square, $K\subset \overline{U}$ be a nonempty closed set and $E_k\subset U$ be such that $\mathrm {sdist}(\cdot ,\partial E_k)\overset{k\rightarrow \infty }{\longrightarrow } \mathrm {dist}(\cdot ,K)$ uniformly in $\overline{U}.$ Then $E_k\overset{\mathcal {K}}{\rightarrow } K$ as $k\rightarrow \infty .$ Analogously if $\mathrm {sdist}(\cdot ,\partial E_k) \overset{k\rightarrow \infty }{\longrightarrow } -\mathrm {dist}(\cdot ,K)$ uniformly in $\overline{U},$ then $\overline{U}{\setminus } E_k\overset{\mathcal {K}}{\rightarrow } K$ as $k\rightarrow \infty .$

### Proof

We prove only the first assertion, the second being the same. If $x_k\in E_k$ is such that $x_k\rightarrow x,$ then by assumption,

$$\begin{aligned} \mathrm {dist}(x,K) = \lim \limits _{k\rightarrow \infty } \mathrm {sdist}(x_k,\partial E_k) \leqq 0 \end{aligned}$$

so that $x\in K.$ On the other hand, given $x\in K$ suppose that there exists $r>0$ such that $B_r(x)\cap E_k=\emptyset$ for infinitely many *k*. Then for such *k*,  $\mathrm {sdist}(x,\partial E_k) = \mathrm {dist}(x,E_k)\geqq r>0,$ which contradicts to the assumption.   $\square$

In the next lemma we observe that the endpoints of every curve $\Gamma$ contained in the boundary of any bounded set *A* with connected boundary are still arcwise connected if we remove the boundary of $\mathrm {Int}(\overline{A})$ belonging to $\Gamma .$

### Lemma 4.3

Let $A\subset \mathbb {R}^2$ be a bounded set such that $\partial A$ is connected and has finite $\mathcal {H}^1$ measure. Suppose that $x,y\in \partial A$ are such that $x\ne y$ and $\Gamma \subset \partial A$ is a curve connecting *x* to *y*. Then there exists a curve $\Gamma '\subset \overline{\partial A{\setminus } (\Gamma \cap \partial \mathrm {Int}(\overline{A}))}$ connecting *x* to *y*.

### Proof

Without loss of generality we assume $G:=\mathrm {Int}(\overline{A})\ne \emptyset ,$ otherwise we simply take $\Gamma '=\Gamma .$ Note that

4.6 $$\begin{aligned} \partial G = \big \{x\in \partial A:\,\, B_r(x)\cap \mathrm {Int}(A),B_r(x){\setminus } \overline{A} \ne \emptyset \,\,\hbox { for every}\ r>0\big \}. \end{aligned}$$

Since connected compact sets of finite length are arcwise connected (see Proposition A.2), it suffices to show that *x* and *y* belong to the same connected component of $\overline{\partial A{\setminus } (\Gamma \cap \partial G)}.$ Suppose that there exist two open sets $P,Q\subset \mathbb {R}^2$ with disjoint closures such that

4.7 $$\begin{aligned} \overline{\partial A{\setminus } (\Gamma \cap \partial G)} = (P\cap \overline{\partial A{\setminus } (\Gamma \cap \partial G)}) \cup (Q\cap \overline{\partial A{\setminus } (\Gamma \cap \partial G)}), \end{aligned}$$

where $x\in P\cap \overline{\partial A{\setminus } (\Gamma \cap \partial G)}$ and $y\in Q\cap \overline{\partial A{\setminus } (\Gamma \cap \partial G)}.$ Then $\Gamma {\setminus } \overline{P\cup Q}\ne \emptyset$ and

4.8 $$\begin{aligned} \Gamma {\setminus } \overline{P\cup Q} = \partial G {\setminus } \overline{P\cup Q}. \end{aligned}$$

Since $\overline{P}\cap \overline{Q}=\emptyset$ and $\mathcal {H}^1(\Gamma )<\infty ,$ the number of connected components $\{L_i\}_{i=1}^n$ of $\Gamma {\setminus }\overline{P\cup Q}$ connecting both *P* and *Q* is at most finite. Moreover, since $\Gamma$ has no self-intersections (see Section A.2 for the definition of the curve in our setting) and the endpoints of $\Gamma$ belong to *P* and *Q*,  respectively, *n* must be odd. However, by (4.8) $L_i\subset \partial G,$ and hence, by (4.6), every neighborhood of $L_i$ contains points belonging to both $\mathrm {Int}(A)$ and $\mathbb {R}^2{\setminus } \overline{A}.$ We reached a contradiction since in this case $\mathrm {Int}(A)$ would be unbounded.   $\quad \square$

Notice that if $A\in \mathcal {A}_m,$ then $\overline{\partial ^* A} = \partial A^{(1)} =\partial \mathrm {Int}(\overline{A}).$

### Lemma 4.4

(Creation of external filament energy) Let $\phi$ be a norm in $\mathbb {R}^2$ satisfying

4.9 $$\begin{aligned} c_1\leqq \phi (\nu ) \leqq c_2,\qquad \nu \in \mathbb {S}^1 \end{aligned}$$

for some $c_2\geqq c_1>0,$ and let $\{E_k\}$ be a sequence of subsets of $U_1$ such that

- $$E_k \overset{\mathcal {K}}{\rightarrow }I_1$$ as $$k\rightarrow \infty ;$$
- there exists $m_o\in \mathbb {N}_0$ such that the number of connected components of $\partial E_k$ lying strictly inside $U_1$ does not exceed $m_o.$

Then, for every $\delta \in (0,1)$, there exists $k_\delta >1$ such that for any $k>k_\delta ,$

4.10 $$\begin{aligned} \int _{U_1\cap \partial ^* E_k} \phi (\nu _{E_k})\,\text {d}\mathcal {H}^1 +2 \int _{U_1\cap (E_k^{(1)}\cup E_k^{(0)})\cap \partial E_k} \phi (\nu _{E_k})\,\text {d}\mathcal {H}^1 \geqq 2\int _{I_1}\phi (\mathbf{e_2})\,\text {d}\mathcal {H}^1- \delta . \end{aligned}$$

### Proof

Let us denote the left hand side of (4.10) by $\alpha _k.$ We may suppose $\sup _k\alpha _k<\infty$. By assumption (a), for every $\delta \in (0,1)$ there exists $k_{1,\delta }>1$ such that

$$\begin{aligned} E_k\subset [-1,1]\times \Big (-\frac{\delta }{16c_2m_o}, \frac{\delta }{16c_2m_o}\Big ) \end{aligned}$$

for all $$k>k_{1,\delta }.$$

*Step 1.* Assume that for some $$k > k_{1,\delta },$$$$\partial E_k$$ has a connected component $$K^1$$ intersecting both $$\{x_1=1\}$$ and $$\{x_1=-\,1\}.$$ In this case by Lemma 4.3, $$\overline{\partial E_k{\setminus } (K^1\cap \partial \mathrm {Int}(\overline{A}))}$$ is also connected and contains a path $$K^2$$ connecting $$\{x_1=1\}$$ to $$\{x_1=-\,1\}.$$ Note that $$K^1$$ and $$K^2$$ may coincide on $$(E_k^{(1)}\cup E_k^{(0)})\cap \partial E_k.$$ Let $$R_i^1$$ and $$R_i^2,$$$$i=1,2,$$ be the segments along the vertical lines $$\{x_1=\pm 1\}$$ connecting the endpoints of $$K^1$$ and $$K^2$$ to $$(\pm 1,0),$$ respectively. Since $$K^1\cap \partial ^*E_k$$ and $$K^2\cap \partial ^*E_k$$ are disjoint up to a $$\mathcal {H}^1$$-negligible set

4.11 $$\begin{aligned} \alpha _k&\geqq \sum \limits _{j=1}^2\Big (\int _{K^j\cap \partial ^*E_k} \phi (\nu _{E_k})\,\text {d}\mathcal {H}^1 + \int _{{K^j{\setminus } \partial ^*E_k}} \phi (\nu _{E_k})\,\text {d}\mathcal {H}^1\Big )\nonumber \\&= \sum \limits _{j=1}^2 \int _{\gamma _j} \phi (\nu _{\gamma _j})\,\text {d}\mathcal {H}^1 - \sum \limits _{i,j=1}^2 \int _{R_i^j} \phi (\mathbf{e_1})\,\text {d}\mathcal {H}^1, \end{aligned}$$

where $\gamma _j:=R_1^j\cup K^j\cup R_2^j$ is the curve connecting $(-1,0)$ to (1, 0). By the (anisotropic) minimality of segments [30, Lemma 6.2],

4.12 $$\begin{aligned} \int _{\gamma _j} \phi (\nu _{\gamma _j})\,\text {d}\mathcal {H}^1\geqq \int _{I_1} \phi (\mathbf{e_2})\,\text {d}\mathcal {H}^1 \end{aligned}$$

Moreover, since $\mathcal {H}^1(R_i^j) \leqq \frac{\delta }{16c_2m_o}$ for any $i,j=1,2,$ by (4.11), (4.12) and (2.4) we obtain

4.13 $$\begin{aligned} \alpha _k \geqq 2 \int _{I_1} \phi (\mathbf{e_2})\,\text {d}\mathcal {H}^1 - \frac{4c_2\delta }{16c_2m_o} = 2 \int _{I_1} \phi (\mathbf{e_2})\,\text {d}\mathcal {H}^1 - \frac{\delta }{4 m_o}, \end{aligned}$$

which implies (4.10).

*Step 2.* Assume now that every connected component of $$U_1\cap \partial E_k$$ intersects at most one of $$\{x_1=1\}$$ and $$\{x_1=-\,1\}.$$ In this case, let $$K^{1},\ldots ,K^{m_k}$$ stand for the connected components of $$\partial E_k$$ lying strictly inside of $$U_1$$ (that is, not intersecting $$\{x_1=\pm 1\}$$); by (b), $$m_k\leqq m_o.$$ Since $$\alpha _k<\infty ,$$ the connected components $$\{L^i\}$$ of $$\overline{U_1}\cap \partial E_k$$ intersecting $$\{x_1=\pm 1\}$$ is at most countable. If $$\{L^i:\,L^i\cap \{x_1=1\}\ne \emptyset \}=\emptyset $$, we set $$K^{m_k+1}=\emptyset $$ otherwise let $$K^{m_k+1}$$ be such that $$\pi (K^{m_k+1})$$ contains all $$\pi (L^i)$$ with $$L^i\cap \{x_1=1\}\ne \emptyset ,$$ where $$\pi $$ is given by (4.5). Analogously, we define $$K^{m_k+2}\in \{L^i:\,L^i\cap \{x_1=-\,1\}\ne \emptyset \}\cup \{\emptyset \}.$$ By the connectedness of $$K^j,$$ for each $$j=1,\ldots ,m_k+2,$$$$\pi (K^j)$$ (if non-empty) is a segment $$[a_k^i,b_k^i]\times \{0\}.$$ Then assumption (a) and the bound $$m_k\leqq m_o$$ imply that

$$\begin{aligned} \lim \limits _{k\rightarrow \infty } \mathcal {H}^1\Big (I_1{\setminus } \bigcup \limits _{j=1}^{m_k+2} \pi (K^j)\Big )=0. \end{aligned}$$

Hence there exists $k_{2,\delta }>k_{1,\delta }$ such that

4.14 $$\begin{aligned} \mathcal {H}^1\Big (I_1 {\setminus } \bigcup \limits _{i=1}^{m_k+2} \pi (K^j)\Big ) < \frac{\delta }{8c_2m_o} \end{aligned}$$

for any $k> k_{2,\delta }.$ Then repeating the proof of (4.13) with $K^j$ in $(a_j,b_j)\times (-1,1)$, for every $j=1,\ldots ,m_k+2$ we find

$$\begin{aligned} \int _{K^j\cap \partial ^*E_k} \phi (\nu _{E_k})\text {d}\mathcal {H}^1 +2 \int _{K^j\cap (E_k^{(1)}\cup E_k^{(0)})\cap \partial E_k} \phi (\nu _{E_k})\text {d}\mathcal {H}^1 \geqq 2 \int _{\pi (K^j)} \phi (\mathbf{e_2})\,\text {d}\mathcal {H}^1 - \frac{\delta }{4m_o}. \end{aligned}$$

Therefore, by (4.14) and (2.4),

$$\begin{aligned} \alpha _k&\geqq \sum \limits _{j=1}^{m_k+2} \int _{K^j\cap \partial ^*E_k} \phi (\nu _{E_k})\text {d}\mathcal {H}^1 +2 \int _{K^j\cap (E_k^{(1)} \cup E_k^{(0)})\cap \partial E_k} \phi (\nu _{E_k})\text {d}\mathcal {H}^1 \\&\geqq \sum \limits _{j=1}^{m_k+2} \Big (2 \int _{\pi (K^j)} \phi (\mathbf{e_2})\,\text {d}\mathcal {H}^1 - \frac{\delta }{4m_o}\Big )\geqq 2 \int _{\bigcup \pi (K^j)} \phi (\mathbf{e_2})\,\text {d}\mathcal {H}^1 - \frac{(m_k+2)\delta }{4m_o} \\&\geqq 2\int _{I_1} \phi (\mathbf{e_2})\,\text {d}\mathcal {H}^1 - 2c_2\,\frac{\delta }{8c_2m_o}- \frac{(m_o+2)\delta }{4m_o} \\&= 2\int _{I_1} \phi (\mathbf{e_2})\,\text {d}\mathcal {H}^1 - \frac{(m_o+3)\delta }{4m_o}. \end{aligned}$$

Since $m_o\geqq 1,$ this implies (4.10).   $\square$

### Lemma 4.5

(Creation of internal crack energy)

Let $\phi$ be as in Lemma 4.4 and let $\{E_k\}$ be a sequence of subsets of $U_1$ such that

- $$U_1{\setminus } E_k \overset{\mathcal {K}}{\rightarrow }I_1=[-1,1]\times \{0\}$$ as $$k\rightarrow \infty ;$$
- there exists $m_o\in \mathbb {N}_0$ such that the number of connected components of each $\partial E_k$ lying strictly inside $U_1$ does not exceed $m_o.$

Then for every $\delta \in (0,1)$ there exists $k_\delta >1$ such that for any $k>k_\delta ,$

$$\begin{aligned} \int _{U_1\cap \partial ^* E_k} \phi (\nu _{E_k})\,\text {d}\mathcal {H}^1 +2 \int _{U_1\cap (E_k^{(0)}\cup E_k^{(1)})\cap \partial E_k} \phi (\nu _{E_k})\,\text {d}\mathcal {H}^1 \geqq 2\int _{I_1}\phi (\mathbf{e_2})\,\text {d}\mathcal {H}^1- \delta . \end{aligned}$$

### Proof

The assertion follows from applying Lemma 4.5 to $$U_1{\setminus } E_k.$$$$\quad \square $$

The following result extends the lower semicontinuity result of [15, Theorem 1.1] to the anisotropic case.

### Proposition 4.6

Let $$D\subset \mathbb {R}^d$$ be a bounded open set and let $$\phi \in C(\overline{D}\times \mathbb {R}^d;[0,+\infty )$$ be a Finsler norm in $$\mathbb {R}^d,$$$$d\geqq 2,$$ satisfying

4.15 $$\begin{aligned} c_1\leqq \phi (x,\nu ) \leqq c_2,\qquad (x,\nu )\in \overline{D}\times \mathbb {S}^{d-1}, \end{aligned}$$

for some $c_2\geqq c_1>0.$ Consider $\{w_h\}\subset GSBD^2(D;\mathbb {R}^d)$ such that

4.16 $$\begin{aligned} \sup \limits _{k\geqq 1} \int _D|e(w_h)|^2\text {d}x +\mathcal {H}^1(J_{w_h})\leqq M \end{aligned}$$

for some $M>0$ and the set

$$\begin{aligned} E:=\{x\in D:\,\,\liminf \limits _{h\rightarrow \infty } |w_h(x)| = +\infty \} \end{aligned}$$

has finite perimeter. Suppose that $w_h\rightarrow w$ a.e. in $D{\setminus } E$ as $h\rightarrow \infty$ (so that by [15, Theorem 1.1] $w\in GSBD^2(D{\setminus } E;\mathbb {R}^d)$). Then

4.17 $$\begin{aligned} \int _{J_w\cup \partial ^*E} \phi (x,\nu _{J_w\cup \partial E}^{})\text {d}\mathcal {H}^{d-1} \leqq \liminf \limits _{h\rightarrow \infty } \int _{J_{w_h}} \phi (x,\nu _{J_{w_h}}^{})\text {d}\mathcal {H}^{d-1}. \end{aligned}$$

### Proof

We divide the proof into two steps.

*Step 1.* First we prove the (4.17) assuming that $\phi$ is independent on $x\in D,$ i.e., $\phi (\nu )=\phi (x,\nu )$ for any $x\in \overline{D}\times \mathbb {R}^d.$

Let $W=\{\phi ^\circ \leqq 1\}$ be the Wulff shape of $\phi$, i.e, the unit ball for the dual norm

$$\begin{aligned} \phi ^\circ (\xi ) = \max \limits _{\phi (\eta )=1}\,\, |\xi \cdot \eta |. \end{aligned}$$

Note that $\phi ^{\circ \circ } = \phi$ and by (4.15),

4.18 $$\begin{aligned} \frac{1}{c_2}\,|\xi |\leqq \phi ^\circ (\xi )\leqq \frac{1}{c_1}\,|\xi | \end{aligned}$$

for any $\xi \in \mathbb {R}^2.$ Let $\{\xi _n\}\subset \partial W$ be a countable dense set. Then since

$$\begin{aligned} \phi (\nu ) = \sup \limits _{n\in \mathbb {N}} |\xi _n\cdot \nu |, \end{aligned}$$

from [25, Lemma 6] it follows that for every bounded open set *G* and $u\in GSBD^2(G;\mathbb {R}^d),$

4.19 $$\begin{aligned} \int _{G\cap J_u} \phi (\nu _{J_u}^{})\text {d}\mathcal {H}^{d-1} = \sup \,\sum \limits _{n=1}^N \int _{F_n\cap J_u} |\xi _n\cdot \nu _{J_u}^{}| \text {d}\mathcal {H}^{d-1}, \end{aligned}$$

where $\sup$ is taken over finite disjoint open sets $\{F_n\}_{n=1}^N$ whose closures are contained in *G*.

Now we prove (4.17). Under the notation of [7, 15], for any $\epsilon \in (0,1),$ open set $F\subset D$ with $\overline{F}\subset D$ and for $\mathcal {H}^1$-a.e. $\xi \in \partial W$ we have

4.20 $$\begin{aligned} |\xi | \int _{\Pi _\xi } \Big [\mathcal {H}^0(F_y^\xi&\cap J_{\widehat{w}_y^\xi }\cap (F{\setminus } E)_y^\xi ) + \mathcal {H}^0(F_y^\xi \cap \partial E_y^\xi )\Big ]\text {d}\mathcal {H}^{d-1} \nonumber \\&\leqq |\xi | \liminf \limits _{h\rightarrow \infty } \int _{\Pi _\xi } \Big [\mathcal {H}^0(F_y^\xi \cap J_{({\widehat{w}}_h)_y^\xi }) + c_2^{-1} \epsilon f_y^\xi (w_h) \Big ]\text {d}\mathcal {H}^{d-1}, \end{aligned}$$

where $$ \Pi _\xi : = \{y\in \mathbb {R}^d:\,\, y\cdot \xi = 0\}, $$ is the hyperplane passing through the origin and orthogonal to $$\xi ,$$ given $$y\in \mathbb {R}^d,$$$$ F_y^\xi :=\{t\in \mathbb {R}:\,\, y+t\xi \in F\} $$ is the section of the straight line passing through $$y\in \mathbb {R}^d$$ and parallel to $$\xi ,$$ given $$u:F\rightarrow \mathbb {R}^d$$ and $$y\in \mathbb {R}^d,$$$${\widehat{u}}_y^\xi :F_y^\xi \rightarrow \mathbb {R}$$ is defined as $${\widehat{u}}_y^\xi (t): = u(y+t\xi )\cdot \xi ,$$ and

4.21 $$\begin{aligned} f_y^\xi (w_h) = I_y^\xi (w_h) + II_y^\xi (w_h), \end{aligned}$$

with

$$\begin{aligned} \int _{\Pi _\xi } I_y^\xi (w_h) \text {d}\mathcal {H}^1\leqq \int _F |e(w_h(x))|^2\text {d}x, \qquad h\geqq 1, \end{aligned}$$

and

$$\begin{aligned} \int _{\Pi _\xi } II_y^\xi (w_h) \text {d}\mathcal {H}^1\leqq |D_\xi (\tau (w_h\cdot \xi ))|(F), \qquad h\geqq 1, \end{aligned}$$

for $\tau (t):= \tanh (t)$ (see [15, Eq.s 3.10 and 3.11] applied with *F* in place of $\Omega$).

By [2, Theorem 4.10] and (4.18), (4.20) can be rewritten as

4.22 $$\begin{aligned}&\int _{F\cap (J_w\cup \partial ^*E)} |\nu _{J_w\cup \partial E}^{}\cdot \xi | \text {d}\mathcal {H}^{d-1} \leqq \liminf \limits _{h\rightarrow \infty } \int _{F\cap J_{w_h}} |\nu _{J_{w_h}}^{}\cdot \xi |\text {d}\mathcal {H}^{d-1}\nonumber \\&\quad + \epsilon \int _F |e(w_h(x))|^2\text {d}x + \epsilon |D_\xi (\tau (w_h\cdot \xi ))|(F). \end{aligned}$$

Fix any finite family $\{F_n\}_{n=1}^N$ of pairwise disjoint open sets whose closures are contained in *D*. Since (4.22) holds for $\mathcal {H}^1$-a.e. $\xi \in \partial W,$ we can extract a countable dense set $\{\xi _n\}\subset \partial W$ satisfying (4.22) with $\xi =\xi _i$ and $F=F_j$ for all *i*, *j*. Now taking $F=F_n$ and $\xi =\xi _n$ in (4.22) and summing over $n=1,\ldots ,N,$ we get

$$\begin{aligned}&\sum \limits _{n=1}^N \int _{F_n\cap (J_w\cup \partial ^*E)} |\nu _{J_w\cup \partial E}^{}\cdot \xi _n| \text {d}\mathcal {H}^{d-1} \leqq \liminf \limits _{h\rightarrow \infty } \sum \limits _{n=1}^N \int _{F_n\cap J_{w_h}} |\nu _{J_{w_h}}^{}\cdot \xi _n|\text {d}\mathcal {H}^{d-1}\\&\quad +\epsilon \int _{\bigcup \limits _{n=1}^NF_n} |e(w_h(x))|^2\text {d}x + \epsilon |D_{\xi _n}(\tau (w_h\cdot \xi _n))|\Big (\bigcup \limits _{n=1}^NF_n\Big ). \end{aligned}$$

Recall that by (4.19),

$$\begin{aligned} \sum \limits _{n=1}^N \int _{F_n\cap J_{w_h}} |\nu _{J_{w_h}}^{}\cdot \xi _n|\text {d}\mathcal {H}^1 \leqq \int _{J_{w_h}} \phi (\nu _{J_{w_h}}^{})\mathcal {H}^{d-1}, \end{aligned}$$

and by (4.16),

$$\begin{aligned} \int _{\bigcup \limits _{n=1}^NF_n} |e(w_h(x))|^2\text {d}x \leqq \int _{D} |e(w_h(x))|^2\text {d}x \leqq M \end{aligned}$$

and

$$\begin{aligned} |D_{\xi _n}(\tau (w_h\cdot \xi _n))|\Big (\bigcup \limits _{n=1}^NF_n\Big ) \leqq |D_{\xi _n}(\tau (w_h\cdot \xi _n))|(D) \leqq M \end{aligned}$$

for any $h\geqq 1.$ Therefore,

$$\begin{aligned} \sum \limits _{n=1}^N \int _{F_n\cap (J_w\cup \partial ^*E)} |\nu _{J_w\cup \partial E}^{}\cdot \xi _n| \text {d}\mathcal {H}^{d-1} \leqq 2M\epsilon + \liminf \limits _{h\rightarrow \infty } \int _{J_{w_h}} \phi (\nu _{J_{w_h}}^{})\mathcal {H}^{d-1}. \end{aligned}$$

Now taking $\sup$ over $\{F_n\}$ and letting $\epsilon \rightarrow 0$ we obtain (4.17).

*Step 2.* Now we prove (4.17) in general case. Without loss of generality we suppose that the *liminf* in (4.17) is a finite limit. Consider the sequence $\{\mu _h\}_{h\geqq 0}$ of positive Radon measures in *D* defined at Borel subsets of $B\subseteq D$ as

$$\begin{aligned} \mu _h(B) = \int _{B\cap J_{w_h}} \phi (x,\nu _{J_{w_h}}^{})\text {d}\mathcal {H}^{d-1},\qquad h\geqq 1, \end{aligned}$$

and

$$\begin{aligned} \mu _0(B) = \int _{B\cap J_w} \phi (x,\nu _{J_w}^{})\text {d}\mathcal {H}^{d-1}. \end{aligned}$$

Since $\sup _h \mu _h(D)<\infty ,$ by compactness, there exist a positive Radon measure $\mu$ and a not relbelled subsequence $\{\mu _h\}_{h\geqq 1}$ such that $\mu _h\rightharpoonup *\mu$ as $h\rightarrow \infty .$ We prove that

4.23 $$\begin{aligned} \mu \geqq \mu _0, \end{aligned}$$

in particular from $\mu (D) \geqq \mu _0(D)$ (4.17) follows. Since $\mu _0$ is absolutely continuous with respect to  to prove (4.23) we need only to show4.24For this aim fix $\epsilon \in (0,c_1).$ By the uniform continuity of $\phi ,$ there exists $r_\epsilon >0$ such that

4.25 $$\begin{aligned} |\phi (x,\nu ) - \phi (y,\nu )|\leqq \epsilon \end{aligned}$$

for any $\nu \in \mathbb {S}^{d-1}$ and $x,y\in D$ with $|x-y|<r_\epsilon .$ In particular, given $x\in J_w$ and for a.e. $r\in (0,r_\epsilon ),$

$$\begin{aligned} \mu (B_r(x_0))&= \lim \limits _{h\rightarrow \infty } \mu _h(B_r(x_0)) \\ \geqq&\liminf \limits _{h\rightarrow \infty } \int _{B_r(x_0)\cap J_{w_h}} \phi (x_0,\nu _{J_{w_h}})\text {d}\mathcal {H}^{d-1} - \epsilon \limsup \limits _{h\rightarrow \infty } \mathcal {H}^{d-1}(B_r(x_0)\cap J_{w_h}), \end{aligned}$$

where in the equality we use the weak* convergence of $\{\mu _h\}$ and in the inequality (4.25) with $y=x_0$ and $x\in B_r(x_0)\cap J_{w_h}$ By Proposition 4.6 applied with $\phi (x_0,\cdot ),$

$$\begin{aligned} \liminf \limits _{h\rightarrow \infty } \int _{B_r(x_0)\cap J_{w_h}} \phi (x_0,\nu _{J_{w_h}})\text {d}\mathcal {H}^{d-1}&\geqq \int _{B_r(x_0)\cap J_w} \phi (x_0,\nu _{J_w})\text {d}\mathcal {H}^{d-1}\\&\geqq \mu _0(B_r(x_0)) - \epsilon \mathcal {H}^{d-1}(B_r(x_0)\cap J_w), \end{aligned}$$

where in the second equality we again used (4.25). Moreover, by (4.15),

$$\begin{aligned} \limsup \limits _{h\rightarrow \infty } \mathcal {H}^{d-1}(B_r(x_0)\cap J_{w_h}) \leqq \frac{1}{c_1}\,\limsup \limits _{h\rightarrow \infty } \mu _h(B_r(x_0))= \frac{\mu (B_r(x_0))}{c_1} \end{aligned}$$

and

$$\begin{aligned} \mathcal {H}^{d-1}(B_r(x_0)\cap J_w) \leqq \frac{\mu _0(B_r(x_0))}{c_1}, \end{aligned}$$

thus,

$$\begin{aligned} \mu (B_r(x_0)) \geqq \frac{c_1-\epsilon }{c_1+\epsilon }\,\,\mu _0(B_r(x_0)). \end{aligned}$$

Since $\epsilon$ and $r\in (0,r_\epsilon )$ are arbitrary, (4.24) follows from the Besicovitch Derivation Theorem.   $\quad \square$

### Lemma 4.7

(Creation of delamination energy)

Let $$\phi $$ be as in Lemma 4.4 and suppose that $$\Omega _k\subset U_4$$ is a sequence of Lipschitz sets, $$E_k\subset \Omega _k,$$$$J_{E_k}\in \mathcal {J}_{E_k},$$ and $$g_0,g_1\in [0,+\infty ),$$$$u_k\in GSBD^2(U_4;\mathbb {R}^2)$$ and $$u^\pm \in \mathbb {R}^2$$ with $$u^+\ne u^-$$ are such that

- $$\mathrm {sdist}(\cdot , U_4 \cap \partial \Omega _k)\rightarrow \mathrm {sdist}(\cdot ,\partial U_4^+)$$ uniformly in $$U_{3/2}$$;
- $\Sigma _k:=U_4\cap \partial \Omega _k$ is a graph of a Lipschitz function $l_k:{I_4}\rightarrow \mathbb {R}$ such that $l_k(0)=0$ and $|l_k'|\leqq \frac{1}{k};$
- $$\mathrm {sdist}(\cdot , U_4 \cap \partial E_k)\rightarrow \mathrm {sdist}(\cdot ,\partial U_4^+)$$ uniformly in $$U_{3/2}$$;
- there exists $m_k\in \mathbb {N}_0$ such that the number of connected components of each $\partial E_k$ lying strictly inside $\Omega _k$ does not exceed $m_k;$
- $$|g_1 - g_0|\leqq \phi (\mathbf{e_2});$$
- $J_{u_k}\subset (\Omega _k\cap \partial E_k)\cup J_{E_k}\cup L_k,$ where $L_k\subset \Sigma _k$ is a relatively open subset of $\Sigma _k$ with $\mathcal {H}^1(L_k)<1/k;$
- $$\sup \limits _{k\geqq 1} \int _{U_4}|e(u_k)|^2\text {d}x < \infty ;$$
- $u_k\rightarrow u^+$ a.e. in $U_1^+$ and $u_k\rightarrow u^-$ a.e. in $U_1{\setminus } \overline{U_1^+}.$

Then for every $\delta \in (0,1)$ there exists $k_\delta >1$ for which

4.26 $$\begin{aligned}&\int _{U_1\cap \Omega _{k} \cap \partial ^*E_{k}} \phi (\nu _{E_{k}})\text {d}\mathcal {H}^1 +2\int _{U_1\cap \Omega _{k} \cap (E_{k}^{(1)}\cup E_k^{(0)}) \cap \partial E_{k}} \phi (\nu _{E_{k}})\text {d}\mathcal {H}^1\nonumber \\&\qquad + \int _{U_1\cap \Sigma _{k}{\setminus } \partial E_{k}} g_0 \text {d}\mathcal {H}^1 + \int _{U_1\cap \Sigma _{k}\cap E_{k}^{(0)}\cap \partial E_{k}} \big (\phi (\nu _\Sigma ) + g_1\big )\text {d}\mathcal {H}^1\nonumber \\&\qquad + \int _{U_1\cap \Sigma _{k}\cap \partial ^*E_{k}{\setminus } J_{E_{k}}} g_1\text {d}\mathcal {H}^1 + \int _{U_1\cap J_{E_{k}}} \big (\phi (\nu _\Sigma ) + g_0 \big )\,\text {d}\mathcal {H}^1\nonumber \\&\quad \geqq \int _{I_1}\big (\phi (\mathbf{e_2}) + g_0\big )\,\text {d}\mathcal {H}^1- \delta \end{aligned}$$

for any $$k>k_\delta .$$

### Proof

Denote the left-hand-side of (4.26) by $\alpha _k.$ We suppose that $\sup _k |\alpha _k|<\infty$ so that by (4.9)

4.27 $$\begin{aligned} \sup \limits _k \mathcal {H}^1(\partial E_k)\leqq M \end{aligned}$$

for some $M>0.$ Moreover, passing to a not relabelled subsequence if necessary, we assume that

$$\begin{aligned} \liminf \limits _{k\rightarrow \infty } \alpha _k = \lim \limits _{k\rightarrow \infty } \alpha _k. \end{aligned}$$

By assumption (b), $\Sigma _k$ is “very close” $I_2,$ hence, by the area formula [3, Theorem 2.91] for any $K_k\subset \Sigma _k$ one has

$$\begin{aligned}&\limsup \limits _{k\rightarrow \infty } \Big |\int _{\pi (K_k)}\phi (\mathbf{e_2})\text {d}\mathcal {H}^1 - \int _{K_k} \phi (\nu _{\Sigma _k})\text {d}\mathcal {H}^1 \Big |\\&\quad \leqq \limsup \limits _{k\rightarrow \infty } \int _{\pi (K_k)}|\phi (\mathbf{e_2}) -\phi (l_k',1)| \text {d}\mathcal {H}^1 \leqq \limsup \limits _{k\rightarrow \infty } \int _{\pi (K_k)}|\phi (\mathbf{e_2} -(l_k',1))| \text {d}\mathcal {H}^1 \\&\quad = \limsup \limits _{k\rightarrow \infty } \int _{\pi (K_k)}\phi (0,1)\,|l_k'| \text {d}\mathcal {H}^1 =0, \end{aligned}$$

where in the last inequality and in the first equality we used that $\phi$ is a norm, and the last equality follows from $|l_k'|\leqq \frac{1}{k}.$ Hence,

4.28 $$\begin{aligned} \lim \limits _{k\rightarrow \infty } \Big |\int _{\pi (K_k)}\phi (\mathbf{e_2})\text {d}\mathcal {H}^1 - \int _{K_k} \phi (\nu _{\Sigma _k})\text {d}\mathcal {H}^1 \Big | = 0. \end{aligned}$$

We divide the proof into two steps.

*Step 1.* For shortness, let $J_k:=J_{E_k}$ and $C_k:=\Sigma _k{\setminus }\overline{\partial ^*E_k}.$ We claim that for any $\delta \in (0,1)$ there exists $k_\delta ^1>0$ such that for any $k>k_\delta ^1,$

4.29 $$\begin{aligned}&\int _{U_1\cap \Omega _k \cap \partial ^*E_k} \phi (\nu _{E_k})\text {d}\mathcal {H}^1 +2\int _{U_1\cap \Omega _k \cap (E_{k}^{(1)}\cup E_k^{(0)}) \cap \partial E_k} \phi (\nu _{E_k})\text {d}\mathcal {H}^1 \nonumber \\&\quad \geqq 2 \int _{U_1\cap \Sigma _k{\setminus } (J_k\cup C_k)} \phi (\nu _{\Sigma _k})\text {d}\mathcal {H}^1 +\int _{U_1\cap C_k} \phi (\nu _{\Sigma _k})\text {d}\mathcal {H}^1- \delta . \end{aligned}$$

Indeed, by adding to both sides of (4.29) the quantity $2\int _{U_1\cap J_k} \phi (\nu _{\Sigma _k})\text {d}\mathcal {H}^1 +\int _{U_1\cap C_k} \phi (\nu _{\Sigma _k})\text {d}\mathcal {H}^1,$ (4.29) is equivalent to

4.30 $$\begin{aligned}&\int _{U_1\cap ((\Omega _k \cap \partial ^*E_k) \cup C_k)} \phi (\nu _{\partial E_k\cup C_k})\text {d}\mathcal {H}^1 +2\int _{U_1\cap \Omega _k \cap (((E_{k}^{(1)}\cup E_k^{(0)}) \cap \partial E_k)\cup J_k)} \phi (\nu _{E_k})\text {d}\mathcal {H}^1 \nonumber \\&\quad \geqq 2 \int _{U_1\cap \Sigma _k} \phi (\nu _{\Sigma _k})\text {d}\mathcal {H}^1- \delta , \end{aligned}$$

and hence, we will prove (4.30).

Note that since $J_k\subset \Sigma _k$ is $\mathcal {H}^1$-rectifiable, given $\delta \in (0,1)$ there exists a finite union $R_k$ of intervals of $\Sigma _k$ such that

4.31 $$\begin{aligned} J_k\cup L_k\subset R_k\quad \text {and}\qquad \mathcal {H}^1(R_k{\setminus } (J_k\cup L_k))<\frac{\delta }{5c_2}, \end{aligned}$$

where $$c_2>0$$ is given in (4.9). Possibly slightly modifying $$u_k$$ around the (approximate) continuity points of $$R_k$$ and around the boundary of the voids $$U_2{\setminus } E_k$$ we assume that $$J_k:=R_k,$$$$L_k=\emptyset $$ and $$J_{u_k}=(\Omega _k\cap \partial E_k)\cup C_k\cup J_k$$ (up to a $$\mathcal {H}^1$$-negligible set).

Let $K_k=U_1\cap (\overline{\Omega _k\cap \partial E_k}\cup \overline{J_k\cup C_k}).$ By relative openness of $C_k=\Sigma _k{\setminus } \overline{\partial ^*E_k}$ and $J_k$ in $\Sigma _k$ and assumption (d), $K_k$ is a union $\bigcup _{j} K_k^j$ of at most countably many pairwise disjoint connected rectifiable sets $K_k^j$ relatively closed in $U_1.$

Let ${co}(K_k^j)$ denote the closed convex hull of $K_k^j.$ Observe that if $K_k^j$ is not a segment, then the interior of ${co}(K_k^j)$ is non-empty and

4.32 $$\begin{aligned}&\int _{K_k^j\cap (\partial ^*E_k\cup C_k)} \phi (\nu _{K_k})\text {d}\mathcal {H}^1 + 2\int _{K_k^j\cap (((E_{k}^{(1)}\cup E_k^{(0)}) \cap \partial E_k)\cup J_k)} \phi (\nu _{K_k})\text {d}\mathcal {H}^1 \nonumber \\&\quad \geqq \int _{\partial {co}(K_k^j)} \phi (\nu _{{co}(K_k^j)})\text {d}\mathcal {H}^1. \end{aligned}$$

Now we define the minimal union of disjoint closed convex sets containing $K_k$ as follows. For every $k\geqq 1$ let us define the sequences $\{D_i^k\}_i$ of pairwise disjoint subsets of $\mathbb {N}$ and $\{V_i^k\}_i$ of pairwise disjoint closed convex subsets of $\overline{U_1}$ as follows. Let $D_0^k:=\{1\}$ and $V_0^k:=\emptyset .$ Suppose that for some $i\geqq 1$ the sets $D_0^k,\ldots ,D_{i-1}^k$ and $V_0^k,\ldots ,V_{i-1}^k$ are defined and let $j_o$ be the smallest element of $\mathbb {N}{\setminus } \bigcup \limits _{j=0}^{i-1}D_j^k.$ Define

$$\begin{aligned} D_i^k:=\Big \{h\in \mathbb {N}{\setminus } \bigcup \limits _{j=0}^{i-1}D_j^k:\, {co}(K_k^{j_o})\cap {co}(K_k^h)\ne \emptyset \Big \} \end{aligned}$$

and

$$\begin{aligned} V_i^k:={co}(\cup _{h\in D_i^k}\, K_k^h). \end{aligned}$$

Note that $j_o\in D_i^k.$ As in (4.32) we observe that

4.33 $$\begin{aligned}&\sum \limits _{h\in D_i^k} \int _{K_k^h\cap (\partial ^*E_k\cup C_k)} \phi (\nu _{K_k})\text {d}\mathcal {H}^1 + 2\int _{K_k^h\cap (((E_{k}^{(1)}\cup E_k^{(0)}) \cap \partial E_k)\cup J_k)} \phi (\nu _{K_k})\text {d}\mathcal {H}^1 \nonumber \\&\quad \geqq \int _{\partial V_i^k} \phi (\nu _{V_i^k}^{})\text {d}\mathcal {H}^1. \end{aligned}$$

Then $K_k\subset \bigcup \limits _i V_i^k$ and by (4.33)

$$\begin{aligned}&\int _{K\cap (\partial ^*E_k\cup C_k)} \phi (\nu _{K_k})\text {d}\mathcal {H}^1 + 2\int _{K_k\cap (((E_{k}^{(1)}\cup E_k^{(0)}) \cap \partial E_k)\cup J_k)} \phi (\nu _{K_k})\text {d}\mathcal {H}^1\\&\quad \geqq \sum \limits _{i\notin T}\int _{\partial V_i^k} \phi (\nu _{V_i^k}^{})\text {d}\mathcal {H}^1 + 2\sum \limits _{i\in T}\int _{\partial V_i^k} \phi (\nu _{V_i^k}^{})\text {d}\mathcal {H}^1, \end{aligned}$$

where *T* is the set of all indices *i* for which $$V_i^k$$ is a line segment. For every $$i\in T$$ we replace the segment $$V_i^k$$ with a closed rectangle $$Q_i$$ containing $$V_i^k$$ and not intersecting any $$V_j^k,$$$$j\ne i,$$ such that

$$\begin{aligned} 2\int _{V_i^k} \phi (\nu _{V_i^k}^{})\text {d}\mathcal {H}^1\geqq \int _{\partial Q_i} \phi (\nu _{Q_i})\text {d}\mathcal {H}^1 -\frac{\delta }{10\cdot 2^i} \end{aligned}$$

Therefore, redefining $V_i^k:=Q_i$ we obtain

4.34 $$\begin{aligned}&\int _{K_k\cap (\partial ^*E_k\cup C_k)} \phi (\nu _{K_k})\text {d}\mathcal {H}^1 + 2\int _{K_k\cap (((E_{k}^{(1)}\cup E_k^{(0)}) \cap \partial E_k)\cup J_k)} \phi (\nu _{K_k})\text {d}\mathcal {H}^1 \nonumber \\&\quad \geqq \sum \limits _{i}\int _{\partial V_i^k} \phi (\nu _{V_i^k}^{})\text {d}\mathcal {H}^1 -\frac{\delta }{5}. \end{aligned}$$

Note that $U_1{\setminus } \bigcup \limits _i V_i^k$ is a Lipschitz open set and $J_{u_k}\cap (U_1{\setminus } \bigcup \limits _i V_i^k) =\emptyset ,$ and hence, by the Poincaré-Korn inequality, $u_k\in H^1(U_1{\setminus } \bigcup \limits _i V_i^k).$ Moreover, (4.27), (4.9) and (4.34) imply $c_1\sum \limits _i \mathcal {H}^1(\partial V_i^k)<M+1,$ thus, there exists $\eta \in \mathbb {R}^2$ such that the set

$$\begin{aligned} \{x\in U_1\cap \bigcup \limits _i \partial V_i^k:\,\text {trace of } u_k\big |_{U_1{\setminus } \cup V_i^k} \text { is equal to } \eta \} \end{aligned}$$

is $$\mathcal {H}^1$$-negligible (see [46, Proposition 2.6]). Therefore, $$v_k:=u_k\chi _{U_1{\setminus } \cup V_i^k}^{} +\eta \chi _{\cup V_i^k}^{}$$ belongs to $$GSBD^2(U_1;\mathbb {R}^2),$$$$J_{v_k}= U_1\cap \cup _i \partial V_i^k.$$ By assumptions (a), (c) and (h), $$v_k\rightarrow v:=u^+\chi _{U_1^+} +u^-\chi _{U_1{\setminus } U_1^+},$$ and by assumption (g) and inequalities (4.9), and (4.27),

$$\begin{aligned} \sup \limits _k \int _{U_1}|e(v_k)|^2\text {d}x + \mathcal {H}^1(J_{v_k}) \leqq \sup \limits _k \int _{U_1}|e(u_k)|^2\text {d}x + \frac{M+1}{c_1}<\infty . \end{aligned}$$

Repeating the same arguments of the proof of (3.25) we obtain

4.35 $$\begin{aligned} 2\int _{I_1}\phi (\mathbf{e_2})\text {d}\mathcal {H}^1 \leqq \liminf \limits _{k\rightarrow \infty } \int _{J_{v_k}}\phi (\nu _{J_{v_k}})\text {d}\mathcal {H}^1. \end{aligned}$$

Note that the direct application of Proposition 4.6 would not be enough since we would obtain the estimate:

$$\begin{aligned} \int _{I_1}\phi (\mathbf{e_2})\text {d}\mathcal {H}^1 \leqq \liminf \limits _{k\rightarrow \infty } \int _{J_{v_k}}\phi (\nu _{J_{v_k}})\text {d}\mathcal {H}^1 \end{aligned}$$

without coefficient 2 on the left.

From (4.34) and (4.35) it follows that there exists $k_\delta ^1>0$ such that

4.36 $$\begin{aligned}&\int _{U_1\cap ((\Omega _k\cap \partial ^*E_k)\cup C_k)} \phi (\nu _{\partial E_k\cup C_k}^{})\text {d}\mathcal {H}^1 + 2\int _{U_1\cap (((E_{k}^{(1)}\cup E_k^{(0)}) \cap \partial E_k)\cup J_k)} \phi (\nu _{E_k}^{})\text {d}\mathcal {H}^1\nonumber \\&\quad \geqq 2 \int _{I_1}\phi (\mathbf{e_2})\text {d}\mathcal {H}^1 - \frac{2\delta }{5} \end{aligned}$$

for any $k\geqq k_\delta ^1.$ By (4.28) we may suppose that for such *k*, 

$$\begin{aligned} \int _{I_1}\phi (\mathbf{e_2})\text {d}\mathcal {H}^1 \geqq \int _{U_1\cap \Sigma _k} \phi (\nu _{\Sigma _k})\text {d}\mathcal {H}^1 -\frac{\delta }{5}. \end{aligned}$$

Thus, in view of (4.31), from (4.36) we get (4.30).

*Step 2.* Finally we prove (4.26). Let $k_{\delta /2}^1$ be given by Step 1 with $\delta /2$ in place of $\delta .$ From (4.29) for $k>k_{\delta /2}^1$ we have

4.37 $$\begin{aligned}&\int _{U_1\cap \Omega _k \cap \partial ^*E_k} \phi (\nu _{E_k})\text {d}\mathcal {H}^1 +2\int _{U_1\cap \Omega _k \cap (E_{k}^{(1)}\cup E_k^{(0)}) \cap \partial E_k} \phi (\nu _{E_k})\text {d}\mathcal {H}^1\nonumber \\&\qquad + \int _{U_1\cap \Sigma _k{\setminus } \partial E_k} g_0 \text {d}\mathcal {H}^1 + \int _{U_1\cap \Sigma _k\cap E_k^{(0)}\cap \partial E_k} \big (\phi (\nu _{\Sigma _k}) + g_1\big )\text {d}\mathcal {H}^1\nonumber \\&\qquad + \int _{U_1\cap \Sigma _k\cap \partial ^*E_k{\setminus } J_{E_k}} g_1\text {d}\mathcal {H}^1 + \int _{U_1\cap J_{E_k}} \big (\phi (\nu _{\Sigma _k}) + g_0 \big )\,\text {d}\mathcal {H}^1\nonumber \\&\quad \geqq \int _{U_1\cap \Sigma _k{\setminus } \partial E_k} \big (\phi (\nu _{\Sigma _k}) +g_0\big ) \text {d}\mathcal {H}^1 + \int _{U_1\cap \Sigma _k\cap E_k^{(0)}\cap \partial E_k} \big (2\phi (\nu _{\Sigma _k}) + g_1\big )\text {d}\mathcal {H}^1\nonumber \\&\qquad + \int _{U_1\cap \Sigma _k\cap \partial ^*E_k{\setminus } J_{E_k}} \big (2\phi (\nu _{\Sigma _k})+g_1\big )\text {d}\mathcal {H}^1 + \int _{U_1\cap J_{E_k}} \big (\phi (\nu _{\Sigma _k}) + g_0 \big )\,\text {d}\mathcal {H}^1- \frac{\delta }{2}. \end{aligned}$$

Thus, (4.28) implies that there exists $k_\delta >k_{\delta /2}^1$ such that

$$\begin{aligned}&\int _{U_1\cap \Sigma _k{\setminus } \partial E_k} \big (\phi (\nu _{\Sigma _k}) +g_0\big ) \text {d}\mathcal {H}^1 + \int _{U_1\cap \Sigma _k\cap E_k^{(0)}\cap \partial E_k} \big (2\phi (\nu _{\Sigma _k}) + g_1\big )\text {d}\mathcal {H}^1\nonumber \\&\qquad + \int _{U_1\cap \Sigma _k\cap \partial ^*E_k{\setminus } J_{E_k}} \big (2\phi (\nu _{\Sigma _k})+g_1\big )\text {d}\mathcal {H}^1 + \int _{U_1\cap J_{E_k}} \big (\phi (\nu _{\Sigma _k}) + g_0 \big )\,\text {d}\mathcal {H}^1\\&\quad \geqq \int _{I_1\cap \pi (\Sigma _k{\setminus } \partial E_k)} \big (\phi (\mathbf{e_2}) +g_0\big ) \text {d}\mathcal {H}^1 + \int _{I_1\cap \pi (\Sigma _k\cap E_k^{(0)}\cap \partial E_k)} \big (2\phi (\mathbf{e_2}) + g_1\big )\text {d}\mathcal {H}^1\nonumber \\&\qquad + \int _{I_1\cap \pi (\Sigma _k\cap \partial ^*E_k{\setminus } J_{E_k})} \big (2\phi (\mathbf{e_2})+g_1\big )\text {d}\mathcal {H}^1 + \int _{I_1\cap \pi (J_{E_k})} \big (\phi (\mathbf{e_2}) + g_0 \big )\,\text {d}\mathcal {H}^1-\frac{\delta }{2}\\&\quad \geqq \int _{I_1} \big (\phi (\mathbf{e_2}) +g_0\big ) \text {d}\mathcal {H}^1 -\frac{\delta }{2}, \end{aligned}$$

where we used also that $g_0$ and $g_1$ are constants. Now (4.26) follows from (4.37) and the last inequality. $\quad \square$

### Proof of Proposition 4.1

Without loss of generality, we suppose that the limit in the left-hand side of (4.3) is reached and finite. Define

$$\begin{aligned} g_+(x,s) = g(x,s) - g(x,0) + \varphi (x,\nu _\Sigma (x)). \end{aligned}$$

Then $g_+$ is Borel, $g_+(\cdot ,s)\in L^1(\Sigma )$ for $s=0,1,$ and by (4.1), $g_+\geqq 0$ and

4.38 $$\begin{aligned} |g_+(x,1) - g_+(x,0)| \leqq \varphi (x,\nu _\Omega (x)) \end{aligned}$$

for $\mathcal {H}^1$-a.e. $x\in \Sigma .$ Consider the sequence $\mu _k$ of Radon measures in $\mathbb {R}^2,$ associated to $\mathcal {S}(A_k,J_{A_k};\varphi ,g),$ defined at Borel sets $B\subset \mathbb {R}^2$ by

$$\begin{aligned} \mu _k(B)&:= \int _{B\cap \Omega \cap \partial ^*A_k} \varphi (x,\nu _{A_k})\text {d}\mathcal {H}^1 +2\int _{B\cap \Omega \cap (A_k^{(1)}\cup A_k^{(0)})\cap \partial A_k} \varphi (x,\nu _{A_k})\text {d}\mathcal {H}^1\nonumber \\&\quad + \int _{B\cap \Sigma {\setminus } \partial A_k} g_+(x,0)\text {d}\mathcal {H}^1 + \int _{B\cap \Sigma \cap A_k^{(0)}\cap \partial A_k} \big (\varphi (x,\nu _\Sigma ) + g_+(x,1)\big )\text {d}\mathcal {H}^1\nonumber \\&\quad + \int _{B\cap \Sigma \cap \partial ^*A_k{\setminus } J_{A_k}} g_+(x,1)\text {d}\mathcal {H}^1 + \int _{B\cap J_{A_k}} \big (\varphi (x,\nu _\Sigma ) + g_+(x,0) \big )\,\text {d}\mathcal {H}^1. \end{aligned}$$

Analogously, we define the positive Radon measure $\mu$ in $\mathbb {R}^2$ associated to $\mathcal {S}(A,J_A;\varphi ,g),$ writing *A* in place of $A_k$ in the definition of $\mu _k.$ By (2.4), assumption $A_k\overset{\tau _\mathcal {A}}{\rightarrow } A$ and the nonnegativity of $g_+,$

$$\begin{aligned} \sup \limits _{k\geqq 1} \mu _k(\mathbb {R}^2) \leqq 2c_2 \sup \limits _{k\geqq 1} \mathcal {H}^1(\partial A_k) + \sum \limits _{s=0}^1 \int _\Sigma g_+(x,s)\text {d}\mathcal {H}^1<\infty . \end{aligned}$$

Thus, by compactness there exists a (not relabelled) subsequence $\{\mu _k\}$ and a non-negative bounded Radon measure $\mu _0$ in $\mathbb {R}^2$ such that $\mu _k\overset{*}{\rightharpoonup }\mu _0$ as $k\rightarrow \infty .$ We claim that

4.39 $$\begin{aligned} \mu _0\geqq \mu , \end{aligned}$$

which implies the assertion of the proposition. In fact, (4.3) follows from (4.39), the weak*-convergence of $\mu _k,$ and the equalities

$$\begin{aligned} \mu _k(\mathbb {R}^2) = \mathcal {S}(A_k,J_{A_k};\varphi ,g) + \int _{\Sigma } \big (\varphi (x,\nu _\Sigma ) - g(x,0)\big )\text {d}\mathcal {H}^1 \end{aligned}$$

and

$$\begin{aligned} \mu (\mathbb {R}^2) = \mathcal {S}(A,J_{A};\varphi ,g) + \int _{\Sigma } \big (\varphi (x,\nu _\Sigma ) - g(x,0)\big )\text {d}\mathcal {H}^1. \end{aligned}$$

Since $\mu _0$ and $\mu$ are non-negative, and  by Remark 2.3 to prove (4.39) it suffices to establish the following lower-bound estimates for densities of $\mu _0$ with respect to $\mathcal {H}^1$ restricted to various parts of $\partial A:$4.40a4.40b4.40c4.40d4.40e4.40f4.40g

We separately outline below the proofs of (4.40a)–(4.40g).

*Proof of* (4.40a). Consider points $x\in \Omega \cap \partial ^*A$ such that

1. $$\nu _A(x)$$ exists;
2. *x* is a Lebesgue point of $y\in \partial ^*A \mapsto \varphi (y,\nu _A(y)),$ i.e.,
  $$\begin{aligned} \lim \limits _{r\rightarrow 0} \frac{1}{2r}\int _{U_r\cap \partial ^*A} |\varphi (y,\nu _A(y))- \varphi (x,\nu _A(x))|\text {d}\mathcal {H}^1(y)=0; \end{aligned}$$
exists and is finite.

By the definition of $\partial ^*A,$ continuity of $\phi ,$ the Borel regularity of $y\in \partial ^*A \mapsto \varphi (y,\nu _A(y)),$ and the Besicovitch Derivation Theorem, the set of points $x\in \Omega \cap \partial ^*A$ not satisfying these conditions is $\mathcal {H}^1$-negligible, hence we prove (4.40a) for $x\in \Omega \cap \partial ^*A$ satisfying (a1)–(a3). Without loss of generality we suppose $x=0$ and $\nu _A(x) = \mathbf{e_2}.$ By Lemma 3.2, $A_k\rightarrow A$ in $L^1(\mathbb {R}^2),$ therefore, $D\chi _{A_k} \overset{*}{\rightharpoonup }D\chi _A,$ and hence, by the Besicovitch Derivation Theorem [3, Theorem 2.22] and the definition (2.1) of the reduced boundary,Then for a.e. $r>0$ such that $U_r\subset \subset \Omega$ and $\mathcal {H}^1(\partial U_r\cap \partial A)=0,$ the Reshetnyak Lower-semicontinuity Theorem [3, Theorem 2.38] implies

$$\begin{aligned} \mu _0(U_r)&= \liminf \limits _{k\rightarrow \infty } \mu _k(U_r) \geqq \liminf \limits _{k\rightarrow \infty } \int _{U_r\cap \partial ^*A_k} \varphi (y,\nu _{A_k})\,\text {d}\mathcal {H}^1 \geqq \int _{U_r\cap \partial ^*A} \varphi (y,\nu _A)\,\text {d}\mathcal {H}^1 \end{aligned}$$

Therefore, by [32, Theorem 1.153] and assumption (a2),*Proof of* (4.40b). Consider points $x\in \Omega \cap A^{(0)}\cap \partial A$ such that

1. $$\theta ^*(\partial A,x) = \theta _*(\partial A,x)=1;$$
2. $$\nu _A(x)$$ exists;
3. $\overline{U_1}\cap \sigma _{\rho ,x}(\partial A)\overset{\mathcal {K}}{\rightarrow } \overline{U_1}\cap T_x,$ where $T_x$ is the approximate tangent line to $\partial A$ and $\sigma _{\rho ,x}$ is given by (4.4);
exists and finite.

By the $\mathcal {H}^1$-rectifiability of $\partial A,$ Proposition A.4 (applied with the closed connected component *K* of $\partial A$ containing *x*) and the Besicovitch Derivation Theorem, the set of points $x\in A^{(0)}\cap \partial A$ not satisfying these conditions is $\mathcal {H}^1$-negligible, hence we prove (4.40b) for $x\in A^{(0)}\cap \partial A$ satisfying (b1)–(b4). Without loss of generality we assume $x=0$, $\nu _A(x) = \mathbf{e_2}$ and $T_x = T_0$ is the $x_1$-axis.

Let us choose a sequence $\rho _n\searrow 0$ such that

4.41 $$\begin{aligned} \mu _0(\partial U_{\rho _n})=0\qquad \text {and}\qquad \lim \limits _{k\rightarrow \infty } \mu _k(\overline{U_{\rho _n}}) = \mu _0(U_{\rho _n}) \end{aligned}$$

and4.42By Proposition A.5 (a), (b2) and (b3) imply that $$\mathrm {sdist}(\cdot ,\sigma _{\rho _n}(\partial A))\rightarrow \mathrm {dist}(\cdot ,T_0)$$ uniformly in $$\overline{U_1}.$$ Since for any $$n>1,$$$$\mathrm {sdist}(\cdot ,\partial A_k)\rightarrow \mathrm {sdist}(\cdot ,\partial A)$$ uniformly in $$\overline{U_{\rho _n}}$$ as $$k\rightarrow \infty ,$$ by a diagonal argument, we find a subsequence $$\{A_{k_n}\}$$ such that

$$\begin{aligned} \mathrm {sdist}(\cdot ,\sigma _{\rho _n} (\partial A_{k_n}))\rightarrow \mathrm {dist}(\cdot ,T_0)\quad \hbox { uniformly in}\ \overline{U_1}, \end{aligned}$$

as $$n\rightarrow \infty $$ and

4.43 $$\begin{aligned} \mu _{k_n}(\overline{U_{\rho _n}}) \leqq \mu _0(U_{\rho _n}) + \rho _n^2 \end{aligned}$$

for any *n*. By Lemma 4.2, $\overline{U_1}\cap \sigma _{\rho _n}(A_{k_n}) \overset{\mathcal {K}}{\rightarrow }I_1:=\overline{U_1}\cap T_0.$

From (2.4), (4.43), the definition of $\mu _k,$ (4.42) and (b4) it follows that4.44By the uniform continuity of $\varphi ,$ for every $\epsilon >0$ there exists $n_\epsilon >0$ such that

4.45 $$\begin{aligned} \varphi (y,\xi ) \geqq \varphi (0,\xi ) - \epsilon \end{aligned}$$

for every $$y\in U_{\rho _{n_\epsilon }}.$$ Moreover, since $$\{A_k\}\subset \mathcal {A}_m,$$ the number of connected components of $$\partial \sigma _{\rho _n}(A_{k_n})$$ lying strictly inside $$U_1,$$ does not exceed from *m*. Hence, applying Lemma 4.4 with $$\phi =\varphi (0,\cdot ),$$$$m_o=m$$ and $$\delta =\epsilon ,$$ we find $$n_\epsilon '>n_\epsilon $$ such that for any $$n>n_\epsilon ',$$

$$\begin{aligned}&\int _{U_1\cap \partial ^*\sigma _{\rho _n}(A_{k_n})} \varphi (0,\nu _{\sigma _{\rho _n}(A_{k_n}) })\,\text {d}\mathcal {H}^1\\&\qquad + 2\int _{U_1\cap \big ((\sigma _{\rho _n}(A_{k_n}))^{(0)}\cup (\sigma _{\rho _n}(A_{k_n}))^{(1)}\big )\cap \partial \sigma _{\rho _n}(A_{k_n})} \varphi (0,\nu _{\sigma _{\rho _n}(A_{k_n}) })\,\text {d}\mathcal {H}^1\\&\quad \geqq 2 \int _{I_1} \varphi (0,\mathbf{e_2})\,\text {d}\mathcal {H}^1-\epsilon = 4\varphi (0,\mathbf{e_2})-\epsilon . \end{aligned}$$

Therefore, by the definition of $\mu _k,$ for such *n* one has

4.46 $$\begin{aligned} \mu _{k_n}(\overline{U_{\rho _n}})&\geqq \int _{\overline{U_{\rho _n}}\cap \partial ^* A_{k_n}} \varphi (y,\nu _{A_{k_n}})\,\text {d}\mathcal {H}^1 + 2\int _{\overline{U_{\rho _n}}\cap \big (A_k^{(0)}\cup A_k^{(1)}\big )\cap \partial A_{k_n}} \varphi (y,\nu _{A_{k_n}})\,\text {d}\mathcal {H}^1\nonumber \\&\geqq \int _{\overline{U_{\rho _n}}\cap \partial ^* A_{k_n}} \varphi (0,\nu _{A_{k_n}})\,\text {d}\mathcal {H}^1 + 2\int _{\overline{U_{\rho _n}}\cap \big (A_k^{(0)}\cup A_k^{(1)}\big )\cap \partial A_{k_n}} \varphi (0,\nu _{A_{k_n}})\,\text {d}\mathcal {H}^1\nonumber \\&\quad - \epsilon \,\mathcal {H}^1(\overline{U_{\rho _n}}\cap \partial A_k)\nonumber \\&= \rho _n \Big (\int _{U_1\cap \partial ^*\sigma _{\rho _n}(A_{k_n})} \varphi (0,\nu _{\sigma _{\rho _n}(A_{k_n}) })\,\text {d}\mathcal {H}^1\nonumber \\&\quad + 2\int _{U_1\cap \big ((\sigma _{\rho _n}(A_{k_n}))^{(0)}\cup (\sigma _{\rho _n}(A_{k_n}))^{(1)}\big )\cap \partial \sigma _{\rho _n}(A_{k_n})} \varphi (0,\nu _{\sigma _{\rho _n}(A_{k_n}) })\,\text {d}\mathcal {H}^1\Big )\nonumber \\&\quad - \epsilon \,\mathcal {H}^1(\overline{U_{\rho _n}}\cap \partial A_{k_n}) \nonumber \\&\geqq 4\rho _n\varphi (0,\mathbf{e_2}) -\epsilon \rho _n - \epsilon \,\mathcal {H}^1(\overline{U_{\rho _n}}\cap \partial A_{k_n}) , \end{aligned}$$

and thus, by (4.42)–(4.44),Now using assumption (b4) and letting $\epsilon \rightarrow 0^+$ we obtain (4.40b).

*Proof of* (4.40c). We repeat the same arguments of the proof of (4.40b) using Lemma 4.5 in place of Lemma 4.4 and Proposition A.5 (a) in place of Proposition A.5 (b).

*Proof of* (4.40d). Given $x\in \Sigma {\setminus } \partial A,$ there exists $r_x>0$ such that $B_{r_x}(x)\cap \partial A = \emptyset .$ Since $\partial A_k\overset{\mathcal {K}}{\rightarrow }\partial A,$ there exists $k_x$ such that $B_{r_x/2}(x)\cap \partial A_k = \emptyset$ for all $k>k_x.$ Thus, for any $r\in (0,r_x/2),$

$$\begin{aligned} \mu _k(B_r(x)) = \int _{\Sigma \cap B_r(x)} g_+(y,0)\text {d}\mathcal {H}^1 \end{aligned}$$

so thatfor $\mathcal {H}^1$-a.e. Lebesgue points $x\in \Sigma {\setminus } \partial A$ of $g_+.$

*Proof of* (4.40e). Consider points $x\in \Sigma \cap A^{(0)}\cap \partial A$ such that

1. $$\theta ^*(\Sigma \cap \partial A,x) = \theta _*(\Sigma \cap \partial A,x)=1;$$
2. $\nu _\Sigma (x)$ and $\nu _A(x)$ exist (clearly, either $\nu _\Sigma (x)=\nu _A(x)$ or $\nu _\Sigma (x) = -\nu _A(x)$);
3. $\overline{U_1}\cap \sigma _{\rho ,x}(\partial A)\overset{\mathcal {K}}{\rightarrow } \overline{U_1}\cap T_x,$ where $T_x$ is the approximate tangent line to $\partial A;$
4. *x* is a Lebesgue point of $g_+(\cdot ,1),$ i.e.,
  $$\begin{aligned} \lim \limits _{\rho \rightarrow 0} \,\frac{1}{2\rho }\int _{\Sigma \cap U_{\rho ,\nu _\Sigma (x)}} |g_+(y,1) - g_+(x,1)|\text {d}\mathcal {H}^1(y) = 0; \end{aligned}$$
5. *x* is a Lebesgue point of $y\in \Sigma \cap \varphi (y,\nu _\Sigma (y)),$ i.e.,
  $$\begin{aligned} \lim \limits _{\rho \rightarrow 0} \,\frac{1}{2\rho }\int _{\Sigma \cap U_{\rho ,\nu _\Sigma (x)}} |\varphi (y,\nu _\Sigma (y)) - \varphi (x,\nu _\Sigma (x))|\text {d}\mathcal {H}^1(y) = 0; \end{aligned}$$
exists and is finite.

By the $$\mathcal {H}^1$$-rectifiability of $$\partial A,$$ the Lipschitz continuity of $$\Sigma $$, the Borel regularity of $$\nu _\Sigma (\cdot ),$$ Proposition A.4 (applied with closed connected component *K* of $$\partial A$$ containing *x*), the continuity of $$\varphi $$, assumptions on $$g_+$$ and the Besicovitch Derivation Theorem, the set of $$x\in \Sigma \cap \partial A$$ not satisfying these conditions is $$\mathcal {H}^1$$-negligible. Hence, we prove (4.40e) for *x* satisfying (e1)–(e6). Without loss of generality we assume $$x=0,$$$$\nu _\Sigma (x) = \nu _A(x) = \mathbf{e_2}$$ and $$T_x=T_0$$ is the $$x_1$$-axis. Let $$r_n\searrow 0$$ be such that

$$\begin{aligned} \mu _0(\partial U_{r_n}) = \mathcal {H}^1(\partial U_{r_n}\cap \Sigma ) =0 \end{aligned}$$

and4.47By the weak*-convergence, for any $h\geqq 1$ we have

$$\begin{aligned} \lim \limits _{k\rightarrow \infty } \mu _k(\overline{U_{r_n}}) = \mu _0(U_{r_n}). \end{aligned}$$

By Proposition A.5 (b), (e2) and (e3) imply $\mathrm {sdist}(\cdot , \sigma _{r_n}(\partial A)) \rightarrow \mathrm {dist}(\cdot , T_0)$ uniformly in $\overline{U_1}.$ Since for any *n*,  $\mathrm {sdist}(\cdot ,\sigma _{r_n}(\partial A_k)) \rightarrow \mathrm {sdist}(\cdot ,\sigma _{r_n}(\partial A))$ uniformly in $\overline{U_1}$ as $k\rightarrow \infty ,$ by a diagonal argument, we can find a subsequence $\{k_n\}$ and not relabelled subsequence $\{r_n\}$ such that

4.48 $$\begin{aligned} \mu _{k_n}(\overline{U_{r_n}}) \leqq \mu _0(U_{r_n}) + r_n^2 \end{aligned}$$

for any $n\geqq 1$ and $\mathrm {sdist}(\cdot ,\sigma _{r_n}(A_k)) \rightarrow \sigma (\cdot ,T_0)$ uniformly in $\overline{U_1}$ as $k\rightarrow \infty ,$ thus, by Lemma 4.2,

4.49 $$\begin{aligned} U_1 \cap \sigma _{r_n}(A_{k_n}) \overset{\mathcal {K}}{\rightarrow }I_1:=U_1\cap T_0 \end{aligned}$$

as $n\rightarrow \infty .$ Notice also that by (e2) and Proposition A.4 (applied with the closed connected component *K* of $\Sigma$), $U_1\cap \sigma _{r_n}(\Sigma )\overset{\mathcal {K}}{\rightarrow } I_1$ as $n\rightarrow \infty .$

By (4.38),

$$\begin{aligned} \varphi (y,\nu _\Sigma (x))+g_+(y,0)\geqq g_+(y,1) \end{aligned}$$

for $\mathcal {H}^1$-a.e. on $\Sigma ,$ in particular on $J_{A_k},$ hence, by Remark 2.3 and the definition of $\mu _k,$

$$\begin{aligned} \mu _{k_n}(\overline{U_{r_n}})&\geqq \int _{\overline{U_{r_n}} \cap \Omega \cap \partial ^*A_{k_n}} \varphi (y,\nu _{A_{k_n}})\text {d}\mathcal {H}^1 +2\int _{\overline{U_{r_n}} \cap \Omega \cap \big (A_{k_n}^{(0)} \cup A_{k_n}^{(1)}\big )\cap \partial A_{k_n}} \varphi (y,\nu _{A_{k_n}})\text {d}\mathcal {H}^1\\&\quad + \int _{U_{r_n}\cap \Sigma } g_+(y,1)\text {d}\mathcal {H}^1 + \int _{U_{r_n} \cap \Sigma \cap A_{k_n}^{(0)}\cap \partial A_{k_n}} \varphi (y,\nu _\Sigma )\text {d}\mathcal {H}^1\\&\quad +\int _{U_{r_n} \cap \Sigma {\setminus } \partial A_{k_n}} \big (g_+(y,0) - g_+(y,1)\big )\text {d}\mathcal {H}^1 \end{aligned}$$

Adding and subtracting $\int _{\overline{U_{r_n}} \cap \Sigma \cap \partial ^* A_{k_n}} \phi (y,\nu _{A_{k_n}})\text {d}\mathcal {H}^1$ to the right and using (4.38) once more in the integral over $U_{r_n} \cap \Sigma {\setminus } \partial A_{k_n}$ we get

4.50 $$\begin{aligned} \mu _{k_n}(\overline{U_{r_n}})&\geqq \int _{\overline{U_{r_n}} \cap \partial ^*A_{k_n}} \varphi (y,\nu _{A_{k_n}})\text {d}\mathcal {H}^1 +2\int _{\overline{U_{r_n}} \cap \big ( A_{k_n}^{(0)}\cup A_{k_n}^{(1)}\big ) \cap \partial A_{k_n}} \varphi (y,\nu _{A_{k_n}})\text {d}\mathcal {H}^1\nonumber \\&\quad + \int _{U_{r_n}\cap \Sigma } g_{ + }(y,1)\text {d}\mathcal {H}^1 - \int _{U_{r_n} \cap \Sigma } \varphi (y,\nu _\Sigma (y))\text {d}\mathcal {H}^1 \end{aligned}$$

By the uniform continuity of $\varphi ,$ given $\epsilon \in (0,1)$ there exists $n_\epsilon >0$ such that

$$\begin{aligned} |\varphi (y,\nu ) - \varphi (0,\nu )| < \epsilon \end{aligned}$$

for all $$y\in U_{r_n},$$$$\nu \in \mathbb {S}^1$$ and $$n>n_\epsilon .$$ We suppose also that Lemma 4.4 holds with $$n_\epsilon $$ when $$\delta =\epsilon .$$ Since the number of connected components of $$\partial A_{k_n}$$ lying strictly inside $$U_{r_n}$$ is not greater than *m*,  in view of (4.49) and the non-negativity of $$g_+,$$ as in (4.46) for all $$n>n_\epsilon $$ we obtain

4.51 $$\begin{aligned} \mu _{k_n}(\overline{U_{r_n}})&\geqq 4r_n \varphi (0,\mathbf{e_2}) - \epsilon r_n - \epsilon \mathcal {H}^1(\overline{U_{r_n}\cap \partial A_{k_n}})\nonumber \\&\quad + \int _{U_{r_n}\cap \Sigma } g_{ + }(y,1)\text {d}\mathcal {H}^1 - \int _{U_{r_n} \cap \Sigma } \varphi (y,\nu _\Sigma (y))\text {d}\mathcal {H}^1. \end{aligned}$$

By the non-negativity of $g_+,$ (2.4) and (4.48),

$$\begin{aligned} \mathcal {H}^1(\overline{U_{r_n}\cap \partial A_{k_n}})&\leqq \mathcal {H}^1(U_{r_n}\cap \Omega \cap \cap \partial A_{k_n}) + \mathcal {H}^1(U_{r_n}\cap \Sigma \cap \cap \partial A_{k_n})\\&\leqq \frac{\mu _{k_n}(U_{r_n})}{c_1} + \mathcal {H}^1(U_{r_n}\cap \Sigma )\leqq \mu _0(U_{r_n}) +r_n^2 +\mathcal {H}^1(U_{r_n}\cap \Sigma ) \end{aligned}$$

thus again using (4.48), also (4.47), (4.50) and (4.51), as well as (e1) and (e3)–(e5) we establishNow letting $\epsilon \rightarrow 0$ and using $\nu _\Sigma (0)=\mathbf{e_2}$ we obtain (4.40e).

*Proof of* (4.40f). Since $$g_+$$ is non-negative and $$\chi _{A_k}\rightarrow \chi _A$$ in $$L^1(\mathbb {R}^2),$$ the inequality follows from [1, Lemma 3.8] applied to $$u_k=\chi _{A_k},$$$$u=\chi _A$$ and

$$\begin{aligned} \mathcal {F}_U(v,B) := \int _{U\cap B\cap J_v{\setminus } \Sigma } \varphi (x,\nu _{J_v})\text {d}\mathcal {H}^1 + \int _{\Sigma \cap B} g_+(x,1)v^+(x)\,\text {d}\mathcal {H}^1, \end{aligned}$$

where $U:=\Omega$. More precisely, by (4.1),

4.52 $$\begin{aligned} \mu _k(B) \geqq \mathcal {F}_U(\chi _{A_k},B), \end{aligned}$$

so that the total variation of $\mathcal {F}_U(\chi _{A_k},\cdot )$ is uniformly bounded. Therefore, passing to a further not relabelled subsequence if necessary, we have $\mathcal {F}_U(\chi _{A_k},\cdot ) \rightharpoonup ^* {\bar{\mu }}$ as $k\rightarrow \infty$ for some bounded positive Radon measure ${\bar{\mu }}$ in $\mathbb {R}^2.$ By [1, Lemma 3.8, Eq. 3.15],By (4.52), $\mu _0\geqq {\bar{\mu }},$ and thus, (4.40f) follows.

*Proof of* (4.40g). Consider points $x\in J_A$ for which

1. $$\theta ^*(J_A,x) = \theta _*(J_A,x) = \theta ^*(\partial A,x) = \theta _*(\partial A,x) =\theta ^*(\Sigma ,x) = \theta _*(\Sigma ,x) =1;$$
2. assumption (b) of Proposition 4.1 holds with some $r=r_x>0$;
3. $\Sigma$ is differentiable at *x* and $\nu _\Sigma (x)$ exists;
4. one-sided traces $w^+(x)\ne w^-(x)$ of *w*,  given by assumption (b) of Proposition 4.1, exists;
5. *x* is a Lebesgue point of $g_+(\cdot ,s)$ and $|g_+(x,0) - g_+(x,1)|\leqq \phi (\nu _\Sigma (x));$
exists and finite.

By the $$\mathcal {H}^1$$-rectifiability of $$J_A,$$$$\partial A$$ and $$\Sigma ,$$ assumption (b) of Proposition 4.1 (recall that $$J_A\subset J_w$$), the definition of the jump set of *GSBD*-functions, (4.38), and the Besicovitch Derivation Theorem, the set of points $$x\in J_A$$ not satisfying these conditions is $$\mathcal {H}^1$$-negligible. Hence we prove (4.40g) for $$x\in J_A$$ satisfying (g1)–(g6). Without loss of generality, we assume $$x=0$$ and $$\nu _\Sigma (x) = \mathbf{e_2}.$$ Let $$r_0=r_x$$ and $$w_k\in GSBD^2(B_{r_0}(0);\mathbb {R}^2)$$ be given by assumption (b) of Proposition 4.1. Note that by the weak*-convergence of $$\mu _k,$$

4.53 $$\begin{aligned} \lim \limits _{k\rightarrow \infty } \mu _k(\overline{U_r}) = \mu _0(U_r). \end{aligned}$$

for a.e. $r\in (0,r_0),$ and by (g1), (g3), and Proposition A.4 (applied with connected components of $\Sigma$ and $\partial A$ intersecting at *x*) and also by the definition of blow-up,4.54and4.55as $r\rightarrow 0.$ Since $J_A\subset \Sigma ,$ in view of (4.54),

4.56 $$\begin{aligned} U_4 \cap \sigma _{r}(J_A)\overset{\mathcal {K}}{\rightarrow } I_4. \end{aligned}$$

Moreover, since $J_A$ has a generalized normal at $x=0,$4.57as $r\rightarrow 0.$ In particular, from (4.54) and (4.55),

4.58 $$\begin{aligned} \mathrm {sdist}(\cdot , U_4 \cap \sigma _r(\partial \Omega ))\rightarrow \mathrm {sdist}(\cdot ,\partial U_4^+) \end{aligned}$$

and

4.59 $$\begin{aligned} \mathrm {sdist}(\cdot , U_4 \cap \sigma _r(\partial A))\rightarrow \mathrm {sdist}(\cdot ,\partial U_4^+) \end{aligned}$$

locally uniformly in $U_{3/2}$ as $r\rightarrow 0.$

Letting $\phi =\varphi (0,\cdot ),$*we claim* that there exist sequences $r_h\searrow 0$ and $k_h\nearrow \infty$ such that the sets

$$\begin{aligned} \Omega _h:= U_4 \cap \sigma _{r_h}(\Omega ),\quad \Sigma _h:= U_4 \cap \sigma _{r_h}(\Sigma ),\quad \end{aligned}$$

and

$$\begin{aligned} E_h:= U_4 \cap \sigma _{r_h}(A_{k_h}),\qquad J_{E_h} = U_4 \cap \sigma _{r_h}(J_{A_{k_h}}), \end{aligned}$$

the functions $u_h(x) = w_{k_h}(r_hx)\in GSBD^2( U_4; \mathbb {R}^2),$ the numbers $g_s = g_+(0,s)\in [0,+\infty )$ and the vectors $u^\pm =w^\pm (0)$ satisfy assumptions (a)–(h) of Lemma 4.7.

Indeed, let $\tau$ be any homeomorphism between $\mathbb {R}^2$ and a bounded subset of $\mathbb {R}^2;$ for example, one can take $\tau (x_1,x_2) = (\tanh (x_1),\tanh (x_2)).$ By (4.2), $w_k(rx)\rightarrow w(rx)$ as $k\rightarrow \infty$ for a.e. $x\in U_4$ and for any $r\in (0,r_0/4),$ so that by the Dominated Convergence Theorem,

4.60 $$\begin{aligned} \lim \limits _{k\rightarrow \infty } \int _{U_1} |\tau (w_k(rx)) - \tau (w(rx))|\text {d}x=0. \end{aligned}$$

Moreover, by (g4), the definition [19, Definition 2.4] of the (approximate) jump of the function *w* and [19, Remark 2.2],

4.61 $$\begin{aligned} \lim \limits _{r\rightarrow 0}\int _{U_1} |\tau (w(rx)) - (\tau (u(x)) |\text {d}x=0, \end{aligned}$$

where $u(x):=w^+(0)\chi _{U_1^+}(x) + w^-(0)\chi _{U_1 {\setminus } U_1^+}(x).$ We use (4.60) and (4.61) to extract sequences $k_h\rightarrow \infty$ and $r_h\rightarrow 0$ such that $w_{k_h}(r_hx) \rightarrow u(x)$ a.e. in $U_1.$ By assumption $A_k\overset{\tau _\mathcal {A}}{\rightarrow }A$ and the relations (4.59), (4.53), (4.61) and (4.60), there exist $k_h^1>1$ and a decreasing sequence $r_h\in (0,\frac{1}{h})$ such that for any $h>1$ and $k>k_h^1,$

4.62a $$\begin{aligned}&\Vert \mathrm {sdist}(\cdot ,\sigma _{r_h}( U_{4r_h} \cap \partial A_k)) - \mathrm {sdist}(\cdot ,\sigma _{r_h}( U_{4r_h} \cap \partial A)) \Vert _{L^\infty ( U_{3/2})}^{} < \frac{1}{h} \end{aligned}$$

4.62b $$\begin{aligned}&\Vert \mathrm {sdist}(\cdot ,\sigma _{r_h}( U_{4r_h} \cap \partial A)) - \mathrm {sdist}(\cdot ,\partial U_4^+ ) \Vert _{L^\infty (U_{3/2})}^{} < \frac{1}{h} \end{aligned}$$

4.62c $$\begin{aligned}&\mu _k(\overline{U_{r_h}}) < \mu _0(U_{r_h}) +r_h^2 \end{aligned}$$

4.62d $$\begin{aligned}&\int _{U_1} |\tau (w(r_hx)) - \tau (u(x)) |\text {d}x <\frac{1}{h} \end{aligned}$$

4.62e $$\begin{aligned}&\int _{U_1} |\tau (w_k(r_hx)) - \tau (w(r_hx)) |\text {d}x <\frac{1}{h}. \end{aligned}$$

For every $h\geqq 1,$ we choose $k_h>k_h^1$ such that

4.63 $$\begin{aligned} \frac{1}{k_hr_h} <\frac{1}{h}. \end{aligned}$$

Now $$u_h(x): = w_{k_h}(r_hx)\in GSBD^2(U_2;\mathbb {R}^2),$$ and

- by (4.58), $\mathrm {sdist}(\cdot , U_4\cap \partial \Omega _h)\rightarrow \mathrm {sdist}(\cdot ,\partial U_4^+)$ locally uniformly in $U_{3/2}$ as $h\rightarrow \infty ;$
- by assumption (g3) and the Lipschitz property of $$\Sigma ,$$$$U_{4r_h}\cap \Sigma $$ is a graph of a *L*-Lipschitz function $$l:[-4r_h,4r_h]\rightarrow \mathbb {R}$$ so that $$\Sigma _h= U_4 \cap \partial \Omega _h$$ is the graph of $$l_h(t):=l(r_ht),$$ where $$t\in [-4,4],$$ so that $$l_h(0)=0$$ and $$|l_h'|\leqq \frac{L}{h}$$ by choice (4.63) of $$r_h;$$
- by (4.62a) and (4.62b), $\mathrm {sdist}(\cdot ,U_4\cap \partial E_h)\rightarrow \mathrm {sdist}(\cdot ,\partial U_4^+)$ as $h\rightarrow +\infty ;$
- by assumption $A_k\in \mathcal {A}_m,$ the number of connected components of $\partial E_h$ lying strictly inside $U_4$ does not exceed *m*; 
- by (g5), $$|g_1-g_0|\leqq \phi (\mathbf{e_2});$$
- by (4.2), $J_{u_h}\subset (\Omega _h \cap \partial E_h) \cup J_{E_h}\cup {\widehat{L}}_h,$ where by (4.63), ${\widehat{L}}_h:=\sigma _{r_h}({U_{4r_h}}\cap L_{k_h})$ satisfies $\mathcal {H}^1({\widehat{L}}_h)<\frac{1}{h};$
- since
  $$\begin{aligned} \int _{U_4}|e(u_h)|^2 \text {d}x \leqq \int _{U_{4r_h}}|e(w_{k_h})|^2\text {d}x \leqq \int _{B_{r_0}(0)} |e(w_{k_h})|^2\text {d}x, \end{aligned}$$
  by (4.2), we have $\sup \limits _{h\geqq 1} |e(u_h)|^2\text {d}x<\infty ;$
- by (4.62d)–(4.62e),
  $$\begin{aligned} \lim \limits _{h\rightarrow \infty } \int _{U_1} |\tau (u_h(x)) - \tau (u(x)) |\text {d}x =0, \end{aligned}$$
  thus, possibly passing to further not relabelled subsequence, $u_h\rightarrow u=u^+\chi _{U_1^+}+u^-\chi _{U_1{\setminus } U_1^+}$ a.e. in $U_1.$

This implies the claim.

Now we prove (4.40g). Given $\delta \in (0,1),$ by the continuity of $\varphi ,$ (g5) and (g6), there exists $h_\delta ^1>1$ such that

4.64 $$\begin{aligned} \mu _{k_h}(\overline{U_{r_h}}) \geqq {\widehat{\mu }}_{k_h}(\overline{U_{r_h}}) - 2\delta \mathcal {H}^1(U_{r_h}\cap (\partial A\cup \Sigma )) \end{aligned}$$

for all $h>h_\delta ^1,$ where ${\widehat{\mu }}_k$ is defined exactly the same as $\mu _k$ with $\phi$ and $g_s$ in place of $\varphi$ and $g_+(x,s),$ here we used (g5) as

$$\begin{aligned} \int _{U_{r_h}\cap \Sigma } |g_+(x,s) -g_+(0,s)|\text {d}\mathcal {H}^1 \leqq \frac{\delta }{4}\,\mathcal {H}^1(U_{r_h}\cap \Sigma ) \end{aligned}$$

provided that *h* is large enough. By Lemma 4.7, there exists $h_\delta ^2>h_\delta ^1$ such that for any $h>h_\delta ^2,$

$$\begin{aligned} \frac{1}{r_h}\,{\widehat{\mu }}_{k_h}(\overline{U_{r_h}}) \geqq 2\phi (\mathbf{e_2}) + 2g_0 -2 \delta . \end{aligned}$$

Moreover, by (2.4) and non-negativity of $g_+,$

$$\begin{aligned} \mathcal {H}^1(U_{r_h}\cap (\partial A\cup \Sigma )= & {} \mathcal {H}^1(U_{r_h}\cap \Omega \cap \partial A) + \mathcal {H}^1(U_{r_h}\cap \Sigma \cap \partial A) \\\leqq & {} \frac{\mu _{k_h}(\overline{U_{r_h}})}{c_1} + \mathcal {H}^1(U_{r_h}\cap \Sigma ). \end{aligned}$$

Thus, by (4.64) for any $h>h_\delta ^2,$

$$\begin{aligned} \Big (1+ \frac{\delta }{c_1} \Big )\,\frac{\mu _{k_h}(\overline{U_{r_h}})}{2r_h} + 2\delta \,\frac{\mathcal {H}^1(U_{r_h}\cap \Sigma )}{2r_h} \geqq \phi (\mathbf{e_2}) + g_0 - \delta . \end{aligned}$$

From here and (4.62c) we get

$$\begin{aligned} \phi (\mathbf{e_2}) + g_0 \leqq \delta + \Big (1+ \frac{\delta }{c_1} \Big )\,\Big (\frac{\mu _0(U_{r_h})}{2r_h} +\frac{r_h}{2}\Big )+2 \delta \,\frac{\mathcal {H}^1(U_{r_h}\cap \Sigma )}{2r_h}, \end{aligned}$$

therefore, first letting $h\rightarrow \infty ,$ then $\delta \rightarrow 0$, and using (g6), we obtain (4.40g).   $\quad \square$

Now we address the lower semicontinuity of $\mathcal {F}.$ We start with the following auxiliary extension result:

### Lemma 4.8

Let $$P\subset \mathbb {R}^n$$ and $$Q\supset P$$ be non-empty bounded connected Lipschitz open sets and let $$\mathcal {E}:H^1(P;\mathbb {R}^2)\rightarrow H^1(Q;\mathbb {R}^n)$$ be the Sobolev extension map, i.e., a bounded linear operator such that for any $$v\in H^1(P;\mathbb {R}^n),$$$$Ev=v$$ a.e. in $$P$$ and there exists $$C_P>0$$ such that $$\Vert \mathcal {E}v\Vert _{H^1(Q)}\leqq C_P\Vert v\Vert _{H^1(P)}.$$ Consider any $$\{u_k\}\subset H^1(P;\mathbb {R}^n)$$ such that

4.65 $$\begin{aligned} \sup \limits _{k} \int _P|e(u_k)|^2\text {d}x <\infty \end{aligned}$$

and $u_k\rightarrow u$ a.e. in $P$ for some function $u:P\rightarrow \mathbb {R}^n.$ Then there exist a subsequence $\{u_{k_l}\}_l$ and $v\in H^1(Q;\mathbb {R}^n)$ such that $v=u$ a.e. in $P$ and $\mathcal {E}u_{k_l}\rightarrow v$ in $L^2(Q)$ and

$$\begin{aligned} \sup \limits _{l} \Vert \mathcal {E}u_{k_l}\Vert _{H^1(Q)}<\infty . \end{aligned}$$

### Proof

By Proposition 3.7, $u\in H_\mathrm {loc}^1(P;\mathbb {R}^n)\cap GSBD^2(P;\mathbb {R}^n).$ By Poincaré-Korn inequality, there exist $c_P>0$ and a sequence $\{a_k\}$ of rigid displacements such that

4.66 $$\begin{aligned} \Vert u_k+a_k\Vert _{H^1(P)} \leqq c_P\Vert e(u_k)\Vert _{L^2(P)} \end{aligned}$$

for any *k*. Since $u_k\rightarrow u$ a.e. in $P$, reasoning as in the proof of Proposition 3.7 (with $P$ in place of $B_\epsilon$), up to a not relabelled subsequence, $a_k\rightarrow a$ a.e. in $\mathbb {R}^n$ for some rigid displacement $a:\mathbb {R}^n\rightarrow \mathbb {R}^n.$ In particular, $H^1(P;\mathbb {R}^n)$-norm of $a_k$ is uniformly bounded independently of *k*,  hence,

4.67 $$\begin{aligned} \sup _k \Vert \mathcal {E}a_k\Vert _{H^1(Q)}<\infty . \end{aligned}$$

Since

4.68 $$\begin{aligned} \Vert \mathcal {E}(u_k + a_k)\Vert _{H^1(Q)}\leqq C_P\Vert u_k+a_k\Vert _{H^1(P)} \leqq C_Pc_P\Vert e(u_k)\Vert _{L^2(P)}, \end{aligned}$$

by (4.65), the linearity of *E* and (4.67),

$$\begin{aligned} \sup _k \Vert \mathcal {E}u_k\Vert _{H^1(Q)}<\infty . \end{aligned}$$

Thus, by the Rellich–Kondrachov Theorem, there exists a not relabelled subsequence $$\{u_k\}$$ and $$v\in H^1(Q;\mathbb {R}^n)$$ such that $$\mathcal {E}u_k\rightarrow v$$ in $$L^2(Q)$$ and a.e. in $$Q.$$$$\quad \square $$

### Proof of Theorems 2.8

Without loss of generality, we assume that

4.69 $$\begin{aligned} \sup \limits _{k\geqq 1} \int _{A_k\cup S} |e(u_k)|^2\text {d}x + \mathcal {H}^1(\partial A_k)<\infty , \end{aligned}$$

The lower semicontinuity of the elastic-energy part can be shown by using convexity $$W(x,\cdot )$$. Indeed, let $$D\subset \subset \mathrm {Int}(A).$$ Then by $$\tau _\mathcal {A}$$-convergence of $$A_k,$$$$D\subset \subset \mathrm {Int}(A_k)$$ for all large *k*. Since $$u_k\rightarrow u$$ a.e. in $$A\cup S,$$ by (4.69) and the weak-compactness of $$L^2(D\cup S)$$, $$e(u_k)\rightharpoonup e(u)$$ in $$L^2(D\cup S).$$ Therefore, from the convexity of $$\mathcal {W}(D,\cdot )$$ it follows that

$$\begin{aligned} \mathcal {W}(D,u) \leqq \liminf \limits _{k\rightarrow \infty } \mathcal {W}(D,u_k) \leqq \liminf \limits _{k\rightarrow \infty } \mathcal {W}(A_k,u_k). \end{aligned}$$

Now letting $D\nearrow A\cup S$ we get

$$\begin{aligned} \mathcal {W}(A,u) \leqq \liminf \limits _{k\rightarrow \infty } \mathcal {W}(A_k,u_k). \end{aligned}$$

Since $\mathcal {S}(E,v) = \mathcal {S}(E,J_v;\varphi ,g)$ with $J_E=J_v$ and $g(x,s) = \beta (x)s,$ the lower semicontinuity of of the surface part, follows from Proposition 4.1 provided that for $\mathcal {H}^1$-a.e. $x\in J_u$ there exists $r_x>0$, $w_k\in GSBD(B_{r_x}(x);\mathbb {R}^2)$ and relatively open sets $L_k$ of $\Sigma$ with $\mathcal {H}^1(L_k)<1/k$ such that (4.2) holds. Let

$$\begin{aligned} r_0^x:=\frac{1}{4}\,\min \{ \mathrm {dist}(x,\partial \Omega {\setminus } \Sigma ), \mathrm {dist}(x,\partial S{\setminus } \Sigma )\} \end{aligned}$$

so that $B_{r_0^x}(x)\subset \subset \Omega \cup \Sigma \cup S,$ and choose $r=r_x\in (0,r_0^x)$ such that

$$\begin{aligned} \mathcal {H}^1(\partial B_r(x)\cap \partial A_k)= & {} \mathcal {H}^1(\partial B_r(x)\cap J_{u_k}) \\= & {} \mathcal {H}^1(\partial B_r(x)\cap \partial A) = \mathcal {H}^1(\partial B_r(x)\cap J_u) = 0 \end{aligned}$$

(see [46, Proposition 2.6]) and $$B_r(x)\cap S$$ is connected. We construct $$\{w_k\}$$ by extending $$\{u_k\}$$ in $$B_r(x){\setminus } (A_k\cup S)$$ without creating extra jumps at the interface on the exposed surface of the substrate. More precisely, we apply Lemma 4.8 with $$Q:= B_r(x),$$$$P:=B_r(x)\cap S,$$ and $$u_k\big |_P.$$ Since $$u_k\rightarrow u$$ a.e. in $$P,$$ by Lemma 4.8, there exist $$v\in H^1(Q;\mathbb {R}^2)$$ and a not relabelled subsequence $$\{u_k\}$$ such that the Sobolev extension $$\mathcal {E}u_k$$ of $$u_k\big |_P$$ to $$Q$$ converges to *v* a.e. in $$Q.$$ Define

$$\begin{aligned} w_k:=u_k\chi _{B_r(x)\cap (A_k\cup S)} + \mathcal {E}u_k\chi _{B_r(x){\setminus } (A_k\cup S)}. \end{aligned}$$

Perturbing $w_k$ slightly if necessary, we can assume $J_{w_k}=\Gamma :=B_r\cap (J_{u_k} \cup (\Omega \cup \partial ^*A_k) \cup (A_k^{(1)}\cap \partial A_k))$ up to a $\mathcal {H}^1$-negligible set. In fact, by [46, Proposition 2.6] there exist $\xi \in \mathbb {R}^2$ with arbitrarily small $|\xi |>0$ for which $\mathcal {H}^1(\{y\in \Gamma :\,\, [u_k](y)=\xi \})=0$ (with $[u_k](x)$ the size of the jump of $u_k$), and hence, we can perturb $u_k$ with a $W^{1,\infty }(B_r(x){\setminus } \Gamma )$-function with arbitrarily small norm, which is equal to $\xi$ on an arbitrarily large subset of $\Gamma .$ By construction,

$$\begin{aligned} w_k\rightarrow w:=u\chi _{B_r(x)\cap (A\cup S)} + v\chi _{B_r(x)\cap (A\cup S)}, \end{aligned}$$

thus, by [15, Theorem 1.1], $w\in GSBD^2(B_r(x);\mathbb {R}^2).$ Notice also that $J_u\subset J_w$ since $w=u$ a.e. in $B_r(x)\cap (A\cup S).$ Thus $w_k$ and *w* satisfy (4.2).   $\quad \square$

We conclude this section by proving a lower semicontinuity property of $\mathcal {F}'$ with respect to $\tau _\mathcal {C}'$. Observe that if $(A_k,u_k)\overset{\tau _\mathcal {C}'}{\rightarrow } (A,u),$ then $A =\mathrm {Int}(A)$ so that the weak convergence of $u_k$ to *u* in $H_\mathrm {loc}^1(A\cup S;\mathbb {R}^2)$ is well-defined. However, notice that $\mathcal {C}_m'$ is not closed with respect to $\tau _{\mathcal {C}}'$-convergence.

### Proposition 4.9

(Lower semicontinuity of $\mathcal {F}'$) Assume (H1)–(H3). If $(A_k,u_k)\in \mathcal {C}_m'$ and $(A,u)\in \mathcal {C}$ are such that $(A_k,u_k)\overset{\tau _{\mathcal {C}}'}{\rightarrow } (A,u),$ then

4.70 $$\begin{aligned} \liminf \limits _{k\rightarrow \infty } \mathcal {F}'(A_k,u_k) \geqq \mathcal {F}'(A,u). \end{aligned}$$

### Proof

Consider the auxiliary functional ${\widetilde{\mathcal {F}}}:\mathcal {C}\rightarrow \mathbb {R}$ defined as

$$\begin{aligned} {\widetilde{\mathcal {F}}}(A,u) = \mathcal {F}(A,u) - \int _{\Sigma \cap A^{(0)} \cap \partial A} \big (\phi (x,\nu _A) + \beta \big ) \text {d}\mathcal {H}^1. \end{aligned}$$

Since ${\widetilde{\mathcal {F}}}$ does not see wetting layer energy,

4.71 $$\begin{aligned} \mathcal {F}'(G,u) = {\widetilde{\mathcal {F}}}(G,u) - \int _\Sigma \beta \text {d}\mathcal {H}^1 = \mathcal {F}(G\cup \Sigma ,u) - \int _\Sigma \beta \text {d}\mathcal {H}^1 \end{aligned}$$

for any $G\in \mathcal {A}_m':=\{A\in \mathcal {A}:\,\,A\cup \Sigma \in \mathcal {A}_m\}.$ Repeating the proof of Theorem 2.8 one can readily show that ${\widetilde{\mathcal {F}}}$ is also $\tau _\mathcal {C}$-lower semicontinuous.

Now we prove (4.70). Without loss of generality we suppose that *liminf* is a finite limit. Let $E_k:=A_k\cup \Sigma .$ By the definition of $\mathcal {A}_m'$ and $\tau _\mathcal {C}'$-convergence, $\{E_k\}\subset \mathcal {A}_m$ and $\sup \mathcal {H}^1(\partial E_k)<\infty ,$ therefore by Proposition 3.3, there exist a (not relabelled) subsequence and $E\in \mathcal {A}_m$ such that $E_k\overset{\tau _\mathcal {C}}{\rightarrow } E.$ By Remark 2.2, $A = \mathrm {Int}(E),$ thus, by (4.71),

$$\begin{aligned} \lim \limits _{k\rightarrow \infty } \mathcal {F}'(A_k,u_k)&= \lim \limits _{k\rightarrow \infty } {\widetilde{\mathcal {F}}}(A_k\cup \Sigma ,u_k) - \int _\Sigma \beta \text {d}\mathcal {H}^1 \\&\geqq {\widetilde{\mathcal {F}}}(E,u) - \int _\Sigma \beta \text {d}\mathcal {H}^1 \geqq \mathcal {F}'(A,u). \end{aligned}$$

   $$\quad \square $$

## Existence

In this section we prove Theorems 2.6 and 2.9.

### Proof of Theorem 2.6

We start by showing the existence of solutions of problems (CP) and (UP).

For the constrained minimum problem, let $\{(A_k,u_k)\}\subset \mathcal {C}_m$ be arbitrary minimizing sequence such that

$$\begin{aligned} \sup \limits _{k\geqq 1} \mathcal {F}(A_k,u_k) <\infty . \end{aligned}$$

By Theorem 2.7, there exist $(A,u)\in \mathcal {C}_m$, a not relabelled subsequence $\{(A_k,u_k)\}$ and a sequence $\{D_k,v_k\}\subset \mathcal {C}_m$ such that $(D_k,v_k)\overset{\tau _\mathcal {C}}{\rightarrow } (A,u)$ and $|B_k|=|A_k|=\mathtt {v}$ and

$$\begin{aligned} \liminf \limits _{k\rightarrow \infty } \mathcal {F}(A_k,u_k) \geqq \liminf \limits _{k\rightarrow \infty } \mathcal {F}(D_k,v_k). \end{aligned}$$

By Lemma 3.2 (b), $D_n\rightarrow A$ in $L^1(\mathbb {R}^2)$ so that $|A| = \mathtt {v}.$ Now by Theorem 2.8

$$\begin{aligned} \inf \limits _{(V,v)\in \mathcal {C}_m,\,\,|V| = \mathtt {v}} \mathcal {F}(V,v) = \liminf \limits _{k\rightarrow \infty } \mathcal {F}(D_k,v_k)\geqq \mathcal {F}(A,u) \end{aligned}$$

so that (*A*, *u*) is a minimizer. The case of the unconstrained problem is analogous.

Now we prove (2.7). Observe that in general

5.1 $$\begin{aligned} \inf \limits _{(A,u)\in \mathcal {C}} \mathcal {F}^\lambda (A,u)\leqq \inf \limits _{(A,u)\in \mathcal {C},\,\,|A| = \mathtt {v}} \mathcal {F}(A,u) \end{aligned}$$

and the same inequality still holds if we replace $\mathcal {C}$ with $\mathcal {C}_m.$ Moreover, any solution $(A,u)\in \mathcal {C}_m$ of (UP) satisfying $|A| = \mathtt {v}$ solves also (CP). By Proposition A.6, there exists a universal constant $\lambda _0>0$ with the following property: $(A,u)\in \mathcal {C}_m$ is a solution of (CP) if and only if it solves (UP) for some (and hence for all) $\lambda \geqq \lambda _0$. Thus,

5.2 $$\begin{aligned} \inf \limits _{(A,u)\in \mathcal {C}_m} \mathcal {F}^\lambda (A,u)= \inf \limits _{(A,u)\in \mathcal {C}_m,\,\,|A| = \mathtt {v}} \mathcal {F}(A,u) \end{aligned}$$

for any $m\geqq 1$ and $\lambda \geqq \lambda _0.$ Since $\mathcal {C}_m\subset \mathcal {C}_{m+1}\subset \mathcal {C},$ the map

$$\begin{aligned} m\in \mathbb {N}\mapsto \inf \limits _{(A,u)\in \mathcal {C}_m,\,\,|A|=\mathtt {v}} \mathcal {F}(A,u) \end{aligned}$$

is nonincreasing, and

$$\begin{aligned} \inf \limits _{(A,u)\in \mathcal {C},\,\,|A|=\mathtt {v}} \mathcal {F}(A,u) \leqq \inf \limits _{(A,u)\in \mathcal {C}_m,\,\,|A|=\mathtt {v}} \mathcal {F}(A,u), \end{aligned}$$

so that

5.3 $$\begin{aligned} \inf \limits _{(A,u)\in \mathcal {C},\,\,|A|=\mathtt {v}} \mathcal {F}(A,u) \leqq \lim \limits _{m\rightarrow \infty } \inf \limits _{(A,u)\in \mathcal {C}_m,\,\,|A|=\mathtt {v}} \mathcal {F}(A,u). \end{aligned}$$

In view of (5.1) and (5.3) to conclude the proof of (2.7) it suffices to show that for any $\epsilon \in (0,1)$ and $\lambda >\lambda _0,$ there exist $n\geqq 1$ and $(E,v)\in \mathcal {C}_n$ such that

5.4 $$\begin{aligned} \inf \limits _{(A,u)\in \mathcal {C}} \mathcal {F}^\lambda (A,u) +\epsilon \geqq \mathcal {F}^\lambda (E,v) \end{aligned}$$

Indeed, by (5.4) and (5.2), given $\epsilon \in (0,1)$

$$\begin{aligned} \inf \limits _{(A,u)\in \mathcal {C}} \mathcal {F}^\lambda (A,u) +\epsilon \geqq&\mathcal {F}^\lambda (E,v)\\ \geqq&\inf \limits _{(A,u)\in \mathcal {C}_n} \mathcal {F}^\lambda (A,u)\\&=\inf \limits _{(A,u)\in \mathcal {C}_n,\,|A|=\mathtt {v}} \mathcal {F}(A,u)\\ \geqq&\lim \limits _{m\rightarrow \infty } \inf \limits _{(A,u)\in \mathcal {C}_m,\,\,|A|=\mathtt {v}} \mathcal {F}(A,u). \end{aligned}$$

Now letting $\epsilon \rightarrow 0$ and using (5.2) and (5.3) we get (2.7).

We construct $(E,v)\in \mathcal {C}_n$ satisfying (5.4) as follows. Fix $\epsilon \in (0,1)$ and $\lambda >\lambda _0,$ and choose $(A,u)\in \mathcal {C}$ such that

5.5 $$\begin{aligned} \inf \mathcal {F}^\lambda +\frac{\epsilon }{4} > \mathcal {F}^\lambda (A,u). \end{aligned}$$

Notice that:

- removing the exterior filaments decreases the energy, i.e., $\mathcal {F}(A,u) \geqq \mathcal {F}(\mathrm {Int}(A), u),$ thus, we assume that $A=\mathrm {Int}(A)$ so that *A* is open;
- let $\{A_i\}_{i\in I}$ be all open connected components of *A*. Since
  $$\begin{aligned} \mathcal {F}(A,u) = \sum \limits _{i\in I} \mathcal {F}(A_j, u), \end{aligned}$$
  by the finiteness of $\mathcal {F}(A,u)$ and |*A*|,  we can choose a finite set $I'\subset I$ such that
  $$\begin{aligned} |A| \leqq \sum \limits _{j\in I'} |A_j| + \frac{\epsilon }{8\lambda }. \end{aligned}$$
  Thus, setting $A':=\bigcup _{j\in I'} A_j$ and $u':=u\big |_{A'}$ we get that $(A',u')\in \mathcal {C}$ and
  5.6 $$\begin{aligned} \mathcal {F}^\lambda (A,u) + \frac{\epsilon }{8} \geqq \mathcal {F}(A',u'); \end{aligned}$$
- let $\{F_j\}_{j\in J}$ be all open connected components of $\Omega {\setminus } \overline{A'}.$ Since $\partial F_j\subset \partial A'\cup \partial \Omega$ and $|\Omega | + \mathcal {H}^1(\partial A') + \mathcal {H}^1(\partial \Omega )<\infty ,$ there exists a finite set $J'\subset J$ such that
  $$\begin{aligned} \sum \limits _{j\in J{\setminus } J'} \mathcal {S}(F_j;u_0) <\frac{\epsilon }{16} \end{aligned}$$
  and
  $$\begin{aligned} \sum \limits _{j\in J{\setminus } J'} |F_j| <\frac{\epsilon }{16\lambda }. \end{aligned}$$
  Hence, setting $A'':= A\cup \bigcup _{j\in J{\setminus } J'} F_j$ and $u'':=u'\chi _{A'} +u_0\chi _{\bigcup _{j\in J{\setminus } J'} F_j}$ we get that $(A',u')\in \mathcal {C}$ and
  5.7 $$\begin{aligned} \mathcal {F}^\lambda (A',u')+\frac{\epsilon }{4} \geqq \mathcal {F}^\lambda (A'',u''). \end{aligned}$$
  Notice that for $j\in J{\setminus } J',$ the set $\partial A'\cap \partial F_j$ becomes the internal crack for $A'',$ and there is no elastic energy contribution in $F_j;$

see Fig. 2.

Hence, $A''$ is a union of finitely many connected open sets with finitely many “holes” inside so that $\partial \overline{A''} = \overline{\partial ^*A''}$ consists of finitely many connected sets with finite length. Moreover, by (5.5), (5.6) and (5.7),

5.8 $$\begin{aligned} \inf \mathcal {F}^\lambda +\frac{\epsilon }{2} \geqq \mathcal {F}^\lambda (A'',u''). \end{aligned}$$

In view of (5.5) and (5.8) it remains to show that there exists $m\geqq 1$ and $(E,v)\in \mathcal {C}_m$ such that

5.9 $$\begin{aligned} \mathcal {F}^\lambda (A'',u'') + \frac{\epsilon }{2} > \mathcal {F}^\lambda (E,v). \end{aligned}$$

Let $G:=\mathrm {Int}(\overline{ A'' })$ so that *G* is open and $\partial G =\overline{\partial ^*G}.$ Since $\Sigma$ is a 1-dimensional Lipschitz manifold, by the outer regularity of  there exists a finite union *I* of subintervals of $\Sigma$ such that

$$\begin{aligned} J_{ u'' }\subseteq I \end{aligned}$$

and

5.10 $$\begin{aligned} \mathcal {H}^1( I {\setminus } J_{ u'' }) < \frac{\epsilon }{64c_2}. \end{aligned}$$

Since $\partial \Omega$ is Lipschitz and $\varphi$, there exists a Lipschitz open set $V\subset \Omega$ such that $\partial V\cap \Sigma = I$ and

5.11 $$\begin{aligned} \int _{\Omega \cap \partial ^* V} \varphi (x,\nu _U)\text {d}\mathcal {H}^1 \leqq \int _{I} \varphi (x,\nu _\Sigma )\text {d}\mathcal {H}^1 + \frac{\epsilon }{64} \end{aligned}$$

and

5.12 $$\begin{aligned} |V| < \frac{\epsilon }{32\lambda }; \end{aligned}$$

since $\varphi$ is uniformly continuous, basically, *V* is obtained slightly translating *I* inside *A*.

Let us consider $(B,w)\in \mathcal {C}$ with $B:= A'' {\setminus } V$ and $w= u'' \chi _{ A'' {\setminus } V}^{}.$ Since $B \subset A'',$

$$\begin{aligned} \mathcal {W}( B , w ) \leqq \mathcal {W}( A'' , u'' ); \end{aligned}$$

since $J_{ w }=\emptyset ,$ by (5.10) and (5.11)

5.13 $$\begin{aligned} \mathcal {S}( B , w ) \leqq \mathcal {S}( A'' , u'' ) +\frac{\epsilon }{16}, \end{aligned}$$

and by (5.12)

$$\begin{aligned} \lambda \big || B | - \mathtt {v}\big | \leqq \lambda \big || A'' | - \mathtt {v}\big | +\frac{\epsilon }{16}. \end{aligned}$$

Thus,

5.14 $$\begin{aligned} \mathcal {F}^\lambda ( B , w ) \leqq \mathcal {F}^\lambda ( A'' , u'' ) +\frac{\epsilon }{8}. \end{aligned}$$

Let ${\widetilde{w}}\in GSBD^2(\mathrm {Int}(\overline{ B \cup S});\mathbb {R}^2)$ be such that ${\widetilde{w}}= w$ a.e. in $\overline{ B \cup S}.$ Notice that $\Sigma \cap J_{ {\widetilde{w}} }=\emptyset$ and $J_{ {\widetilde{w}} }\subseteq { B }^{(1)}\cap \partial B .$ Perturbing approximate continuity points of *w* along $B ^{(1)}\cap \partial B$ (as has been done in the proof of Theorem 2.8), we may suppose that $B ^{(1)}\cap \partial B$ is a jump set for ${\widetilde{w}} .$ Hence, using the Vitali class of covering squares for $J_{ {\widetilde{w}} }$ contained in $\Omega$ in the proof of [14, Theorem 1.1] we find ${\widetilde{v}}\in SBV^2(\mathrm{Int}{( B )\cup S\cup \Sigma )};\mathbb {R}^2)\cap W^{1,\infty }(\mathrm{Int}{( B \cup S\cup \Sigma )};\mathbb {R}^2)$ such that $J_{{\widetilde{v}}}$ is contained in a union of finitely many closed connected curves in $\overline{ B }$ (see [14, pp. 1353 and 1359]) and

5.15a $$\begin{aligned}&\int _{ B \cup S}|e({\widetilde{v}}) -e( {\widetilde{w}} )|^2\text {d}x <\frac{\epsilon ^2}{512(\mathcal {W}( B , {\widetilde{w}} ) + 1)(\Vert \mathbb {C}\Vert _\infty + 1)}, \end{aligned}$$

5.15b $$\begin{aligned}&\mathcal {H}^1(J_{{\widetilde{v}}}\Delta J_{ {\widetilde{w}} }) <\frac{\epsilon }{32c_2}. \end{aligned}$$

Notice that we do not need to control the boundary trace of ${\widetilde{w}}$ that’s why we can use the approximation result [14, Theorem 1.1] only inside $B\cup \Sigma \cup S.$ Moreover, since $J_{{\widetilde{w}}}\subset \mathrm {Int}(\overline{B})$ and we use Vitali class of covering cubes only inside $\Omega$ by the formula [14, page 1359] for the jump of the approximating sequence, it follows that $J_{{\widetilde{v}}}\subset \overline{B}.$ In particular, ${{\widetilde{v}}}\in H^1(S;\mathbb {R}^2).$

By the convexity of the elastic energy and the Cauchy-Schwarz inequality for nonnegative quadratic forms,

5.16 $$\begin{aligned}&\int _{ B \cup S} W(x,e({\widetilde{v}}) - E_0)\text {d}x \leqq \int _{ B \cup S} W(x,e( {\widetilde{w}} )- E_0)\text {d}x\nonumber \\&\qquad +2\int _{ B \cup S} \nonumber \mathbb {C}(x)[e({\widetilde{v}})- E_0]:[e({\widetilde{v}}) - e( {\widetilde{w}} )]\text {d}x\nonumber \\&\quad \leqq \int _{ B \cup S} W(x,e( {\widetilde{w}} )- E_0)\text {d}x\nonumber \\&\qquad +2\sqrt{\int _{ B \cup S} W(x,e({\widetilde{v}})- E_0)\text {d}x} \sqrt{\int _{ B \cup S} W(x,e({\widetilde{v}}) - e( {\widetilde{w}} ))\text {d}x}. \end{aligned}$$

Since

$$\begin{aligned} \int _{ B \cup S} W(x,e({\widetilde{v}}) - e( {\widetilde{w}} ))\text {d}x\leqq \Vert \mathbb {C}\Vert _\infty \int _{ B \cup S} |e({\widetilde{v}}) - e( {\widetilde{w}} )|^2\text {d}x, \end{aligned}$$

and

$$\begin{aligned}&\int _{ B \cup S} W(x,e({\widetilde{v}})- E_0)\text {d}x \\&\quad \leqq 2\int _{ B \cup S} W(x,e({\widetilde{v}})- e( {\widetilde{w}} ))\text {d}x + 2\int _{ B \cup S} W(x,e( {\widetilde{w}} )-E_0)\text {d}x\\&\quad \leqq 2\Vert \mathbb {C}\Vert _\infty \int _{ B \cup S} |e({\widetilde{v}})- e( {\widetilde{w}} )|^2\text {d}x +2 \mathcal {W}( B , {\widetilde{w}} ) \leqq 2 \mathcal {W}( B , {\widetilde{w}} )+ 2, \end{aligned}$$

by (5.15a) and (5.16),

5.17 $$\begin{aligned} \int _{ B \cup S} W(x,e({\widetilde{v}})- E_0)\text {d}x \leqq \int _{ B \cup S} W(x,e( {\widetilde{w}} ) - E_0)\text {d}x + \frac{\epsilon }{4}. \end{aligned}$$

As $J_v$ is contained in at most finitely many closed $C^1$-curves, we can find finitely many arcs of those curves whose union $\Gamma \subset \overline{ B }$ still contains $J_{{\widetilde{v}}}$ and satisfies

5.18 $$\begin{aligned} \mathcal {H}^1(\Gamma {\setminus } J_{{\widetilde{v}}})<\frac{\epsilon }{32c_2}. \end{aligned}$$

Set $$E: = \mathrm {Int}(\overline{ B }){\setminus } \Gamma $$ and $$v:={\widetilde{v}}\big |_{E}.$$ We show that (*E*, *v*) satisfies (5.9). Note that $$J_v\cap (E\cup S)=\emptyset ,$$ thus, $$v\in H_\mathrm {loc}^1(E\cup S;\mathbb {R}^2)\cap GSBD^2(\mathrm{Int}{(E\cup S\cup \Sigma )};\mathbb {R}^2).$$ Moreover, by construction, $$\overline{\partial ^*A''},$$$$\Gamma $$ and $$\partial V$$ consist of finitely many connected components, therefore, there exists $$m\geqq 1$$ such that $$(E,v)\in \mathcal {C}_m.$$ Notice that by the definition of *E*, 

5.19 $$\begin{aligned} |E| = |\mathrm {Int}(\overline{ B })| = | B |, \end{aligned}$$

by the definition of *v*,  ${\widetilde{w}}$ and (5.17),

5.20 $$\begin{aligned} \mathcal {W}(E, v) \leqq \mathcal {W}( B , {\widetilde{w}} ) + \frac{\epsilon }{4}=\mathcal {W}( B, w ) + \frac{\epsilon }{4}, \end{aligned}$$

and by $\partial ^*E=\partial ^*B,$ (5.18) and (5.15b) as well as (2.4) and (5.13),

5.21 $$\begin{aligned} \mathcal {S}(E, v) \leqq&\mathcal {S}( B , w ) + \frac{\epsilon }{8}. \end{aligned}$$

From (5.19)–(5.21) we get

$$\begin{aligned} \mathcal {F}^\lambda (E,v) \leqq \mathcal {F}^\lambda (B,w) +\frac{3\epsilon }{8}. \end{aligned}$$

Combining this with (5.14) we obtain (5.9).   $\quad \square$

### Proof of Theorem 2.9

In view of Proposition 4.9 the assertion follows from the direct methods of the Calculus of Variations.   $\quad \square$

## Appendix A

In this section we recall some results from the literature for the reader’s convenience.

### Kuratowski Convergence

Let $\{E_k\}$ be a sequence of subsets of $\mathbb {R}^2.$ A set $E\subset \mathbb {R}^2$ is the $\mathcal {K}$-*lower limit* of $\{E_k\}$ if for every $x\in E$ and $\rho >0$ there exists $n>0$ such that $B_\rho (x)\cap E_k \ne \emptyset$ for all $k>n.$ A set $E\subset \mathbb {R}^2$ is the $\mathcal {K}$-*upper limit* of $\{E_k\}$ if for every $x\in E$ and $\rho >0$ and $n\in \mathbb {N}$ there exists $k>n$ such that $B_\rho (x)\cap E_k \ne \emptyset .$

The $\mathcal {K}$-lower and $\mathcal {K}$-upper limits of $\{E_k\}$ are always exist and respectively denoted as

$$\begin{aligned} \mathcal {K}-\liminf \limits _{k\rightarrow \infty } E_k \qquad \text {and} \qquad \mathcal {K}-\limsup \limits _{k\rightarrow \infty } E_k. \end{aligned}$$

It is clear that both sets are closed sets and

$$\begin{aligned} \mathcal {K}-\liminf \limits _{k\rightarrow \infty } E_k \subseteq \mathcal {K}-\limsup \limits _{k\rightarrow \infty } E_k; \end{aligned}$$

in case of equality, we say $E_k$ converges to $E = \mathcal {K}-\liminf \limits _{k\rightarrow \infty } E_k = \mathcal {K}-\limsup \limits _{k\rightarrow \infty } E_k$ in the Kuratowski sense and write

$$\begin{aligned} E= \mathcal {K}-\lim \limits _{k\rightarrow \infty } E_k\qquad \text {or} \qquad E_k\overset{\mathcal {K}}{\rightarrow }E. \end{aligned}$$

Observe that by the definition of $\mathcal {K}$-convergence, $E_k$ and $\overline{E_k}$ have the same $\mathcal {K}$-upper and $\mathcal {K}$-lower limits. Moreover, Kuratowski limit is always unique.

#### Proposition A.1

The following assertions are equivalent:

- $$E_k\overset{\mathcal {K}}{\rightarrow }E;$$
- if $x_k\in E_k$ converges to some $x\in \mathbb {R}^2,$ then $x\in E,$ and for every $x\in E$ there exists a subsequence $x_{n_k}\in E_{n_k}$ converging to *x*; 
- $\mathrm {dist}(\cdot ,E_k) \rightarrow \mathrm {dist}(\cdot , E)$ locally uniformly in $\mathbb {R}^2;$
- if, in addition, $\{E_k\}$ is uniformly bounded, $E_k\rightarrow E$ with respect to Hausdorff distance $\mathrm {dist}_{\mathcal {H}},$ where
  A.1 $$\begin{aligned} \mathrm {dist}_{\mathcal {H}}(A,B):= {\left\{ \begin{array}{ll} 0 &{} \hbox { if}\ A=B=\emptyset ,\\ \max \Big \{\sup \limits _{x\in A} \mathrm {dist}(x, B), \, \sup \limits _{x\in B} \mathrm {dist}(x, A)\Big \} &{}\text {otherwise}. \end{array}\right. } \end{aligned}$$

### Rectifiability in $\mathbb {R}^2$

Below we recall some important regularity properties of compact connected subsets of finite $\mathcal {H}^1$-measure of $\mathbb {R}^2$ most of them are taken from [29, Chapters 2 and 3].

The image $\Gamma$ of a continuous injection $\gamma :[a,b]\rightarrow \mathbb {R}^2$ is called *curve* (or *Jordan curve*), and $\gamma$ is the parametrization of $\Gamma .$ Clearly, any curve is compact and connected set, hence it is $\mathcal {H}^1$-measurable. The length of a curve $\Gamma$ is defined as

$$\begin{aligned} \sup s(\gamma , P), \end{aligned}$$

where $P=\{t_0,t_1,\ldots ,t_N\}$ is a partition of [*a*, *b*],  i.e., $a=t_0<t_1<\ldots <t_N=b,$

$$\begin{aligned} s(\gamma , P):= \sum \limits _{i=1}^N |\gamma (t_{i-1}) - \gamma (t_i)|, \end{aligned}$$

and $\sup$ is taken over all partitions *P* of [*a*, *b*]. By [29, Lemma 3.2], the length of curve $\Gamma$ is equal to $\mathcal {H}^1(\Gamma ).$

Any curve $\Gamma$ with finite length admits so-called *arclength* parametrization in $[0,\mathcal {H}^1(\Gamma )],$ which is a Lipschitz parametrization $\gamma _o$ with Lipschitz constant 1. Hence, by the Rademacher Theorem [3, Theorem 2.14] it is differentiable at a.e. $\ell \in (0,\mathcal {H}^1(\Gamma ))$ and $|\dot{\gamma _o}(\ell )|\leqq 1.$ Hence $\Gamma$ has an (approximate) tangent line at $\mathcal {H}^1$-a.e. $x\in \Gamma$ and we can define the approximate unit normal $\nu _\Gamma (x)$ of $\Gamma$ at $\mathcal {H}^1$-a.e. $x\in \Gamma .$

We recall the following characteristics of compact connected $\mathcal {H}^1$-rectifiable sets (see [29, Theorem 3.14] and [36, Section 2]).

#### Proposition A.2

(Rectifiable connected sets) Every connected compact set $K\subset \overline{U}$ with $\mathcal {H}^1(K)<\infty$ is arcwise connected and countably $\mathcal {H}^1$-rectifiable, i.e,

$$\begin{aligned} K:= N\cup \bigcup \limits _{j\geqq 1} \Gamma _j, \end{aligned}$$

where *N* is a $\mathcal {H}^1$-negligible set and $\Gamma _j:=\gamma _j([0,1])$ is a curve with finite length for a parametrization $\gamma _j:[0,1]\rightarrow \mathbb {R}^2$ such that

$$\begin{aligned} \gamma _j((0,1))\cap \bigcup \limits _{i=1}^{j-1}\Gamma _i = \emptyset ,\qquad j\geqq 2. \end{aligned}$$

#### Remark A.3

(*Rectifiable curve is locally Jordan*) Let $\Gamma$ be a rectifiable curve. Then for $\mathcal {H}^1$-a.e. $x\in \Gamma$ there exists $r_x>0$ such that $B_{r_x}(x){\setminus } \Gamma$ has exactly two connected components. Indeed, suppose that there exists $x\in \Gamma$ such that $\theta ^*(\Gamma ,x) = \theta _*(\Gamma ,x)=1$ and $B_r(x){\setminus }\Gamma$ has at least three connected components for every $r>0$ such that endpoints of $\Gamma$ lie outside $\overline{B_r(x)}.$ Then $(B_r(x){\setminus } B_{r/2}(x))\cap \Gamma$ should have three connected components and as a result $\mathcal {H}^1((B_r(x){\setminus } B_{r/2}(x))\cap \Gamma )\geqq 3r/2$ and

$$\begin{aligned} 1 =\lim \limits _{r\rightarrow 0} \frac{\mathcal {H}^1(B_r(x)\cap \Gamma )}{2r} \geqq \lim \limits _{r\rightarrow 0} \frac{\mathcal {H}^1(B_{r/2}(x)\cap \Gamma )}{2r} +\frac{3}{4} = \frac{5}{4}, \end{aligned}$$

which is a contradiction.

#### Proposition A.4

(Properties of regular points [3, 29, 36]) Suppose that $K\subset \mathbb {R}^2$ be a connected compact set with $\mathcal {H}^1(K)<\infty .$ Thus, it admits a unit (measure-theoretic) normal $\nu _K(x)$ at $\mathcal {H}^1$-a.e. $x\in K;$ the map $x\mapsto \nu _K(x)$ is Borel measurable and if *L* is any connected subset of *K* then $\nu _L(x) = \nu _K(x)$ for any $x\in L$ for which the unit normal $\nu _K(x)$ to *K* exists. Moreover,  and $\overline{U_{1,\nu _K}(x)}\cap \sigma _{\rho ,x}(K) \overset{\mathcal {K}}{\rightarrow } \overline{U_{1,\nu _K}(x)}\cap T_x$ as $\rho \rightarrow 0^+,$ where $T_x$ is the generalized tangent line to *K* at *x*.

Note that $U_{1,\nu _K}(x)$ in Proposition A.4 can be replaced by arbitrary $U_{R,\nu _K}(x)$.

#### Proposition A.5

Let $A\in \mathcal {A}$ and $x\in \partial A$ be such that the measure-theoretic unit normal $\nu _A(x)$ to $\partial A$ exists and $\overline{U_{R,\nu _A(x)}(x)}\cap \sigma _{\rho ,x}(\partial A) \overset{\mathcal {K}}{\rightarrow } \overline{U_{R,\nu _A(x)}(x)}\cap T_x$ for any $R>0$ as $\rho \rightarrow 0^+.$ Then:

- if $x\in A^{(1)}\cap \partial A,$ then $\mathrm {sdist}(\cdot ,\sigma _{\rho ,x}(\partial A))\rightarrow -\mathrm {dist}(\cdot ,T_x)$ uniformly in $\overline{U_{1,\nu _A(x)}};$
- if $x\in A^{(0)}\cap \partial A,$ then $\mathrm {sdist}(\cdot ,\sigma _{\rho ,x}(\partial A))\rightarrow \mathrm {dist}(\cdot ,T_x)$ uniformly in $\overline{U_{1,\nu _A(x)}}.$

#### Proof

We prove only (a); the proof of (b) is analogous. Let $x\in A^{(1)}\cap \partial A$ be such that

1. $$\nu _A(x)$$ exists;
2. $\overline{U_{R,\nu _A(x)}(x)}\cap \sigma _{\rho ,x}(\partial A) \overset{\mathcal {K}}{\rightarrow } \overline{U_{R,\nu _A(x)}(x)}\cap T_x$ for any $R>0$ as $\rho \rightarrow 0^+.$

Without loss of generality we assume $$x=0,$$$$\nu _A(x)=\mathbf{e_2}$$ and $$T_x=T_0$$ is the $$x_1$$-axis. For shortness we write $$A_\rho $$ and $$(\partial A)_\rho $$ in places of $$\sigma _{\rho ,x}(A)$$ and $$\sigma _{\rho ,x}(\partial A),$$ respectively. Let $$\{\rho _k\}\subset (0,1)$$ be arbitrary sequence converging to 0. Consider $$f_k:=\mathrm {sdist}(\cdot ,(\partial A)_{\rho _k})\big |_{U_4}\in W^{1,\infty }(U_4).$$ For any $$k\geqq 1,$$$$f_k$$ is 1-Lipschitz and $$f_k(0)=0,$$ therefore, by the Arzela-Ascoli Theorem, there exist $$f\in W^{1,\infty }(U_4)$$ and not relabelled subsequence $$\{f_k\}$$ such that $$f_k\rightarrow f$$ uniformly in $$U_4.$$ By (x2) applied with $$R>4$$, $$|f_k| = \mathrm {dist}(\cdot ,(\partial A)_{\rho _k})\rightarrow \mathrm {dist}(\cdot ,T_0)$$ uniformly in $$U_4,$$ therefore, $$|f(x)| = \mathrm {dist}(x, T_0)$$ for any $$x\in U_4.$$ Thus, it suffices to prove that $$f\leqq 0.$$

Assume by contradiction that $f>0$ in $U_4^+:= U_4\cap \{x_2>0\}.$ In addition, by (x2) for any $\delta \in (0,1)$ there exists $k_\delta >0$ such that $U_4\cap (\partial A)_{\rho _k}\subset T_0\times (-\delta ,\delta )$ for any $k>k_\delta .$ Therefore, $\mathrm {sdist}(\cdot ,(\partial A)_{\rho _k})>0$ in $U_{4,\delta }^+:=U_4\cap \{x_2>\delta \},$ and hence, $A\cap \rho _k U_{4,\delta }^+ = \emptyset ,$ where $r D = \{rx:\,x\in D\}.$ Since $0\in A^{(1)}\cap \partial A,$ this implies

$$\begin{aligned} 1=\lim \limits _{k\rightarrow \infty } \frac{|A\cap U_{4\rho _k}|}{|U_{4\rho _k}|} = \lim \limits _{k\rightarrow \infty } \frac{|(A\cap U_{4\rho _k}){\setminus } \rho _kU_{4,\delta }^+|}{|U_{4\rho _k}|} < 1, \end{aligned}$$

which is a contradiction.

An analogous contradiction is obtained assuming $$f>0$$ in $$U_4\cap \{x_2<0\}.$$$$\quad \square $$

### Minimizers of Volume-Constraint and Unconstraint Problems

The following proposition is an extension of [28, Theorem 1.1].

#### Proposition A.6

Assume (H1)–(H3). Then there exists $$\lambda _0>0$$ (possibly depending on $$c_1,$$$$c_2$$ and $$\Omega $$) with the following property: given $$m\geqq 1,$$$$(A,u)\in \mathcal {C}_m$$ is a solution of (CP) if and only if (*A*, *u*) is also a solution to (UP) for all $$\lambda \geqq \lambda _0.$$

#### Proof

Note that any minimizer $(A,u)\in \mathcal {C}_m$ of $\mathcal {F}^\lambda$ with $|A|=\mathtt {v}$ is also minimizer of $\mathcal {F}.$ Hence, it suffices to show that there exists $\lambda _0>0$ such that any minimizer (*A*, *u*) of $\mathcal {F}^\lambda$ for $\lambda >\lambda _0$ satisfies $|A|=\mathtt {v}.$

Assume by contradiction that there exist sequences $\{m_h\}\subset \mathbb {N}$ and $\{\lambda _h\}\subset \mathbb {R}$ with $\lambda _h\rightarrow \infty$ and a sequence $(A_h,u_h)\in \mathcal {C}_{m_h}$ minimizing $\mathcal {F}^{\lambda _h}$ such that $|A_h|\ne \mathtt {v}.$ Since $\Omega$ has finitely many connected components there exists an open Lipschitz set $A_0\subset \Omega$ with $|A_0|=\mathtt {v}$ such that $\mathcal {F}^{\lambda _h}(A_h,u_h) \leqq \mathcal {F}^{\lambda _h}(A_0,u_0)=\mathcal {F}(A_0,u_0)$ for all large *h*. Thus, by (2.4) and (2.5),

A.2 $$\begin{aligned} \sup \limits _h \mathcal {H}^1(\partial A_h) \leqq \Lambda :=\frac{\mathcal {F}(A_0,u_0) + c_2\mathcal {H}^1(\Sigma ) + \mathcal {H}^1(\partial \Omega )}{c_1}, \end{aligned}$$

and $\lambda _h||A_h| - \mathtt {v}|\leqq \mathcal {F}(A_0,u_0) + c_2\mathcal {H}^1(\Sigma )$ for any *h*. This implies $|E_h| \rightarrow \mathtt {v}$ as $h\rightarrow \infty .$ By compactness, there exists a finite perimeter set $A\subset \Omega$ and a not relabelled subsequence such that $\chi _{A_h} \rightarrow \chi _A$ a.e. in $\mathbb {R}^2.$ In particular, $|A|=\mathtt {v}.$

We suppose that $|A_h|<\mathtt {v}$ for all *h*;  the case $|A_h|>\mathtt {v}$ is treated analogously. As in the proof of [28, Theorem 1.1] given $\epsilon \in (0,\frac{1}{8})$, there exist small $r>0$ and $x_r\in \Omega$ such that $B_r(x)\subset \subset \Omega$ and

$$\begin{aligned} |A\cap B_{r/2}(x_r)|<\epsilon r^2,\qquad |A\cap B_r(x_r)|>\frac{\pi r^2}{16}. \end{aligned}$$

For shortness, we suppose that $x_r=0$ we write $B_r:=B_r(x_r).$ Since $A_h\rightarrow A$ in $L^1(\mathbb {R}^2),$ for all large *h*, 

A.3 $$\begin{aligned} |A_h\cap B_{r/2}|<\epsilon r^2,\qquad |A_h\cap B_r|>\frac{\pi r^2}{16}. \end{aligned}$$

Let $\Phi :\mathbb {R}^2\rightarrow \mathbb {R}^2$ be the bi-Lipschitz map which takes $B_r$ into $B_r$ defined as

$$\begin{aligned} \Phi (x):= {\left\{ \begin{array}{ll} (1 - 3\sigma )x, &{} |x|<\frac{r}{2},\\ x+ \sigma \Big (1 - \frac{r^2}{|x|^2}\Big )x, &{} \frac{r}{2} \leqq x < r,\\ x, &{} |x|\geqq r \end{array}\right. } \end{aligned}$$

for some $\sigma \in (0,\frac{1}{16}).$ Recall from [28, pp. 420-422] that the Jacobian $J\Phi$ of $\Phi$ satisfies

$$\begin{aligned} J\Phi (y) \geqq 1+ \sigma \qquad y\in B_r{\setminus } B_{r/2}, \end{aligned}$$

and

$$\begin{aligned} J\Phi (y) \leqq 1+ 8\sigma \qquad y\in B_r, \end{aligned}$$

and the tangential Jacobian $J_1T_x$ of $\Phi$ on the tangent space $T_x$ of $\partial A_h$ satisfies

A.4 $$\begin{aligned} J_1T_x \leqq 1+5\sigma ,\qquad x\in B_r \cap \partial A_h. \end{aligned}$$

Set

$$\begin{aligned} E_h:= \Phi (A_h){\setminus } \partial B_r,\qquad v_h: =u_h\chi _{A_h}{\setminus } B_r + u_0\chi _{E_h\cap B_r}. \end{aligned}$$

Note that $|E_h|<\mathtt {v}$ and since the bi-Lipschitz maps do not increase the number of connected components, $(E_h,v_h)\in \mathcal {C}_m.$ Let us estimate

$$\begin{aligned}&\mathcal {F}^{\lambda _h}(A_h,u_h) - \mathcal {F}^{\lambda _h}(E_h,v_h)\\ {}&\quad = \int _{\overline{B_r}\cap \partial A_h} \theta _{A_h}(x)\,\phi (x,\nu _{A_h}) \text{ d } \mathcal {H}^1 - \int _{\overline{B_r}\cap \partial E_h} \theta _{E_h}(x) \phi (x,\nu _{E_h})\text{ d }\mathcal {H}^1\\ {}&\qquad + \int _{B_r\cap A_h} W(x,e(u_h) - E_0)\text{ d }x - \int _{B_r\cap E_h} W(x,e(v_h) - E_0)\text{ d }x \\ {}&\qquad + \lambda _h \Big (|E_h| - |A_h|\Big ) := I_1 + I_2 + I_3, \end{aligned}$$

where $$\theta _F(x)$$ is 1 for $$\mathcal {H}^1$$-a.e. on $$\partial ^*F,$$ is 2 for $$\mathcal {H}^1$$-a.e. on $$F^{(1)}\cup F^{(0)}\cap \partial F$$ and is 0 otherwise. By the choice of $$v_h,$$$$I_2\geqq 0.$$ Moreover, by (A.4) and the area formula as well as from (2.4), (A.2) and equality $$\theta _{E_h}(\Phi (y)) = \theta _{A_h}(y)$$ for $$\mathcal {H}^1$$-a.e. $$y\in \partial A_h$$,

$$\begin{aligned} \int _{B_r\cap \partial E_h}&\theta _{E_h}(x) \phi (x,\nu _{E_h})\text {d}\mathcal {H}^1 = \int _{B_r\cap \partial A_h} \theta _{A_h}(\Phi (y))\, \phi (\Phi (y),\nu _{A_h})J_1 T_y\,\text {d}\mathcal {H}^1\\&\leqq 2c_2(1+5\sigma ) \mathcal {H}^1(B_r\cap \partial A_h) \leqq 2c_2(1+5\sigma )\Lambda . \end{aligned}$$

Moreover, by (2.4),

$$\begin{aligned} \int _{\partial B_r\cap \partial E_h} \theta _{E_h}(x) \phi (x,\nu _{E_h})\text {d}\mathcal {H}^1 \leqq 2c_2\mathcal {H}^1(\partial B_r) \leqq 4\pi c_2r, \end{aligned}$$

thus,

$$\begin{aligned} I_1\geqq - 2c_2(1+5\sigma )\Lambda - 4\pi c_2r. \end{aligned}$$

Finally, repeating the same arguments of Step 4 in the proof of [28, Theorem 1.1], we obtain

$$\begin{aligned} I_3 \geqq \lambda _h \sigma r^2 (1 - 7\epsilon ), \end{aligned}$$

thus,

A.5 $$\begin{aligned} \mathcal {F}^{\lambda _h}(A_h,u_h) - \mathcal {F}^{\lambda _h}(E_h,v_h) \geqq \lambda _h \sigma r^2 (1 - 7\epsilon ) - 2c_2(1+5\sigma )\Lambda -4\pi c_2r.\nonumber \\ \end{aligned}$$

Since the dependence of the right-side of (A.5) on *h* is only through $$\lambda _h,$$ for sufficiently large *h* we have $$\mathcal {F}^{\lambda _h}(A_h,u_h) >\mathcal {F}^{\lambda _h}(E_h,v_h),$$ which contradicts to the minimality of $$(A_h,u_h).$$$$\quad \square $$

## Footnotes

1 If $\mathbb {R}^2{\setminus } A_k \overset{K}{\rightarrow }\mathbb {R}{\setminus } \mathrm {Int}(A),$ then for any $x\in \mathrm {Int}(A)$ one has $x\in \mathrm {Int}(A_n)$ for all large *n*.
